# Supplementary material for: Patterns and drivers of diatom diversity and abundance in the global ocean
Source: Nat Commun. 2025 Apr 11;16:3452. doi: 10.1038/s41467-025-58027-7 (PMC11992049; doi:10.1038/s41467-025-58027-7)
Supplement: Supplementary file 1 — Supplementary Information [file 41467_2025_58027_MOESM1_ESM.pdf]

Supplementary Figures and Tables

Patterns and drivers of diatom diversity and abundance in the global ocean
Pierella Karlusich, et al. 2025 *Nat Comm*

Table of contents

Table S1: Estimated ASV richness for V4 and V9 datasets using multiple biodiversity estimators.
Table S2: Partial least square analyses to explore the correlations between diatom relative abundance and Shannon index with the physicochemical context.
Table S3: Partial least square analyses to explore the correlations between diatom relative abundance and biotic factors.

Figure S1: Biogeographical coverage of the *Tara* Oceans datasets used in the current work.
Figure S2: Samples and methods used in this study.
Figure S3: Contribution of diatoms to eukaryotic phytoplankton and silicifiers in surface waters of the global ocean using V4 metabarcoding data obtained from different size-fractions.
Figure S4: Accumulation curves for V4 and V9 18S rRNA gene metabarcoding assigned to diatoms.
Figure S5: Distribution of diatom relative abundances in epipelagic seawater samples from the V9 and V4 metabarcoding data across size fractions, water layers, ocean basins, and biomes.
Figure S6: Comparison between V4 and V9 metabarcoding for the global macroecological patterns of diatom relative abundance and diversity in surface waters using data obtained from different size-fractions.
Figure S7: Abundance patterns of diatom classes based on the V4 marker.
Figure S8: Relative contribution of the different diatom classes.
Figure S9: Biogeography of diatom classes in surface waters using V4 and V9 metabarcoding data obtained from different size-fractionated samples.
Figure S10: Environmental distribution of the *Attheya* genus.
Figure S11: Global distribution of the 20 most abundant genera in the V9 and V4 datasets.
Figure S12: Taxonomic composition of the diatom ASV modules obtained by WGCNA.
Figure S13: Unassigned ASVs in the *Tara* Oceans V4 and V9 metabarcoding datasets from diatoms.
Figure S14: Phylogenetic distribution of DUF285 (PF03382) in reference proteomes from UniprotKB database.
Figure S15: Latitudinal abundance gradient for genes and transcripts coding for Domain of Unknown Function 285 (DUF285) and Heat Shock Protein 90 (HSP90) in diatoms and other eukaryotic phytoplankton.

Table S1: Estimated ASV richness for V4 and V9 datasets using multiple biodiversity estimators.

Marker	Observed ASV count	Chao1 estimator	Chao1 standard error	First-order Jackknife estimator	First-order Jackknife standard error	Second-order Jackknife estimator	Bootstrap estimator	Bootstrap standard error	Total number of observations
V9	34243424	3424.899	1.040739	3441.981	4.238084	3280.522	3472.61	25.21701	931
V4	3429	3429.878	1.043834	3443.985	4.791743	3331.35	3473.272	16.97236	969

Table S2: Partial least square analyses to explore the correlations between diatom relative abundance and Shannon index with the physicochemical context

	Diatom relative abundance		*Diatom Shannon*	
	VIP score	*Standard coefficient*	*VIP score*	*Standard coefficient*
Temperature	**1.6266765279**	-0.18715260	**1.5764379**	0.14211408
NH_4^+/DIN	0.7449139294	-0.06770999	0.7251692	0.04639623
NH_4^+	0.0004108426	-0.04073561	0.3672286	0.04230855
NO_2^-	0.0186188635	-0.05158923	0.4473117	0.05296705
NO_3^-	**1.1938164317**	0.10643676	**1.1647857**	-0.07219932
Fe	0.0761344935	0.04829419	0.3701818	-0.04770001
Si	**1.0040927560**	0.09568034	0.9730387	-0.06711979
PO_4^{3-}	0.8829044972	0.05801240	0.9078752	-0.03189882
Chlorophyll a	**1.1668409466**	0.12469494	**1.1261470**	-0.09202232
Absolute latitude	**1.4894446409**	0.18268462	**1.4563720**	-0.14187917

Table S3: Partial least square analyses to explore the correlations between diatom relative abundance and biotic factors

	Diatom relative abundance	
	VIP score	*Standard coefficient*
Prochlorococcus	**2.0589894**	-0.236453116
Synechococcus	0.7559680	-0.087359284
Copepoda	**1.4889173**	0.151032888
Centroheliozoa	**1.5517588**	-0.059139034
Choanoflagellida	**1.7055238**	0.195750218
Chrysophyceae	**1.4418286**	-0.007121123
Dictyochophyceae	**2.3466947**	-0.082131732
Nassellaria	**1.4064072**	-0.162014670
Phaeodarea	0.4485323	-0.051816101
Spumellaria	0.9581452	-0.094281817
Rhizaria	0.5690121	-0.055823984

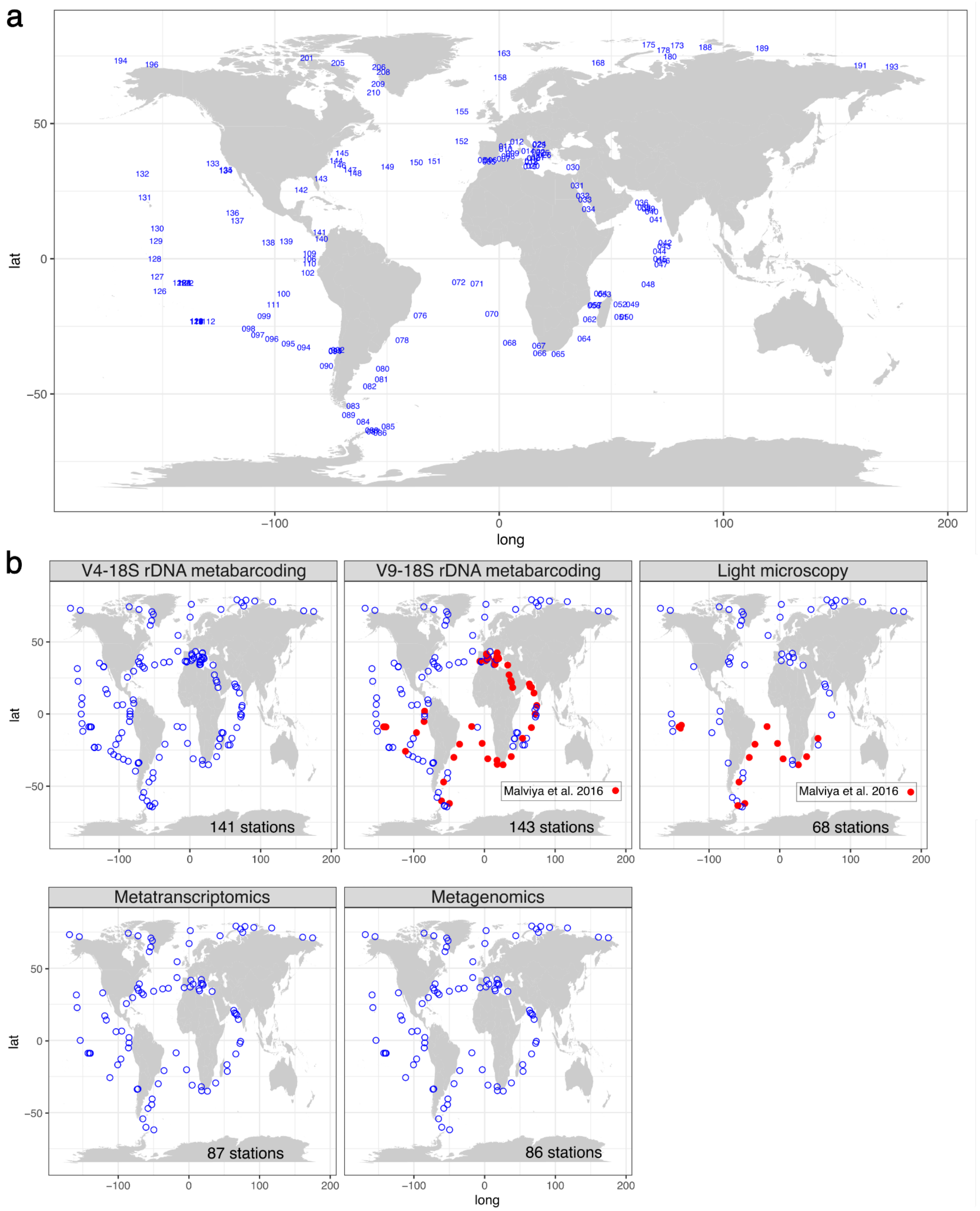

Figure S1: Biogeographical coverage of the *Tara* Oceans datasets used in the current work. a) Sampling station labels. b) Biogeography of the datasets. The total number of stations are indicated. The sampling stations reported by Malviya et al. 2016 *PNAS* 113 (11):E1516-E1525 are marked in red: 43 sampling stations for the V9-based survey and 15 stations for the microscopy survey. Maps were generated with the *borders()* function in *ggplot2*[84].

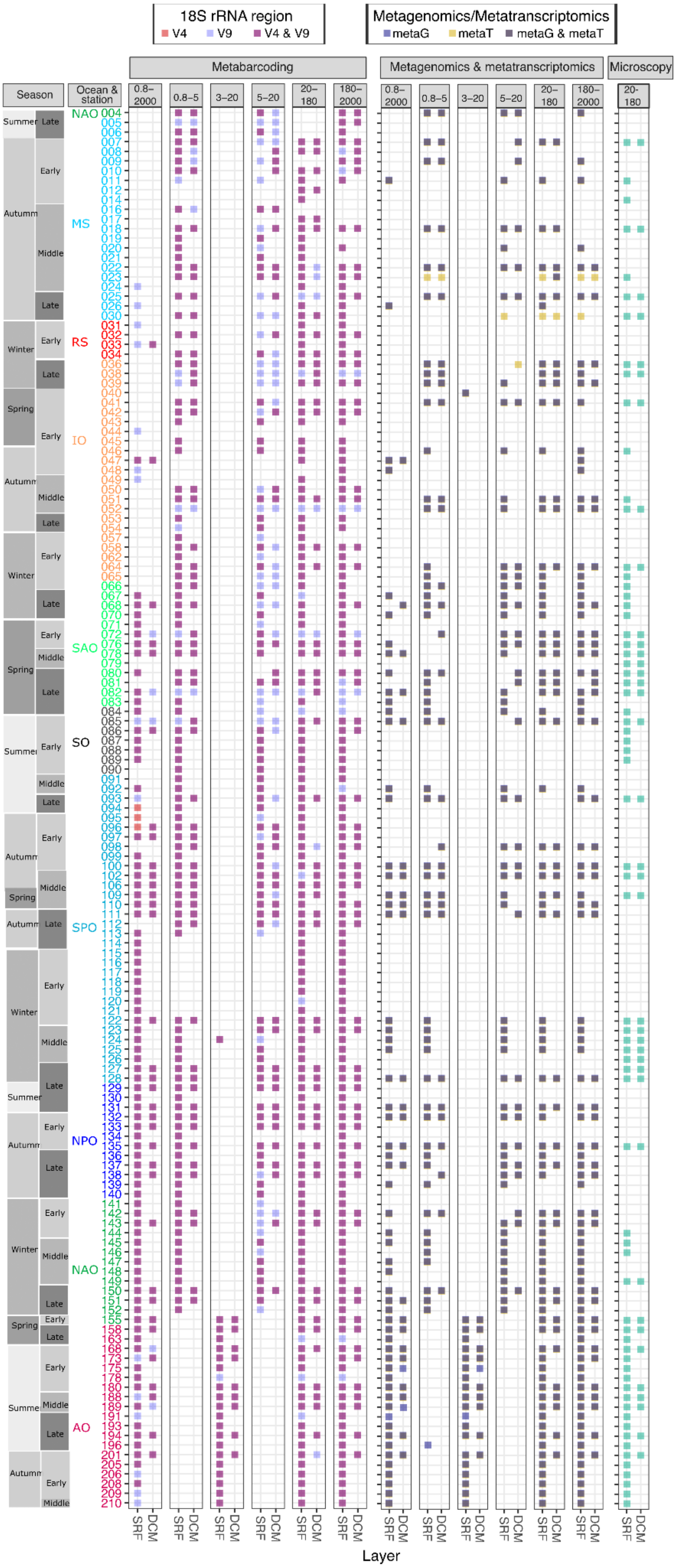

18S rRNA region
V4
V9
V4 & V9
Metagenomics/Metatranscriptomics
metaG
metaT
metaG & metaT
Metabarcoding
Metagenomics & metatranscriptomics
Microscopy
Season
Ocean & station
0.8–2000
0.8–5
3–20
5–20
20–180
180–2000
Summer
Autumn
Winter
Spring
Early
Middle
Late
NAO
MS
RS
IO
SAO
SO
SPO
NPO
AO
SRF
DCM
Layer

Figure S2: Samples and methods used in this study. The current analysis of global diversity and abundance of diatoms was carried out across 145 *Tara* Oceans stations where samples were taken for metabarcoding and/or metagenomics and/or metatranscriptomics and/or optical microscopy. The analyzed samples are indicated as filled boxes, with color according to the method. Two distinct depth layers were sampled: surface (SUR; 5 m) and deep chlorophyll maximum (DCM; 17-180 m). The data from the bottom of the mixed layer was collected when no DCM was observed (stations TARA_123, TARA_124, TARA_125, TARA_152 and TARA_153). Plankton communities were fractionated into four size classes: piconanoplankton (0.8 to 5 μm or 0.8 to 2000 μm), nanoplankton (5 to 20 μm or 3 to 20 μm), microplankton (20 to 180 μm), and mesoplankton (180 to 2000 μm). Season and moment of the season (early, middle, late) are displayed to the left of the panel. Station labels are coloured according to the ocean region: IO, Indian Ocean; MS, Mediterranean Sea; NAO, North Atlantic Ocean; RS, Red Sea; SAO, South Atlantic Ocean; SO, Southern Ocean; SPO, South Pacific Ocean.

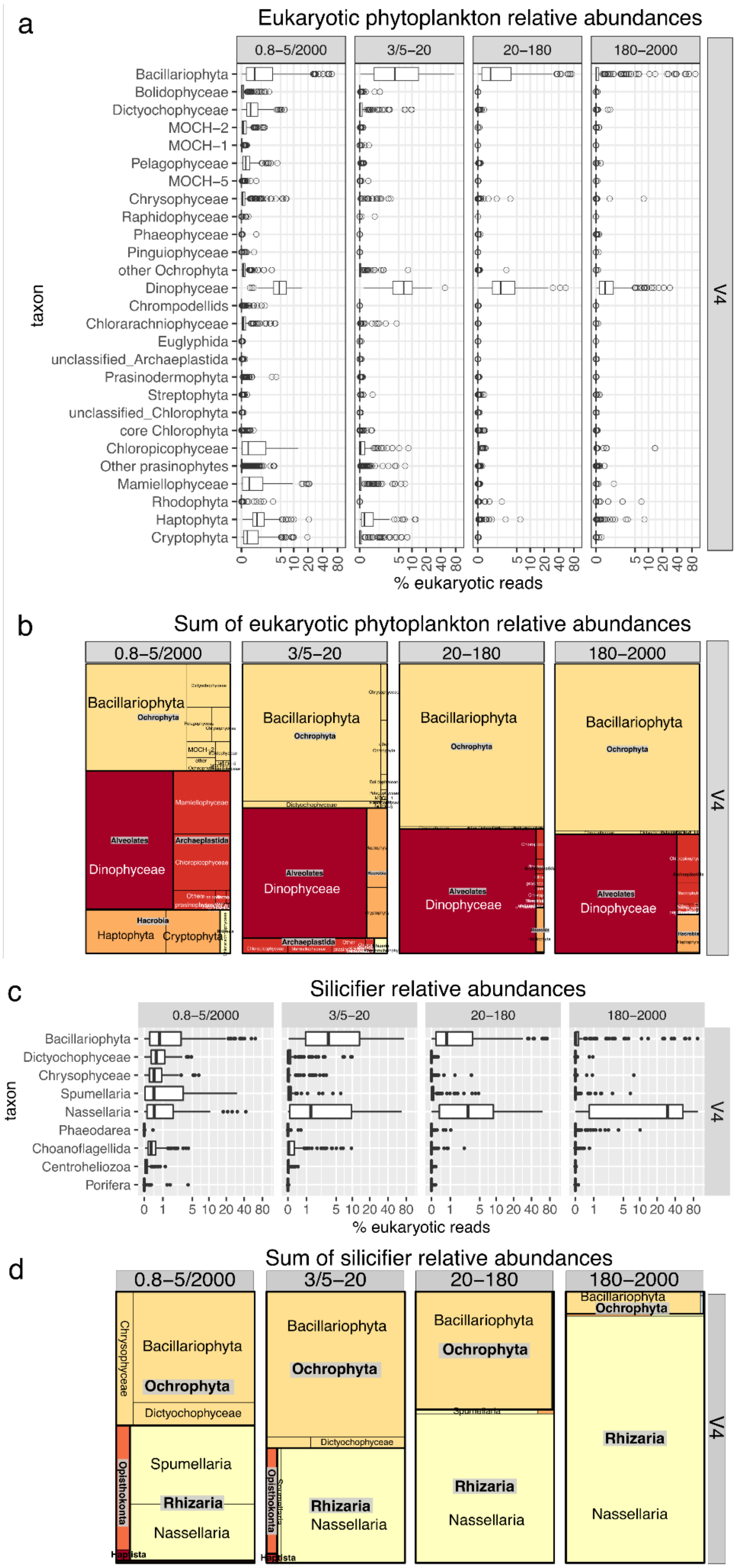
a
Eukaryotic phytoplankton relative abundances
0.8–5/2000
3/5–20
20–180
180–2000
V4
taxon
Bacillariophyta
Bolidophyceae
Dictyochophyceae
MOCH–2
MOCH–1
Pelagophyceae
MOCH–5
Chrysophyceae
Raphidophyceae
Phaeophyceae
Pinguiophyceae
other Ochrophyta
Dinophyceae
Chrompodellids
Chlorarachniophyceae
Euglyphida
unclassified_Archaeplastida
Prasinodermophyta
Streptophyta
unclassified_Chlorophyta
core Chlorophyta
Chloropicophyceae
Other prasinophytes
Mamiellophyceae
Rhodophyta
Haptophyta
Cryptophyta
0
5
10
20
40
80
% eukaryotic reads
b
Sum of eukaryotic phytoplankton relative abundances
0.8–5/2000
3/5–20
20–180
180–2000
V4
Bacillariophyta
Ochrophyta
Alveolates
Dinophyceae
Archaeplastida
Mamiellophyceae
Chloropicophyceae
Hacrobia
Haptophyta
Cryptophyta
Dictyochophyceae
c
Silicifier relative abundances
0.8–5/2000
3/5–20
20–180
180–2000
V4
taxon
Bacillariophyta
Dictyochophyceae
Chrysophyceae
Spumellaria
Nassellaria
Phaeodarea
Choanoflagellida
Centroheliozoa
Porifera
0
1
5
10
20
40
80
% eukaryotic reads
d
Sum of silicifier relative abundances
0.8–5/2000
3/5–20
20–180
180–2000
V4
Chrysophyceae
Bacillariophyta
Ochrophyta
Dictyochophyceae
Opisthokonta
Spumellaria
Rhizaria
Nassellaria
Haptista

Figure S3: Contribution of diatoms to eukaryotic phytoplankton (a-b) and silicifiers (c-d) in surface waters of the global ocean using V4 metabarcoding data obtained from different size-fractions. a-b) Phytoplankton. We focused exclusively on the phytoplankton signal of these data sets, including dinoflagellates and chrysophytes though we acknowledge there are uncertainties in assigning the capacity for photosynthesis in these groups. a) Relative abundances (log scale). Each point is a size-fractionated sample. b) Sum of normalized reads in the overall dataset. c-d) Silicifiers. The equivalent plots for the V9 marker are displayed in Fig 1. Boxplots illustrate the distribution of the dataset, with the box representing the 25–75% interquartile range and the central line indicating the median (50% quantile). Whiskers extend to data points within 1.5 times the interquartile range. The V4 dataset comprises 184 samples for the 0.8–5 µm or 0.8–2000 µm size fractions, 127 for the 3–20 µm or 5–20 µm fractions, 175 for the 20–180 µm fraction, and 185 for the 180–2000 µm fraction.

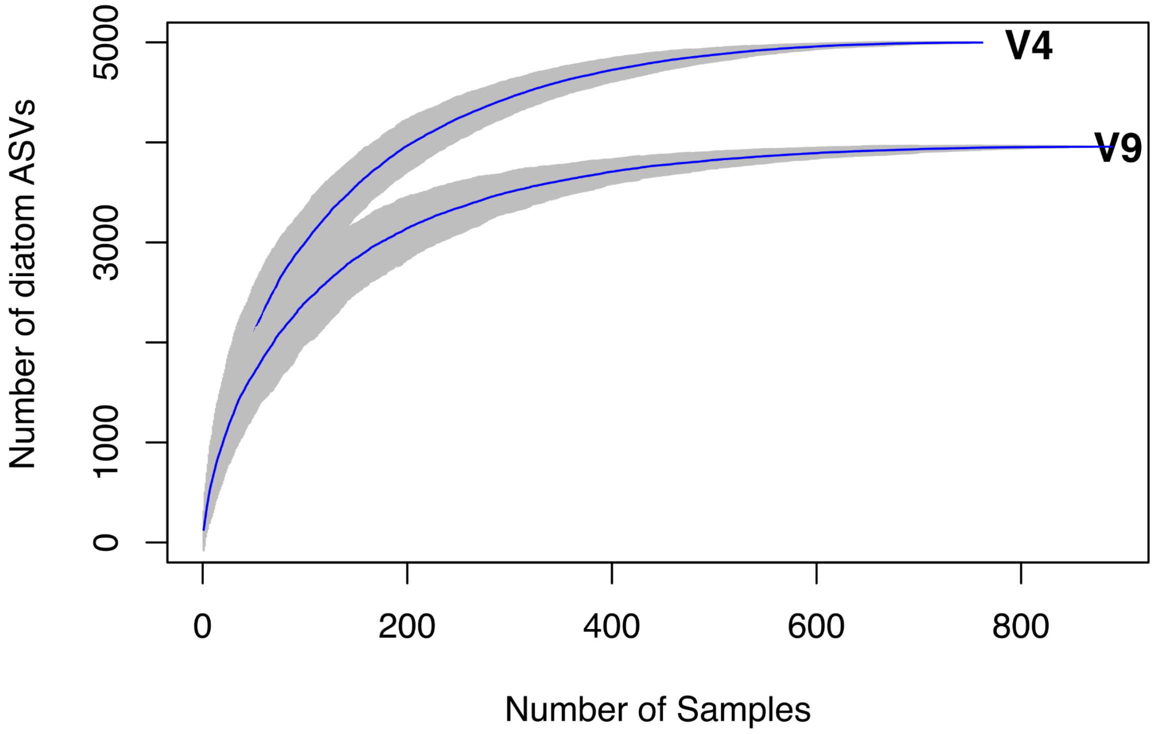

Figure S4: Accumulation curves for V4 and V9 18S rRNA gene metabarcoding assigned to diatoms.

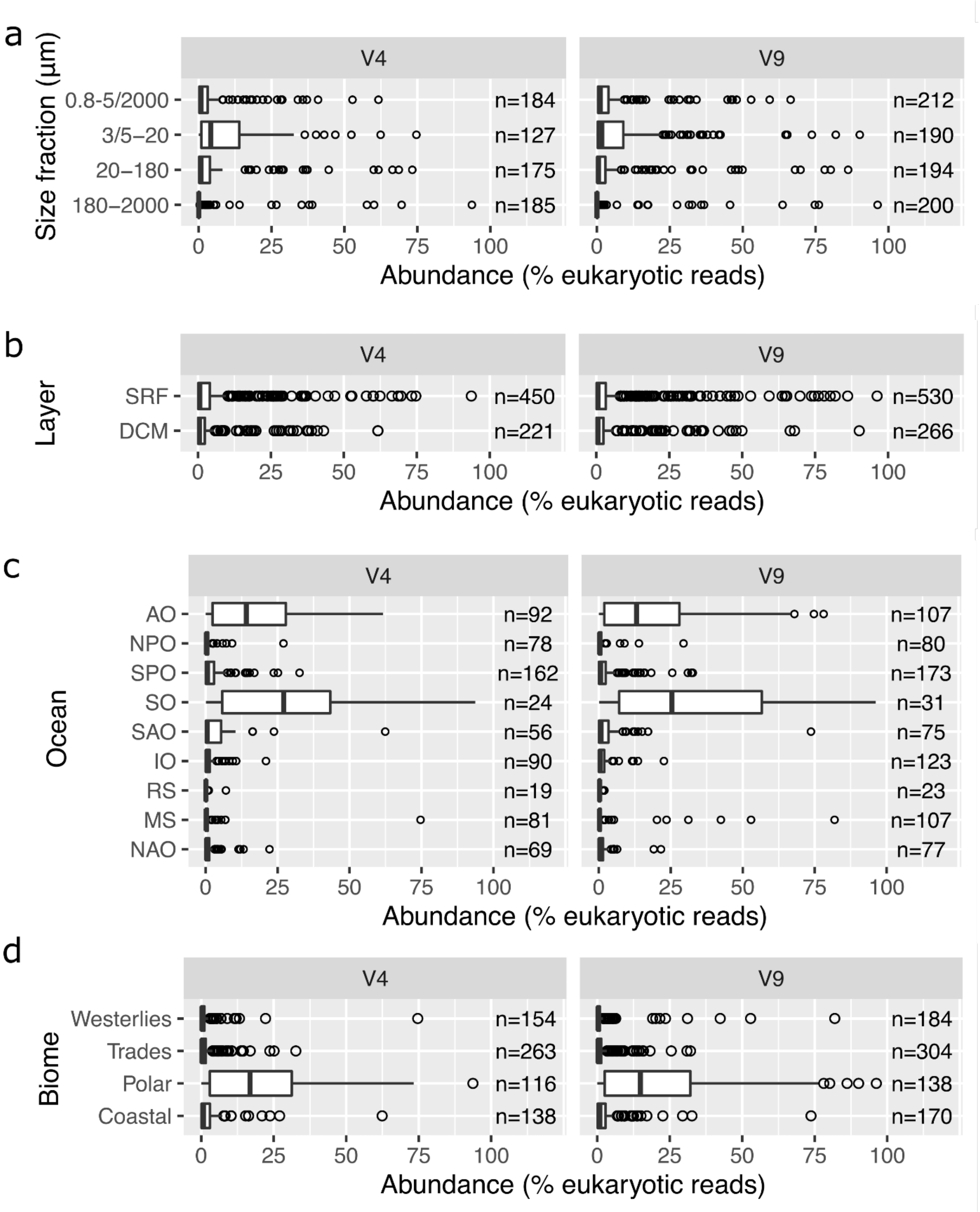

Figure S5: Distribution of diatom relative abundances in epipelagic seawater samples from the V9 and V4 metabarcoding data across size fractions, water layers, and ocean basins. a) Size fractions. b) Water layers (SRF, surface; DCM, deep chlorophyll maximum). c) Ocean basins (MS, Mediterranean Sea, IO, Indian Ocean, SAO, South Atlantic Ocean, SO, Southern Ocean, SPO, South Pacific Ocean, NPO, North Pacific Ocean, NAO, North Atlantic Ocean, AO, Arctic Ocean). d) Biomes.

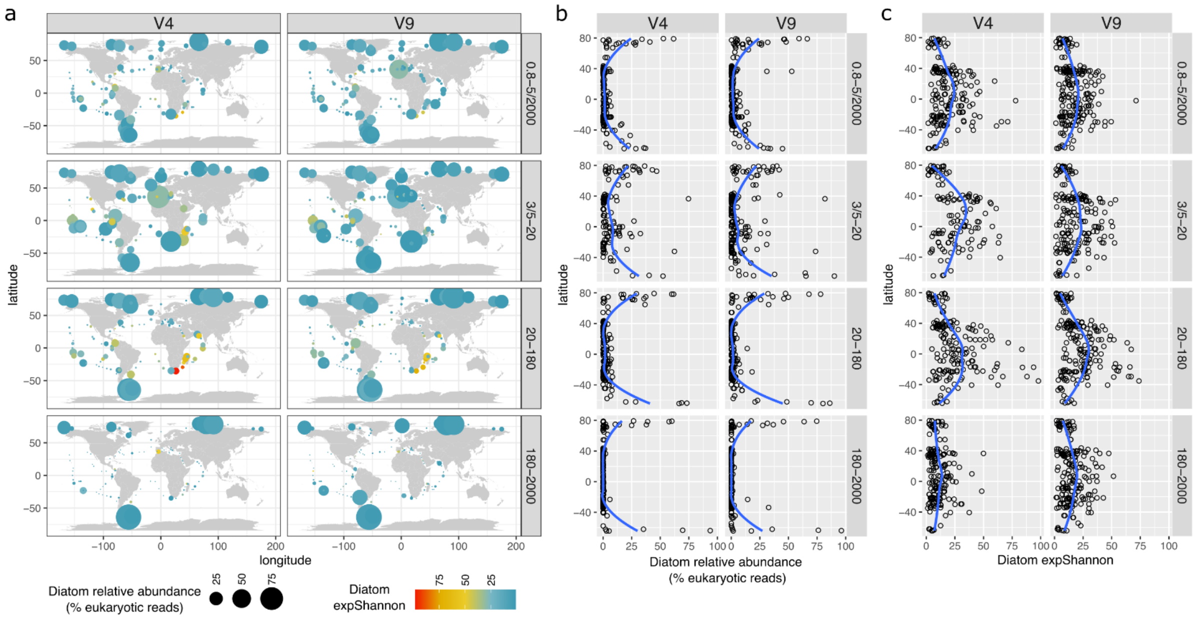

Figure S6: Comparison between V4 and V9 metabarcoding for the global macroecological patterns of diatom relative abundance and diversity in surface waters using data obtained from different size-fractions. a) Distribution maps. b) Latitudinal gradient for relative abundance. c) Latitudinal gradient for the exponentiated Shannon Diversity Index. The blue lines correspond to Loess smoothings. Maps were generated with the *borders()* function in *ggplot2*[84].

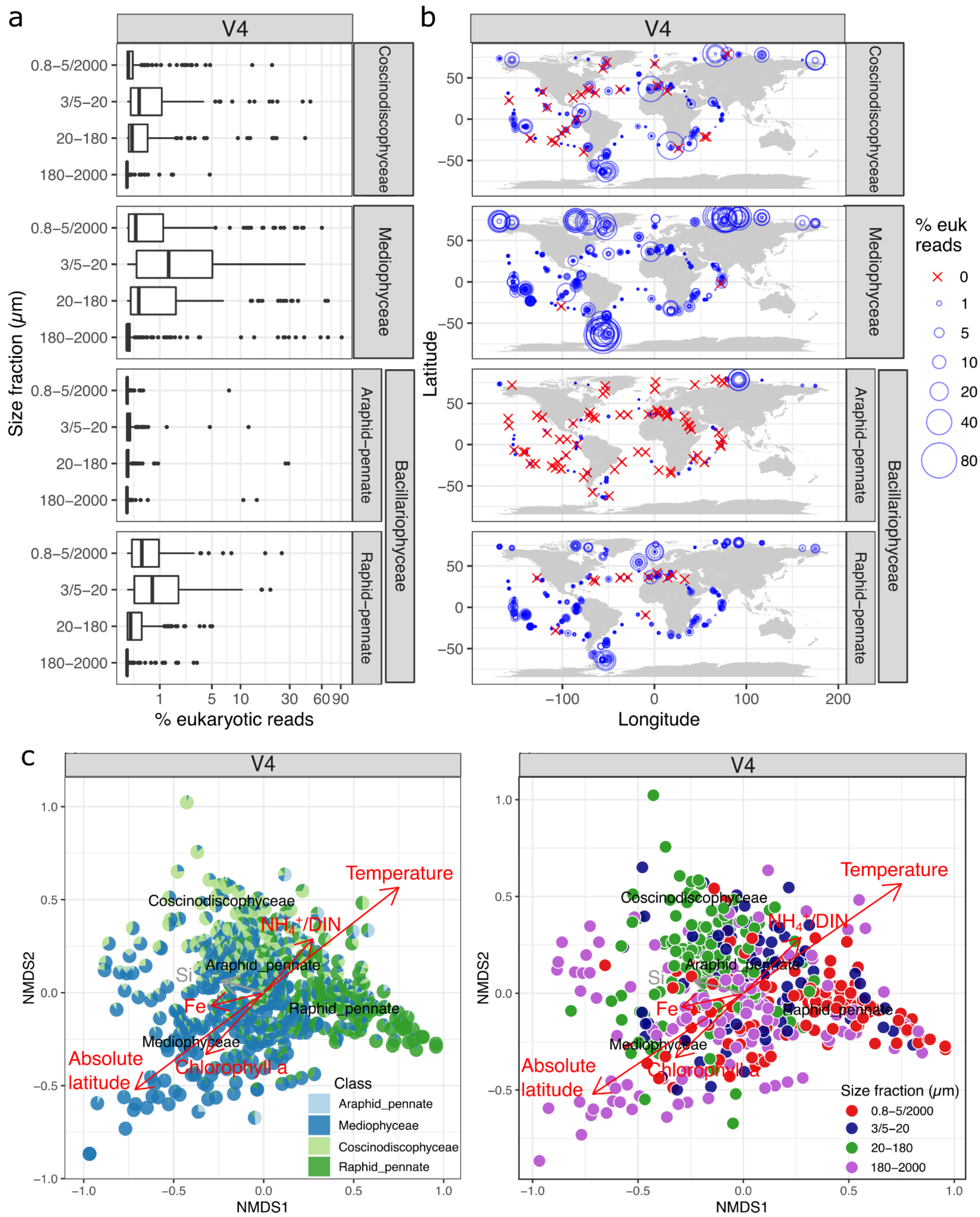

Figure S7: Abundance patterns of diatom classes based on the V4 marker. a) Relative abundances (log scale). Each point is a size-fractionated sample. b) Biogeography. c) NMDS analysis of stations according to Bray–Curtis distance. Fitted statistically significant physico-chemical parameters are displayed in black lines when adjusted P value < 0.05 (temperature, iron, chlorophyll a concentration, absolute latitude, ratio of ammonium to total dissolved inorganic nitrogen) and in gray lines when between 0.1 and 0.05 (silicon). Each pie chart (left) or each circle (right) is a size-fractionated sample. NMDS stress value: 0.1197578. The equivalent figure for V9 is displayed in **Figure 4**. Boxplots illustrate the distribution of the dataset, with the box representing the 25–75% interquartile range and the central line indicating the median (50% quantile). Whiskers extend to data points within 1.5 times the interquartile range. The V4 dataset comprises 184 samples for the 0.8–5 μm or 0.8–2000 μm size fractions, 127 for the 3–20 μm or 5–20 μm fractions, 175 for the 20–180 μm fraction, and 185 for the 180–2000 μm fraction. Maps were generated with the *borders()* function in *ggplot2*[84].

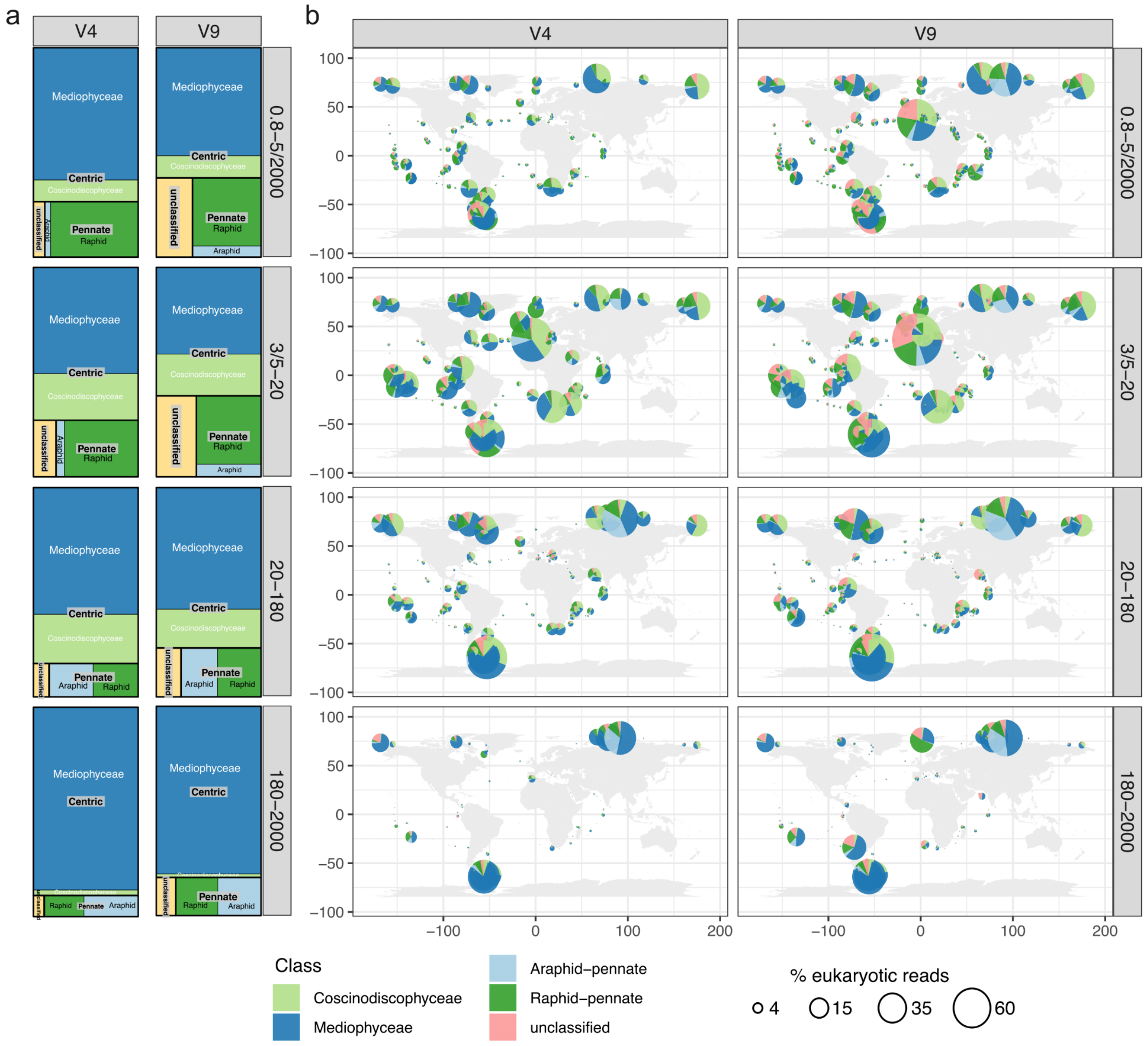

Figure S8: Relative contribution of the different diatom classes. a) Sum of normalized reads for V4 and V9 markers in the overall dataset. b) Biogeography. The maps separated by diatom classes are shown in Fig S9. Maps were generated with the *borders()* function in *ggplot2*[84].

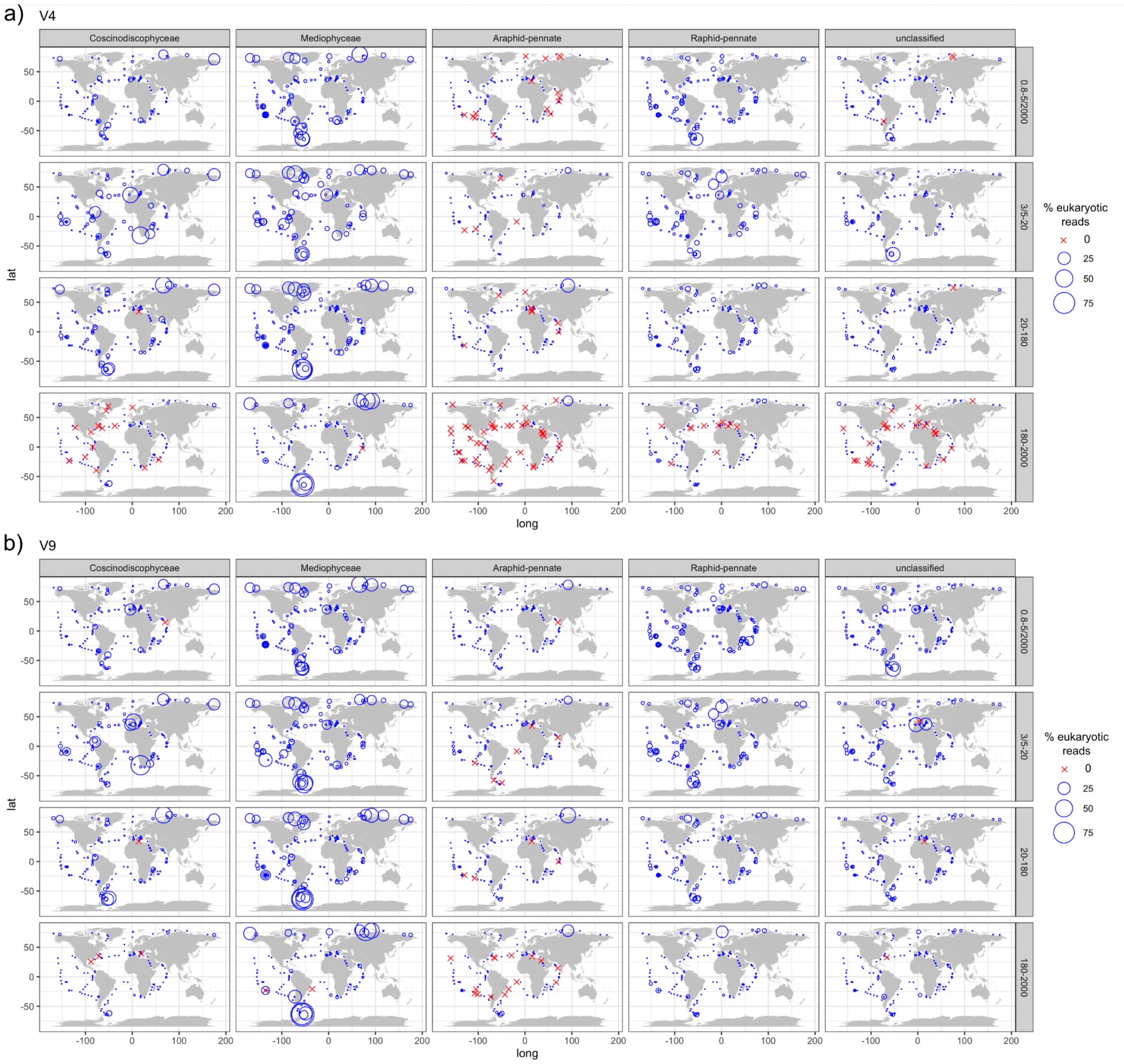

Figure S9: Biogeography of diatom classes in surface waters using V4 (a) and V9 (b) metabarcoding data obtained from different size-fractionated samples. The bubble sizes vary according to the percentage of diatoms among eukaryotes. Maps were generated with the *borders()* function in *ggplot2*[84].

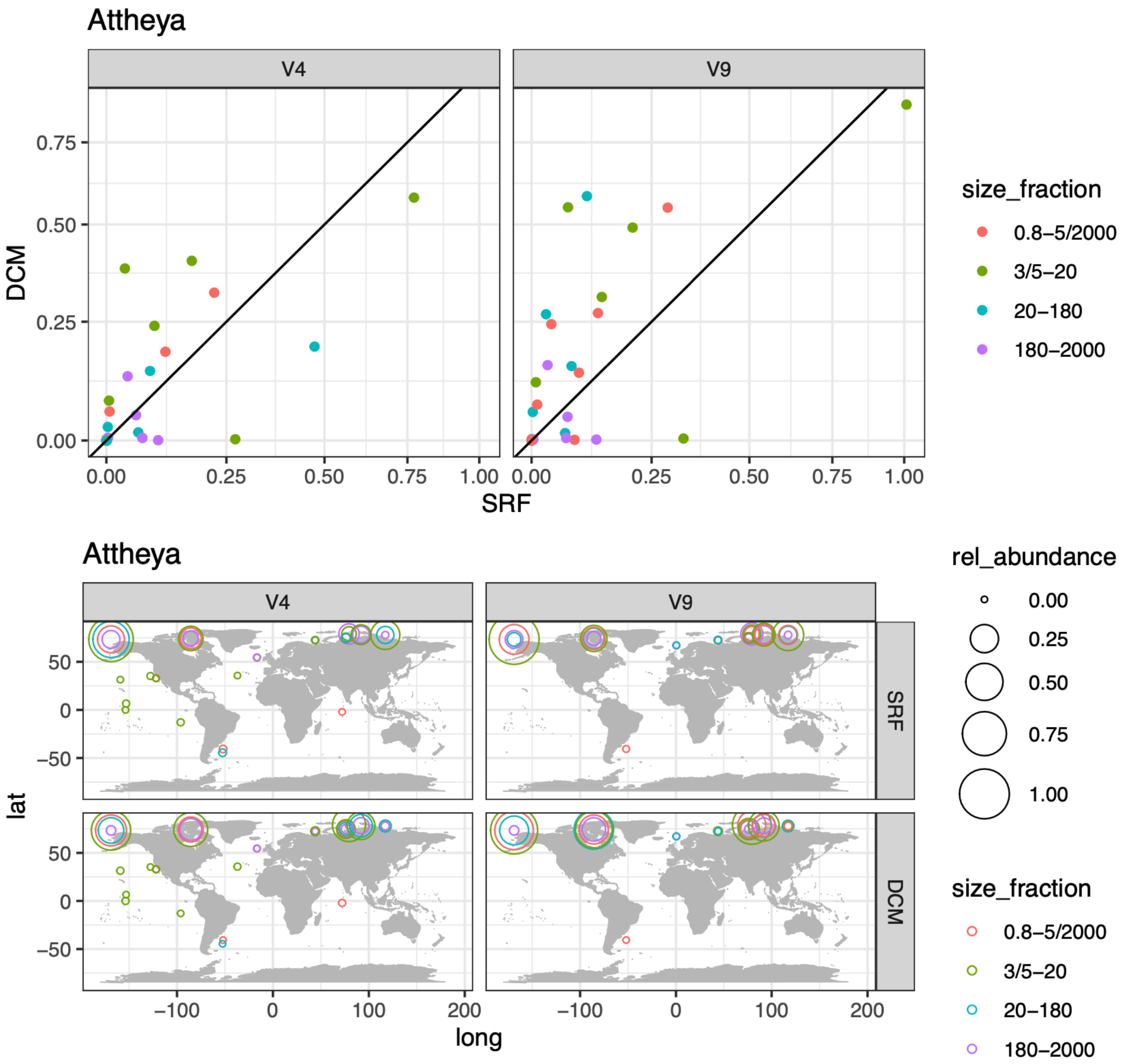

Figure S10: Environmental distribution of the *Attheya* genus. a) Depth distribution. The scatter plots compare the relative abundances between surface (5 m) and deep chlorophyll maximum (DCM; 17–180 m). Axes are in the same scale and the diagonal line corresponds to a 1:1 slope. b) Biogeography. The bubble sizes vary according to the percentage of diatoms among eukaryotes. The data corresponds to V4 (left panel) and V9 (right panel) metabarcoding data obtained from different size-fractionated samples (indicated in colors). Maps were generated with the *borders()* function in *ggplot2*[84].

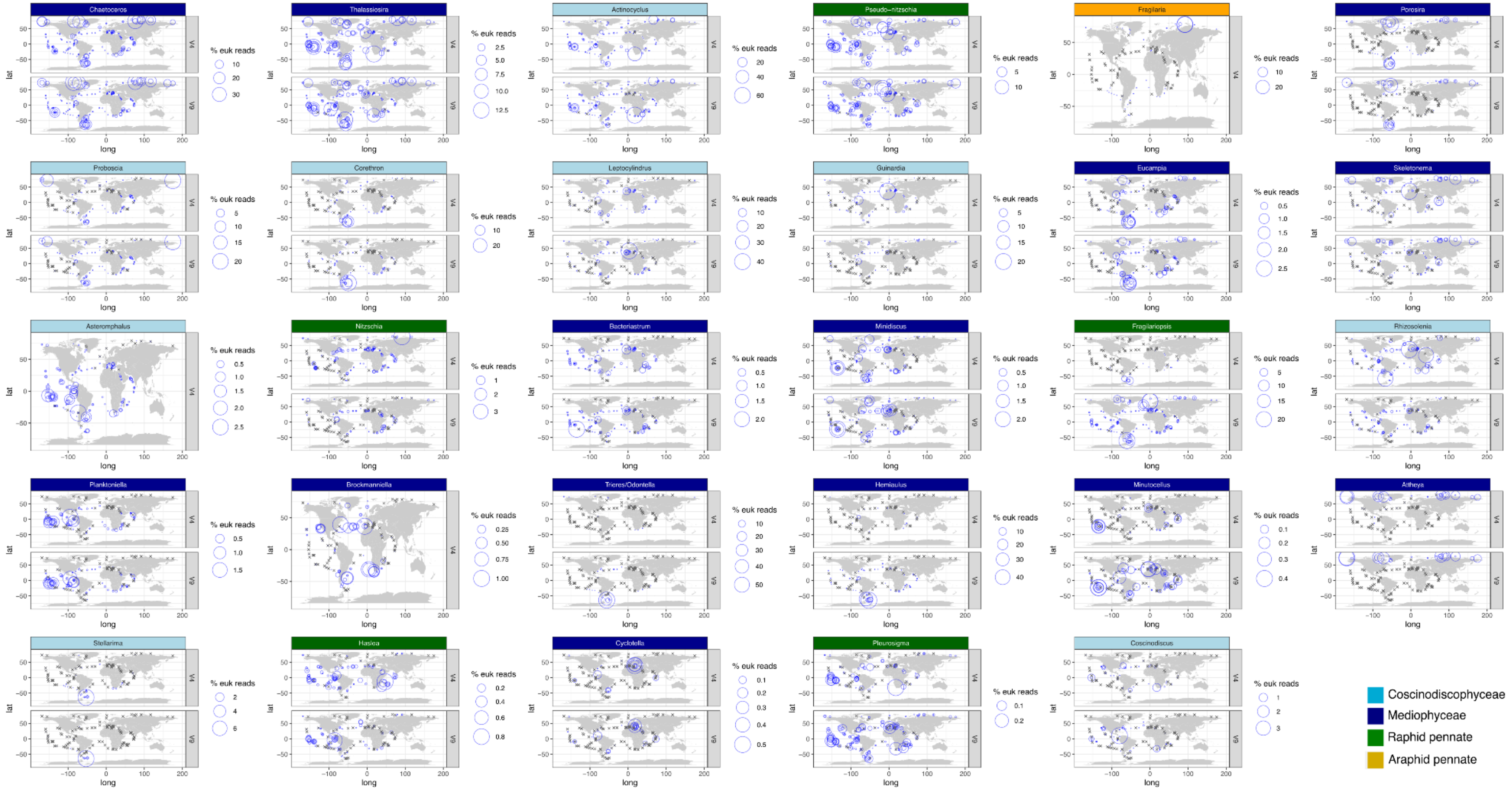
Chaetoceros
Thalassiosira
Actinocyclus
Pseudo−nitzschia
Fragilaria
Porosira
Proboscia
Corethron
Leptocylindrus
Guinardia
Eucampia
Skeletonema
Asteromphalus
Nitzschia
Bacteriastrum
Minidiscus
Fragilariopsis
Rhizosolenia
Planktoniella
Brockmanniella
Triceres/Odontella
Hemiaulus
Minutocellus
Attheya
Stellarima
Haslea
Cyclotella
Pleurosigma
Coscinodiscus
% euk reads
lat
long
V4
V9
Coscinodiscophyceae
Mediophyceae
Raphid pennate
Araphid pennate

Figure S11: Global distribution of the 20 most abundant genera in the V9 and V4 datasets. All genera in the list of Fig 6a are displayed. This figure is analogous to Figure 7, but differentiates by marker region. Bubble areas represent the percentage of reads for each genus among eukaryotic reads at each station location, with crosses indicating absence of detection. The maps depict surface samples from the size fraction where the genus was most prevalent: 20-180 µm for *Chaetoceros*, *Fragilaria*, *Porosira*, *Proboscia*, *Corethron*, Eucampia, Asteromphalus, Planktoniella and *Trieres*/*Odontella, and Stellarima*; 3/5-20 µm for *Thalassiosira*, *Actinocyclus*, *Pseudo-nitzschia*, *Guinardia*, *Leptocylindrus*, *Fragilariopsis*, Skeletonema, Bacteriastrum, Rhizosolenia, Attheya, Haslea, Pleurosigma, Coscinodiscus, and *Hemiaulus*; 0.8-5/2000 µm for *Nitzschia, Minidiscus, Brockmanniella, Minutocellus*, and *Cyclotella*. The maps including all size fractions and covering the top 50 most abundant genera are available in Supplementary File 1. Maps were generated with the *borders()* function in *ggplot2*[84].

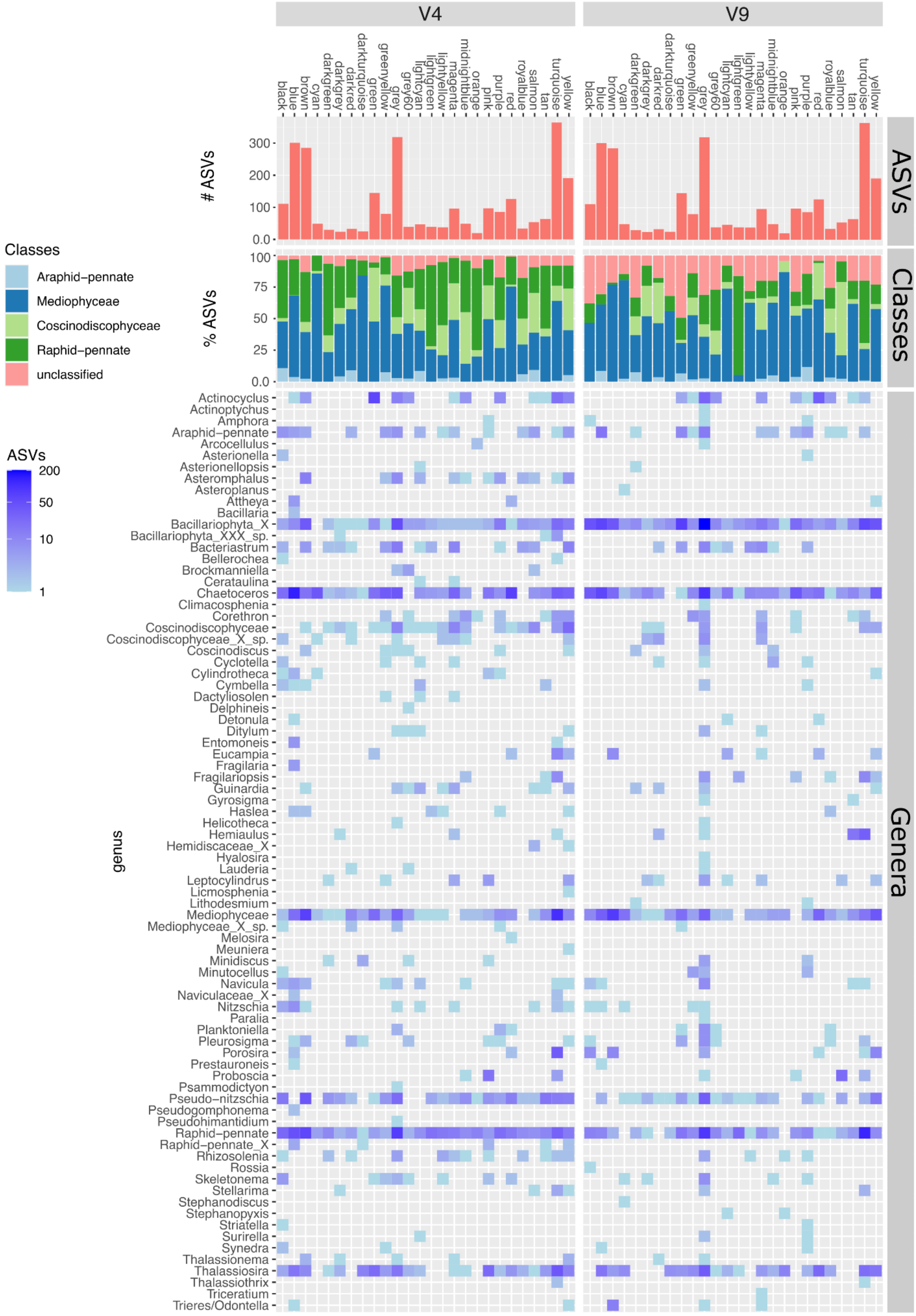

Figure S12: Taxonomic composition of the diatom ASV modules obtained by WGCNA.

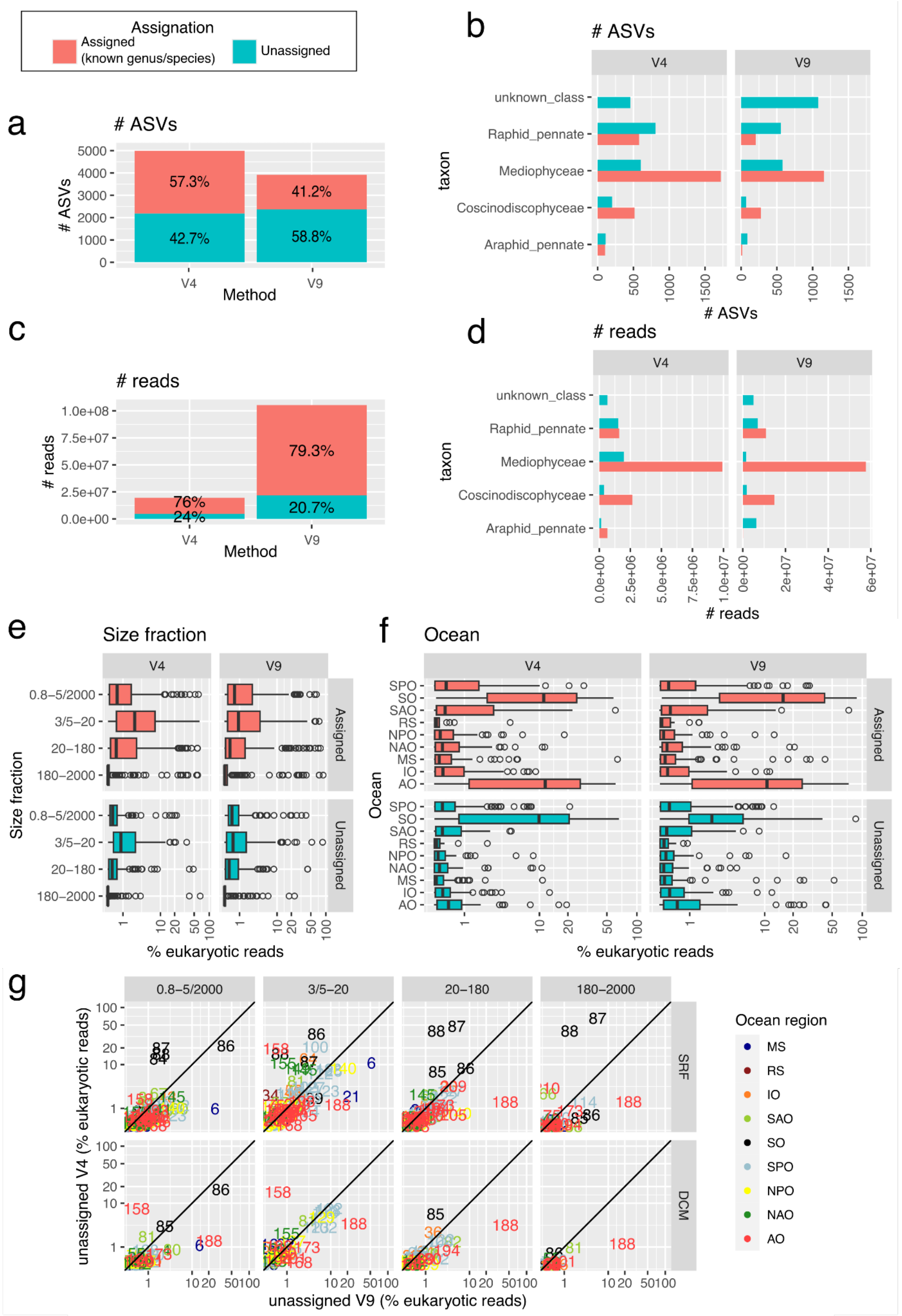

Assignation
Assigned (known genus/species)
Unassigned
a
ASVs
57.3%
42.7%
41.2%
58.8%
Method
b
ASVs
V4
V9
taxon
unknown_class
Raphid_pennate
Mediophyceae
Coscinodiscophyceae
Araphid_pennate
c
reads
76%
24%
79.3%
20.7%
Method
d
reads
e
Size fraction
0.8–5/2000
3/5–20
20–180
180–2000
Assigned
Unassigned
% eukaryotic reads
f
Ocean
SPO
SO
SAO
RS
NPO
NAO
MS
IO
AO
% eukaryotic reads
g
0.8–5/2000
3/5–20
20–180
180–2000
SRF
DCM
unassigned V4 (% eukaryotic reads)
unassigned V9 (% eukaryotic reads)
Ocean region
MS
RS
IO
SAO
SO
SPO
NPO
NAO
AO

Figure S13: Unassigned ASVs in the *Tara* Oceans V4 and V9 metabarcoding datasets from diatoms. The ASVs considered to be unassigned were those that could not be unambiguously assigned to any diatom genus/species but could be classified only as araphid or raphid pennate, Mediophyceae, Coscinodiscophyceae, or unassigned diatom. a-b) Total number of assigned (red) and unassigned (blue) ASVs, and their class-level distribution. c-d) Total number of assigned (red) and unassigned (blue) reads, and their class-level distribution. e) Read abundances of assigned and unassigned ASVs per size class. f) Read abundances of assigned and unassigned ASVs by ocean region. g) Comparison of the read abundance of unassigned ASVs in those samples that are represented by both V4 and V9 metabarcoding. Axes are in the same logarithmic scale and the diagonal line corresponds to a 1:1 slope. Station labels are indicated, with the color according to the ocean region: MS Mediterranean Sea, IO Indian Ocean, SAO South Atlantic Ocean, SO Southern Ocean, SPO South Pacific Ocean, NPO North Pacific Ocean, NAO North Atlantic Ocean, AO Arctic Ocean. Each point in e-g panels corresponds to a size-fractionated sample.

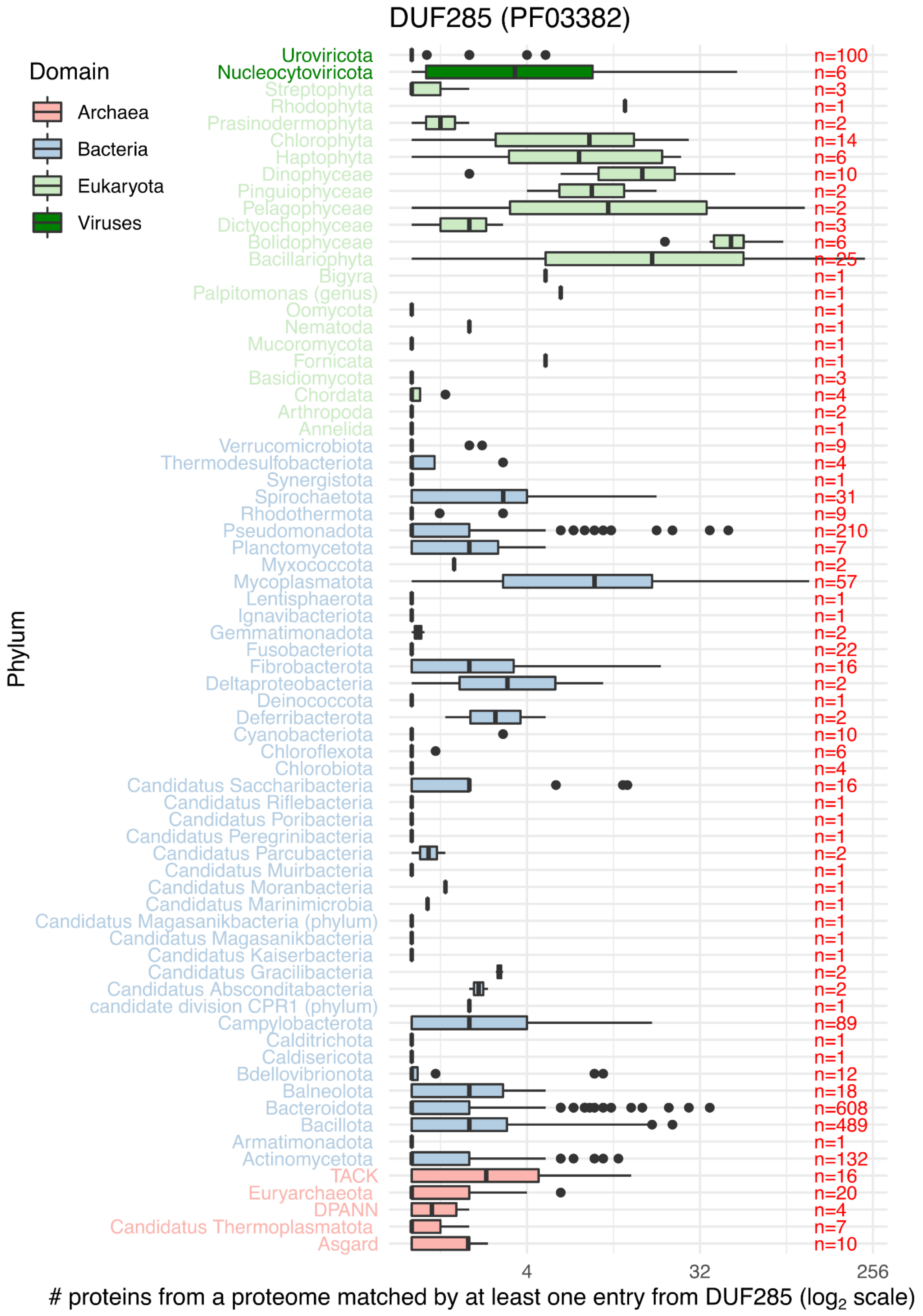

Figure S14: Phylogenetic distribution of DUF285 (PF03382) in reference proteomes from UniprotKB database (https://www.uniprot.org/uniprotkb). The number of analysed species is displayed in red. The x axis corresponds to the number of unique DUF285 sequences per proteome (log_2 scale)

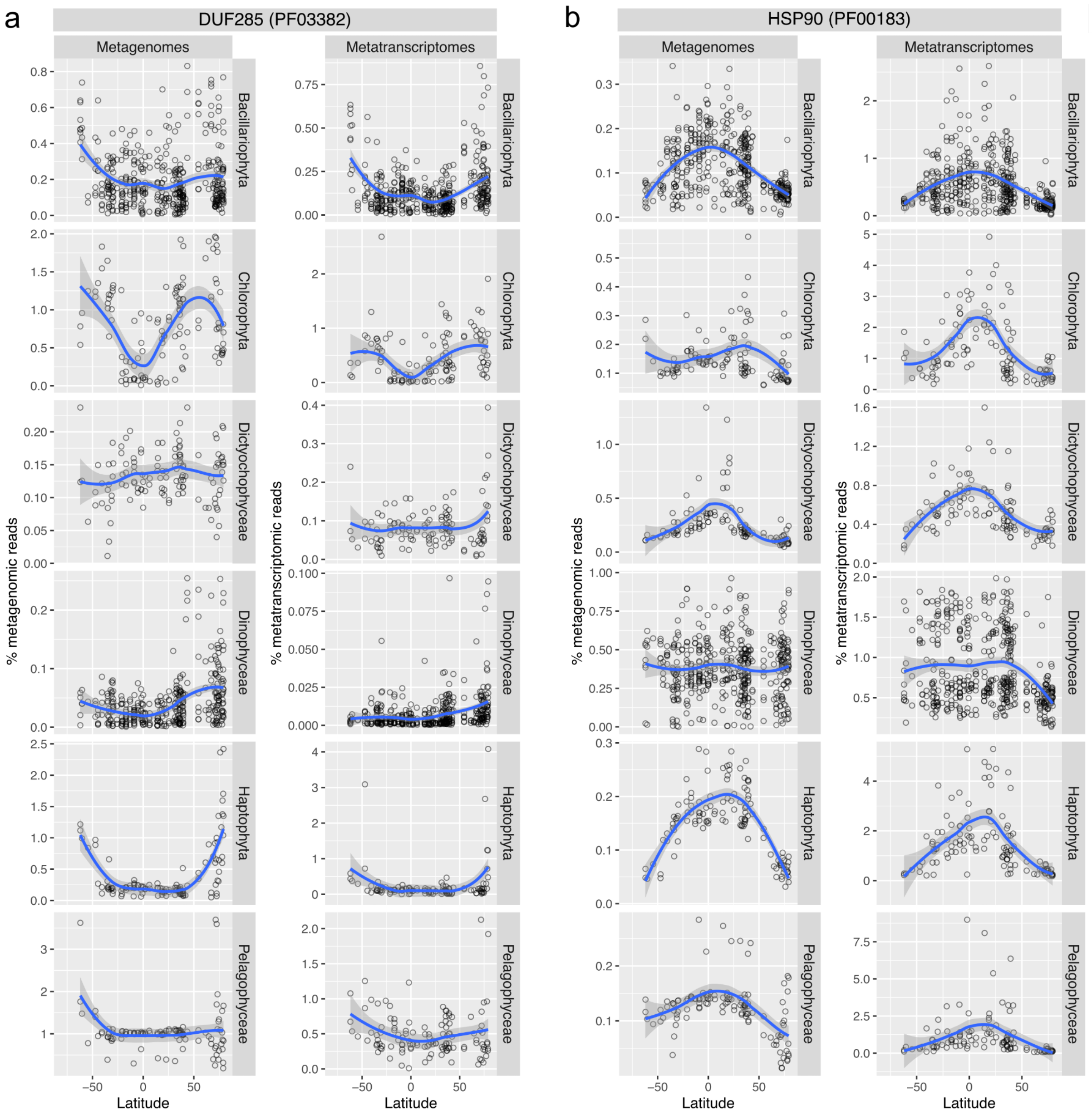

Figure S15: Latitudinal abundance gradient for genes and transcripts coding for Domain of Unknown Function 285 (DUF285) and Heat Shock Protein 90 (HSP90) in diatoms and other eukaryotic phytoplankton. a) DUF285 (PF03382). b) HSP90 (PF00183). The scatter plots correspond to samples from the size fraction where the taxon was most prevalent: 0.8-5/2000, 3/5-20, 20-180, and 180-2000 µm for diatoms and dinoflagellates, and 0.8-5/2000 µm for the rest. Y axis corresponds to the proportion of metagenomic or metatranscriptomic reads for the given function among the total metagenomic or metatranscriptomic reads from the corresponding taxon. Note that we were not able to discard heterotrophic species from dinoflagellates due to the small number of reference gene catalogs for this group.

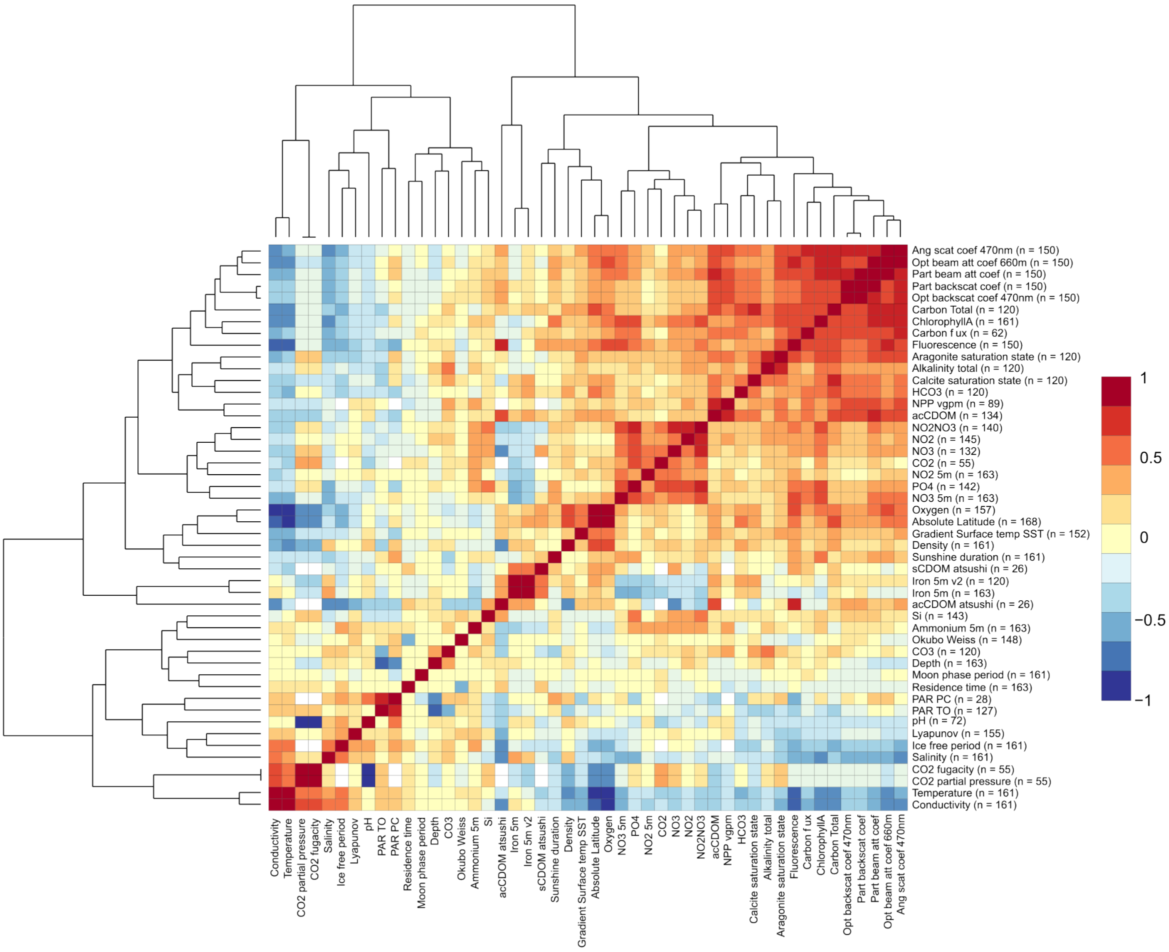

Figure S16: Correlation matrix for the environmental variables analyzed in the current work. Spearman rho correlation values are displayed. Sample size is indicated for each variable.
