# Supplementary material for: Patterns and drivers of diatom diversity and abundance in the global ocean
Source: Nat Commun. 2025 Apr 11;16:3452. doi: 10.1038/s41467-025-58027-7 (PMC11992049; doi:10.1038/s41467-025-58027-7)
Supplement: Supplementary file 2 — Description of Additional Supplementary Files [file 41467_2025_58027_MOESM2_ESM.pdf]

Description of Additional Supplementary Files

Supplementary Data 1: Biogeography of the diatom genera detected in V4 and V9 datasets. Bubble areas are scaled to the % reads for the genus among eukaryotic reads at each station location, whereas crosses indicate absence of detection. Colors indicate the exponentiated Shannon Diversity Index. Note that some genera are only detected in V4 or V9 dataset.

Maps were generated with the *borders()* function in *ggplot2*. [Wickham, H. Data Analysis. in *Use R!* 189–201 (Springer International Publishing, Cham, 2016).]
