# Supplementary material for: Patterns and drivers of diatom diversity and abundance in the global ocean
Source: Nat Commun. 2025 Apr 11;16:3452. doi: 10.1038/s41467-025-58027-7 (PMC11992049; doi:10.1038/s41467-025-58027-7)
Supplement: Supplementary file 3 — Supplementary Data 1 [file 41467_2025_58027_MOESM3_ESM.pdf]

Supplementary Data 1
Patterns and drivers of diatom diversity and abundance in the global ocean
Pierella Karlusich, et al.

Global distribution of the diatom genera detected in V4 and V9 datasets. Bubble sizes are proportional to the % of reads for each genus relative to eukaryotic reads at each sampling station, whereas crosses indicate absence of detection. Colors indicate the exponentiated Shannon Diversity Index. Note that some genera were only detected in either the V4 or V9 dataset. Maps were generated with *borders()* function in *ggplot2*[84].

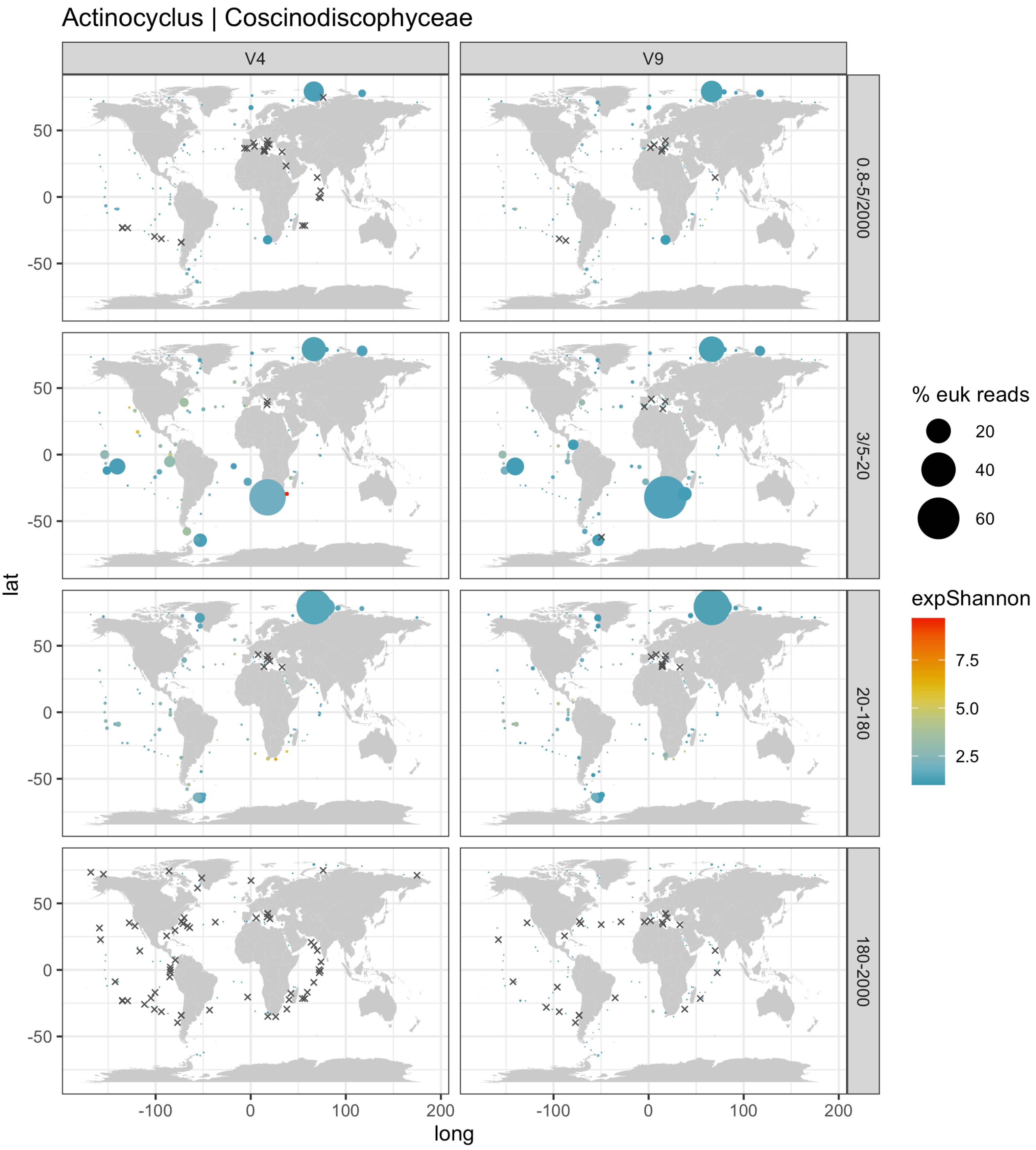

Actinocyclus | Coscinodiscophyceae
V4
V9
0.8-5/2000
3/5-20
20-180
180-2000
lat
long
50
0
-50
-100
100
200
% euk reads
20
40
60
expShannon
7.5
5.0
2.5

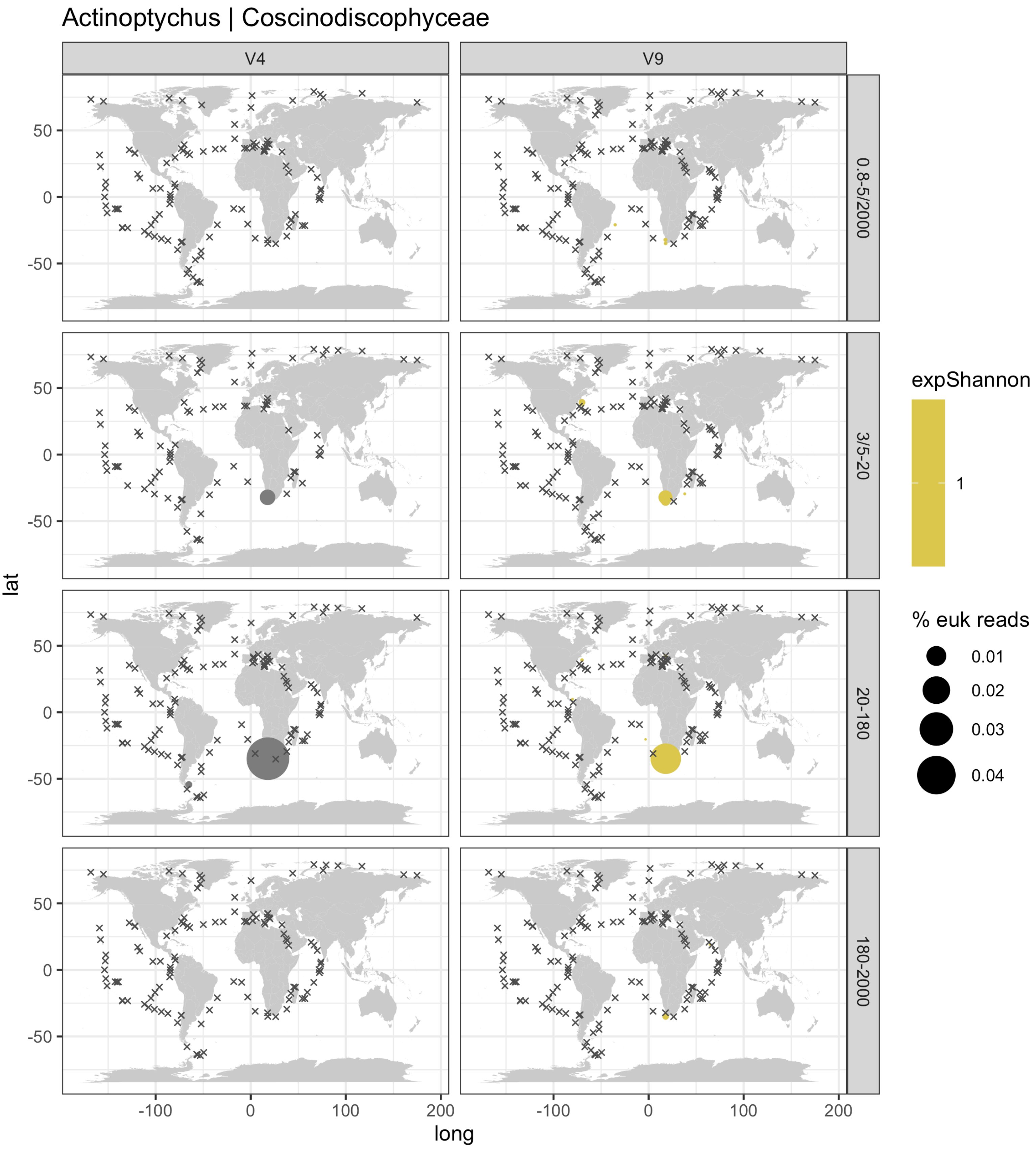
Actinoptychus | Coscinodiscophyceae
V4
V9
0.8-5/2000
3/5-20
20-180
180-2000
lat
long
50
0
-50
-100
100
200
expShannon
1
% euk reads
0.01
0.02
0.03
0.04

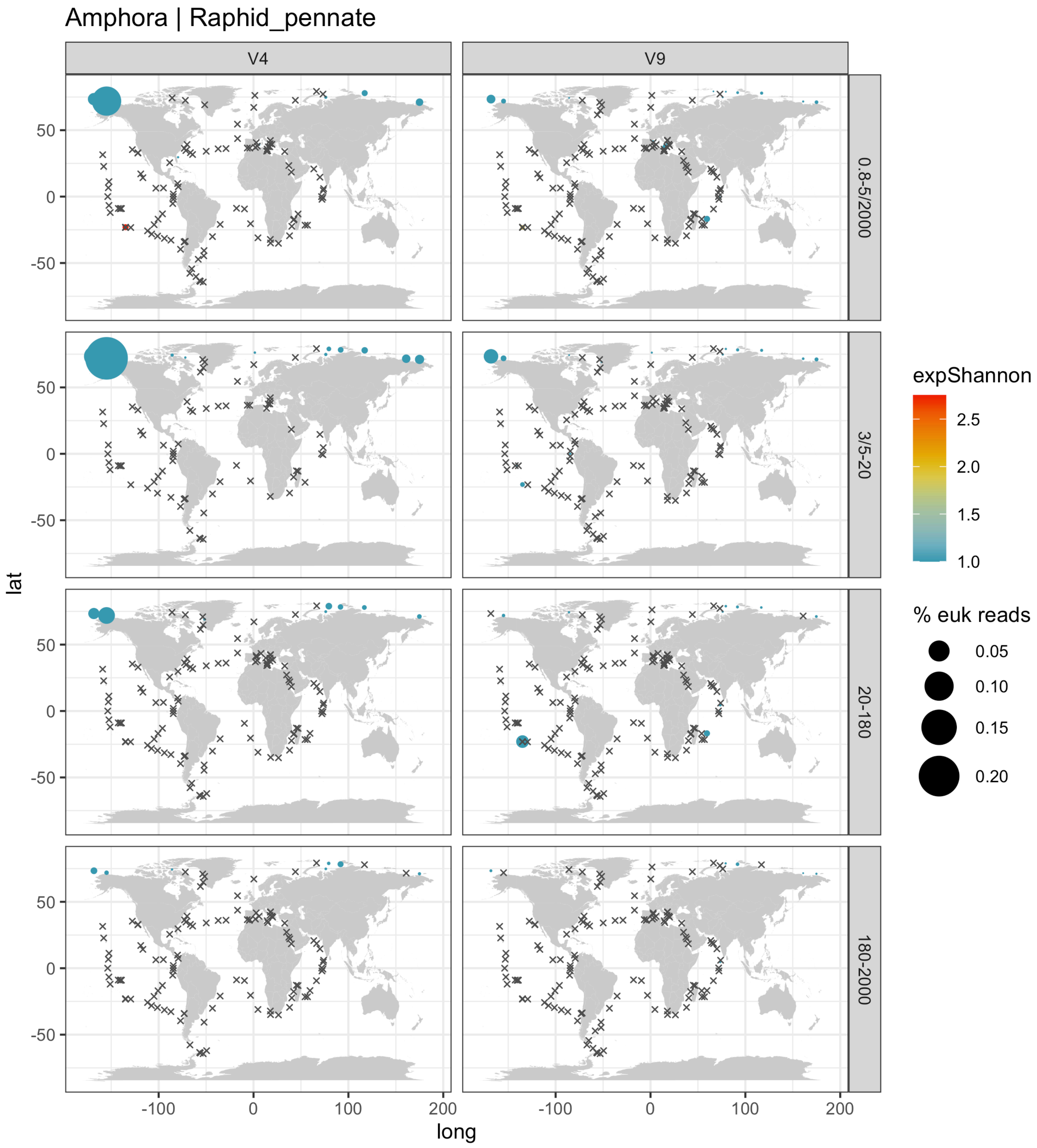

Amphora | Raphid_pennate
V4
V9
0.8-5/2000
3/5-20
20-180
180-2000
lat
long
50
0
-50
-100
0
100
200
expShannon
2.5
2.0
1.5
1.0
% euk reads
0.05
0.10
0.15
0.20

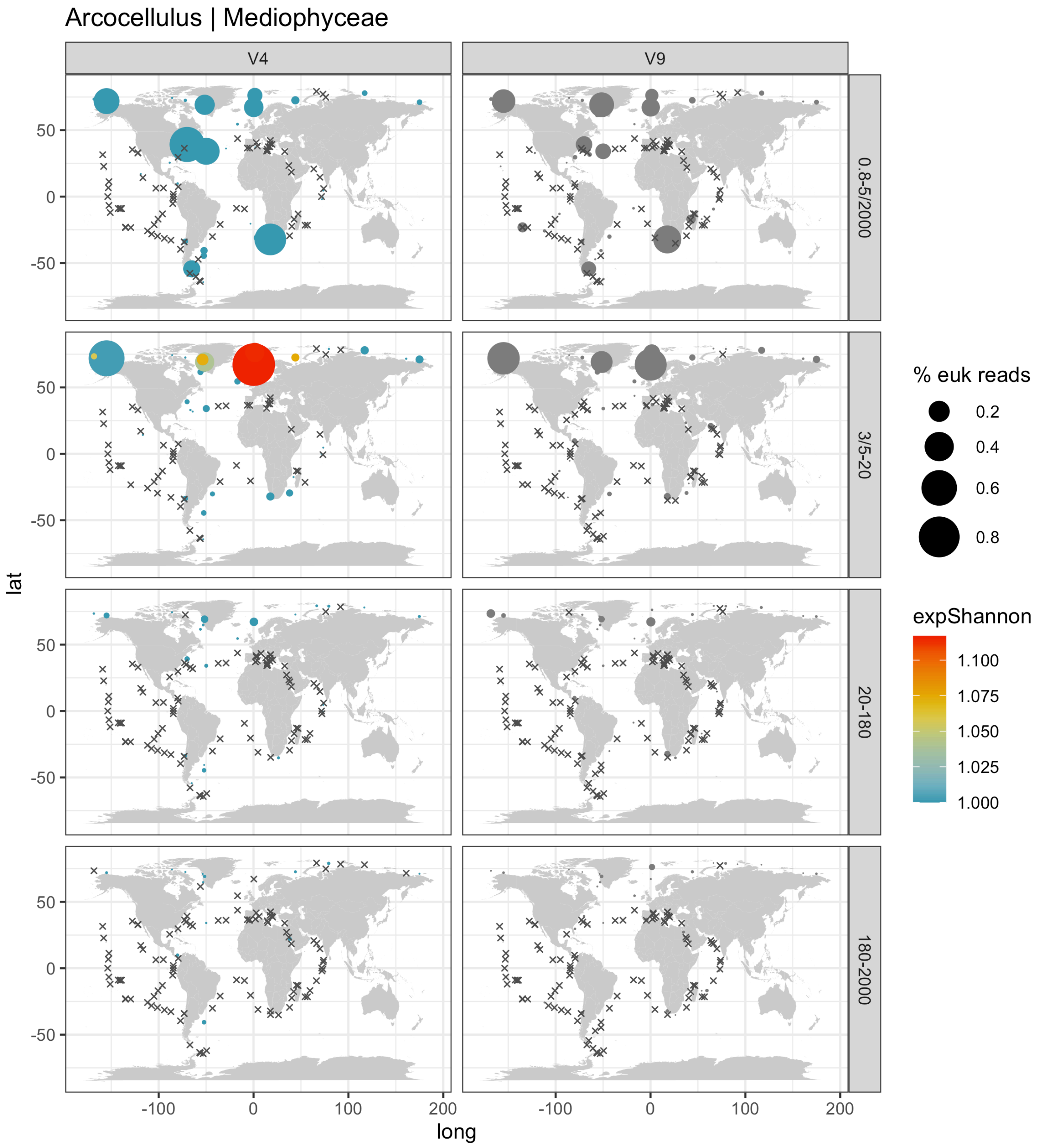
Arcocellulus | Mediophyceae
V4
V9
0.8-5/2000
3/5-20
20-180
180-2000
lat
long
50
0
-50
-100
0
100
200
% euk reads
0.2
0.4
0.6
0.8
expShannon
1.100
1.075
1.050
1.025
1.000

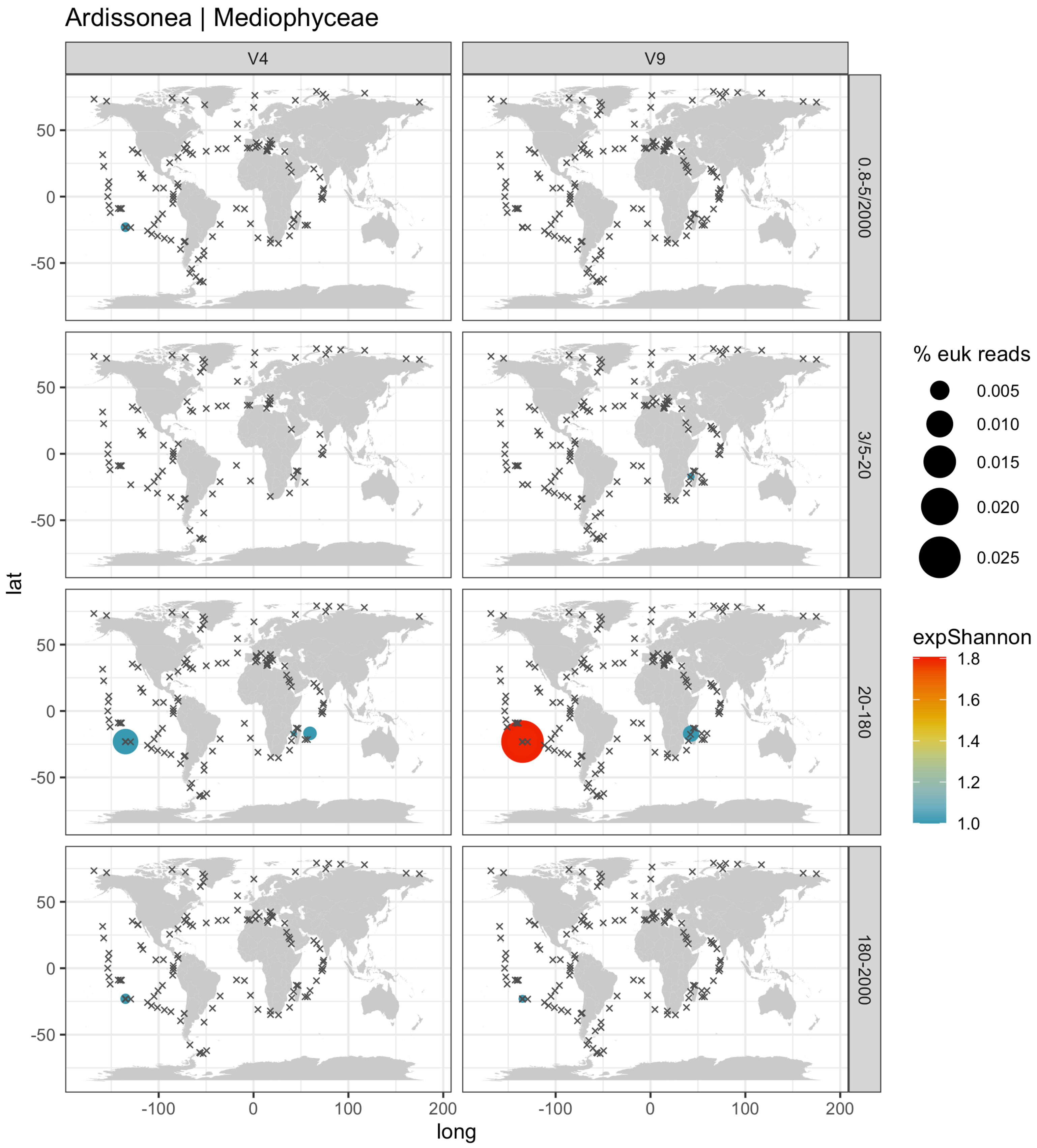

Ardissonea | Mediophyceae
V4
V9
0.8-5/2000
3/5-20
20-180
180-2000
lat
long
50
0
-50
-100
100
200
% euk reads
0.005
0.010
0.015
0.020
0.025
expShannon
1.8
1.6
1.4
1.2
1.0

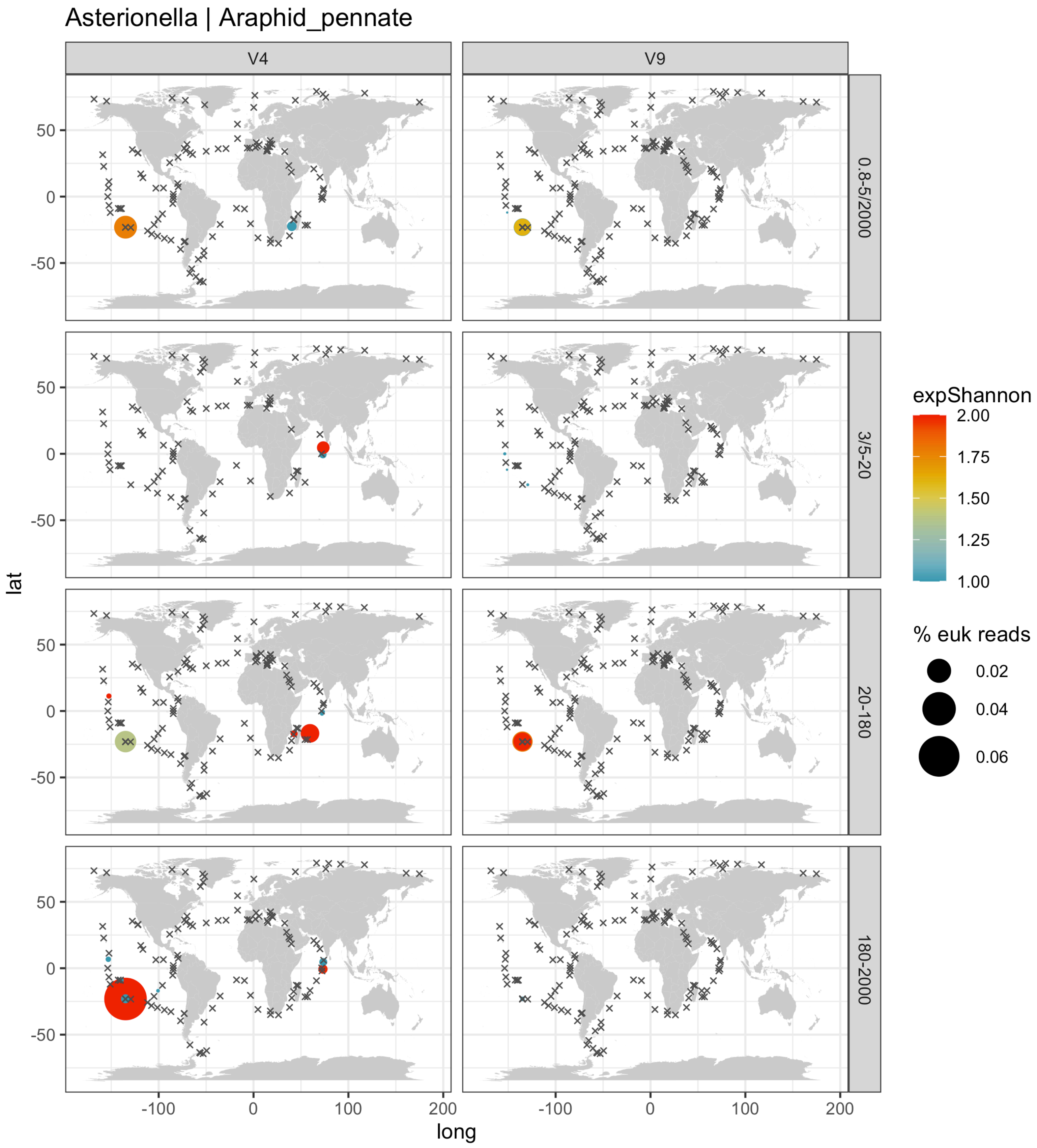

Asterionella | Araphid_pennate
V4
V9
0.8-5/2000
3/5-20
20-180
180-2000
lat
long
50
0
-50
-100
100
200
expShannon
2.00
1.75
1.50
1.25
1.00
% euk reads
0.02
0.04
0.06

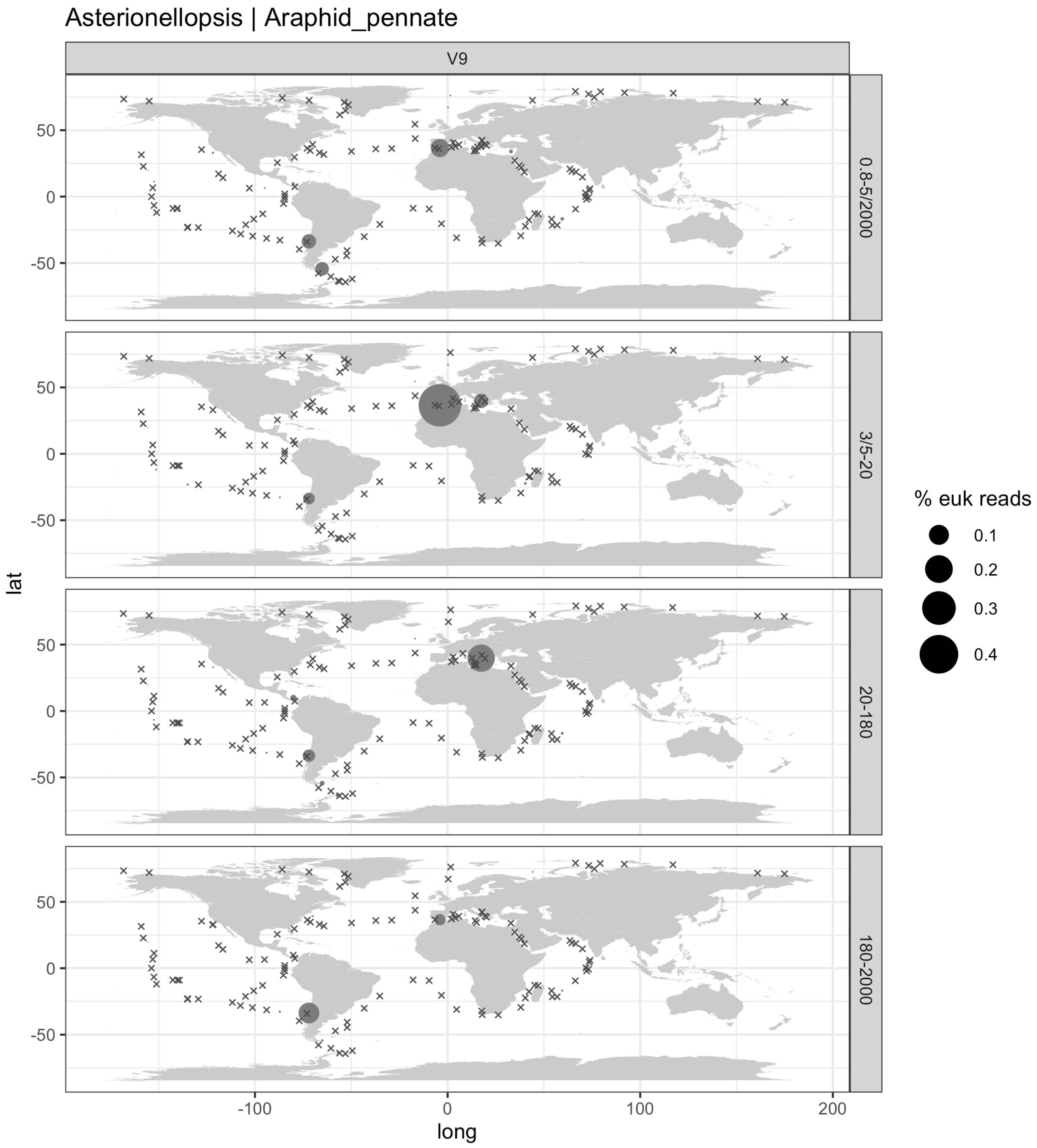

Asterionellopsis | Araphid_pennate
V9
0.8-5/2000
3/5-20
20-180
180-2000
lat
long
50
0
-50
-100
0
100
200
% euk reads
0.1
0.2
0.3
0.4

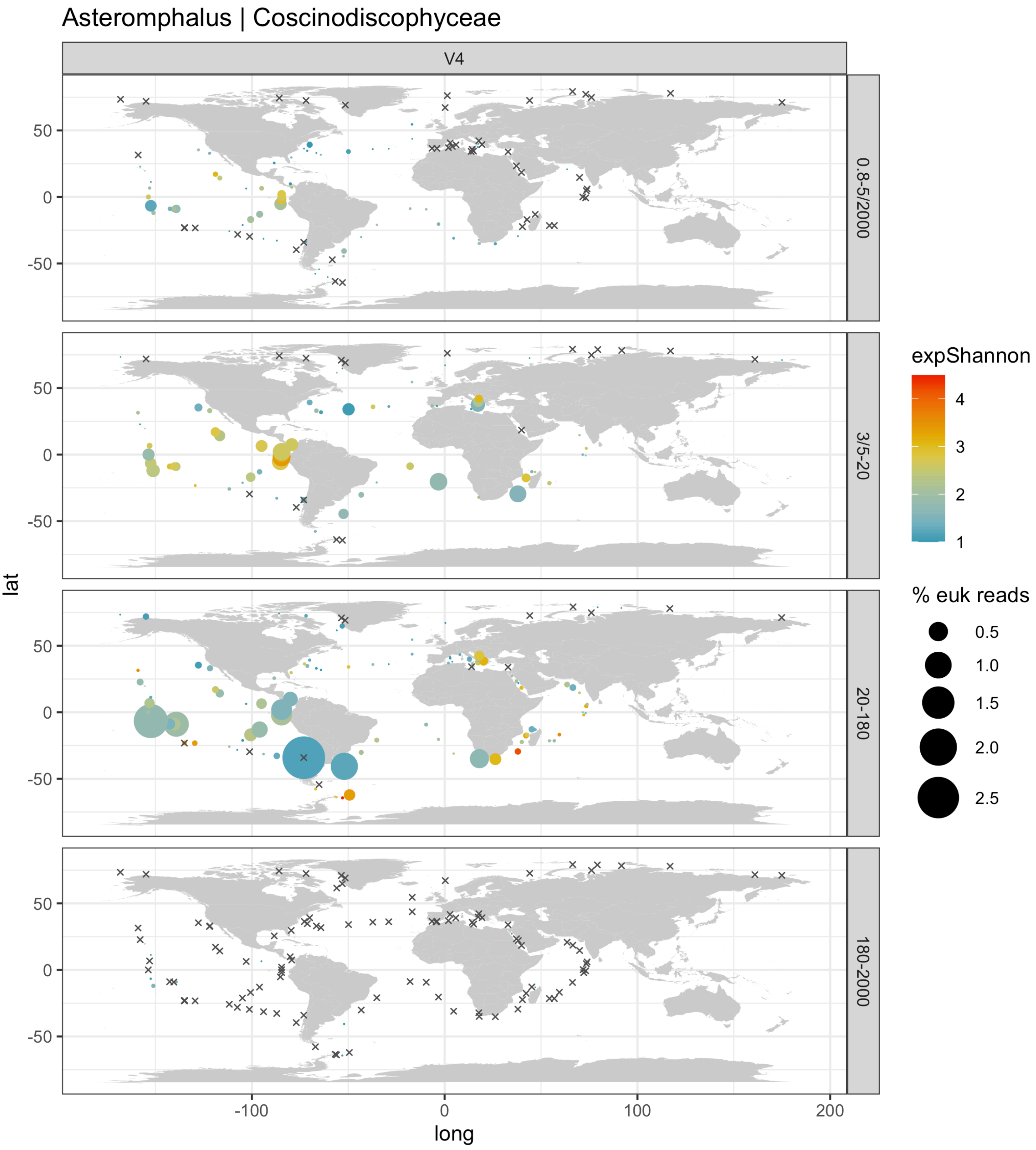

Asteromphalus | Coscinodiscophyceae
V4
0.8-5/2000
3/5-20
20-180
180-2000
lat
long
50
0
-50
-100
0
100
200
expShannon
4
3
2
1
% euk reads
0.5
1.0
1.5
2.0
2.5

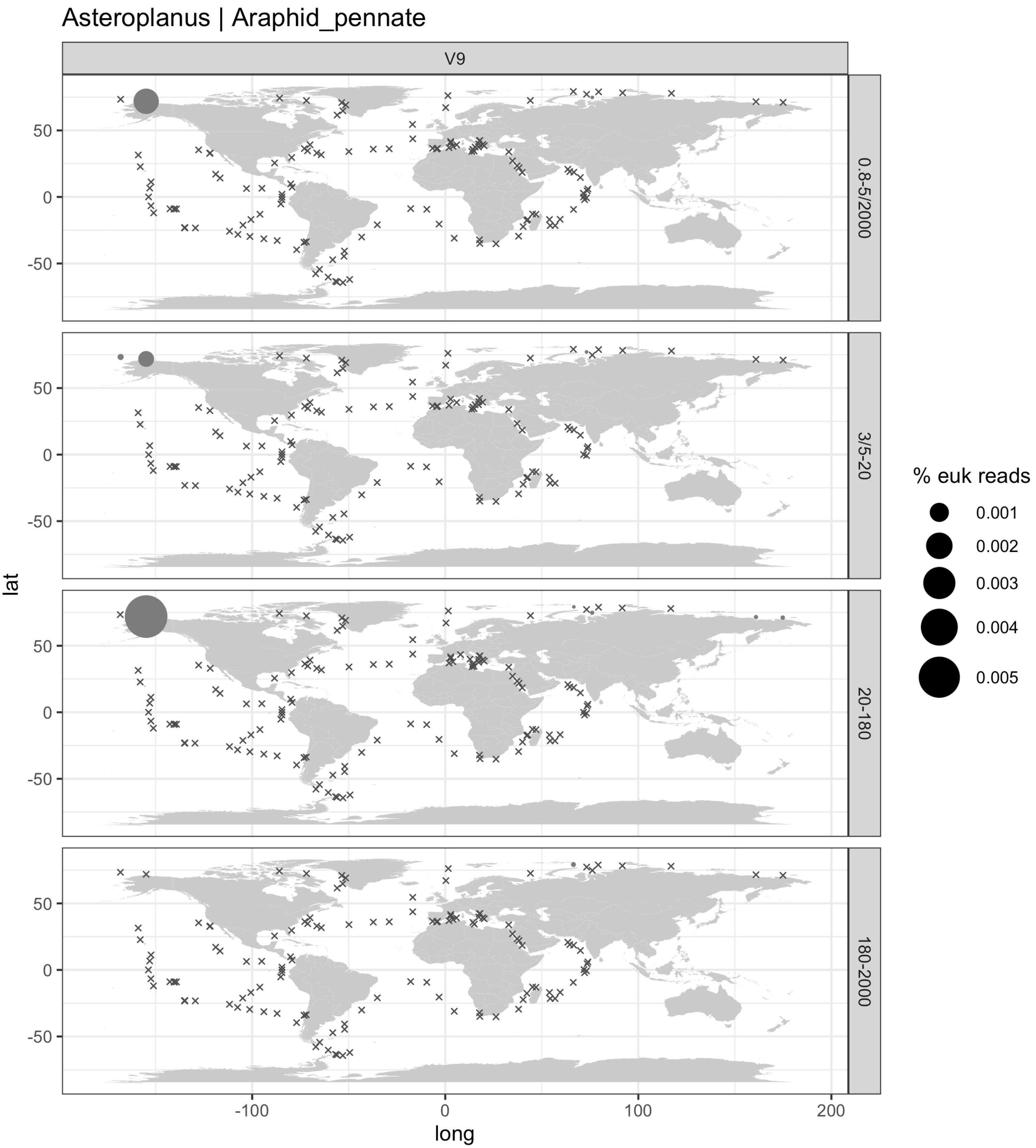

Asteroplanus | Araphid_pennate
V9
0.8-5/2000
3/5-20
20-180
180-2000
lat
long
50
0
-50
-100
0
100
200
% euk reads
0.001
0.002
0.003
0.004
0.005

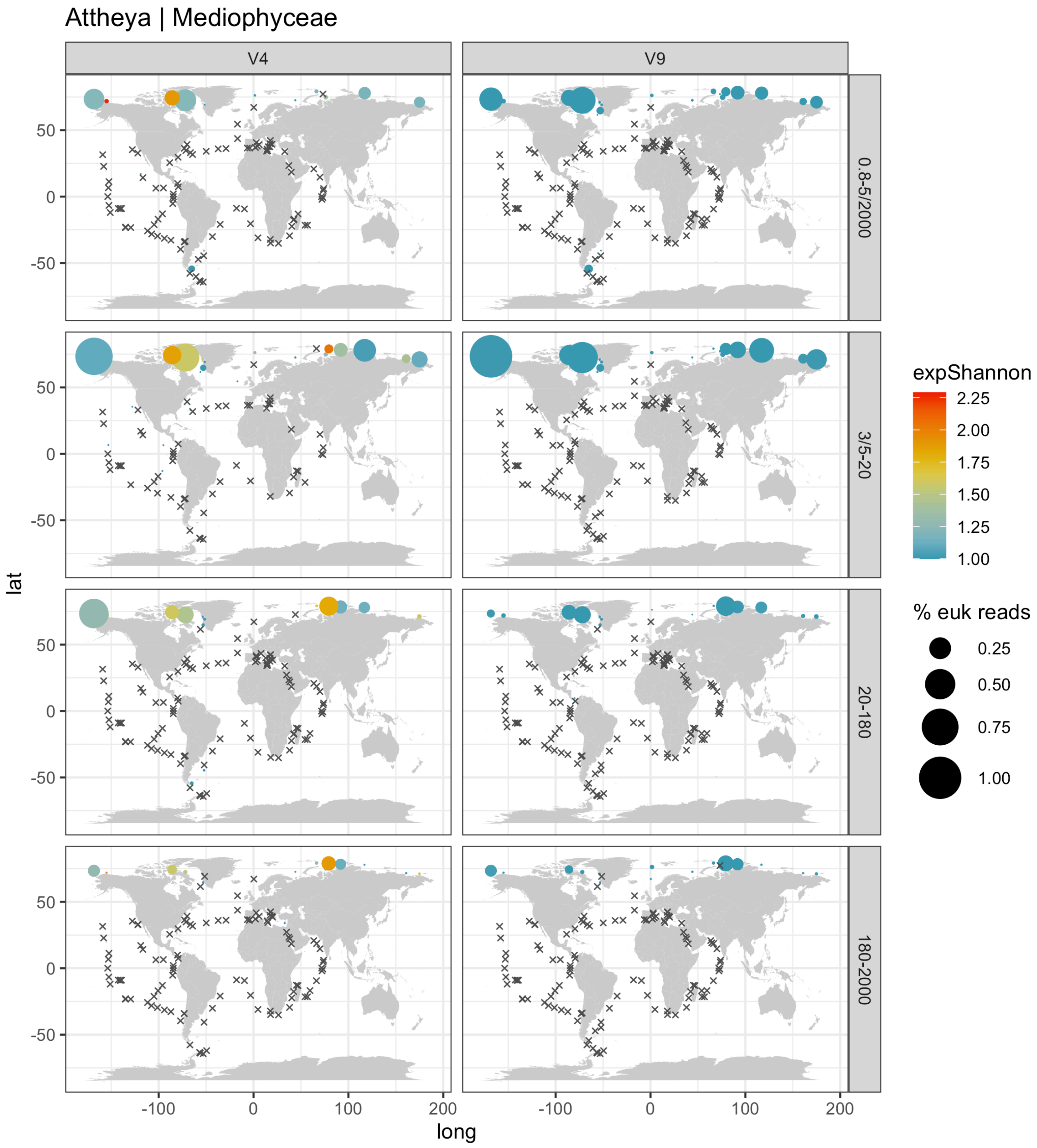

Attheya | Mediophyceae
V4
V9
0.8-5/2000
3/5-20
20-180
180-2000
lat
long
50
0
-50
-100
0
100
200
expShannon
2.25
2.00
1.75
1.50
1.25
1.00
% euk reads
0.25
0.50
0.75
1.00

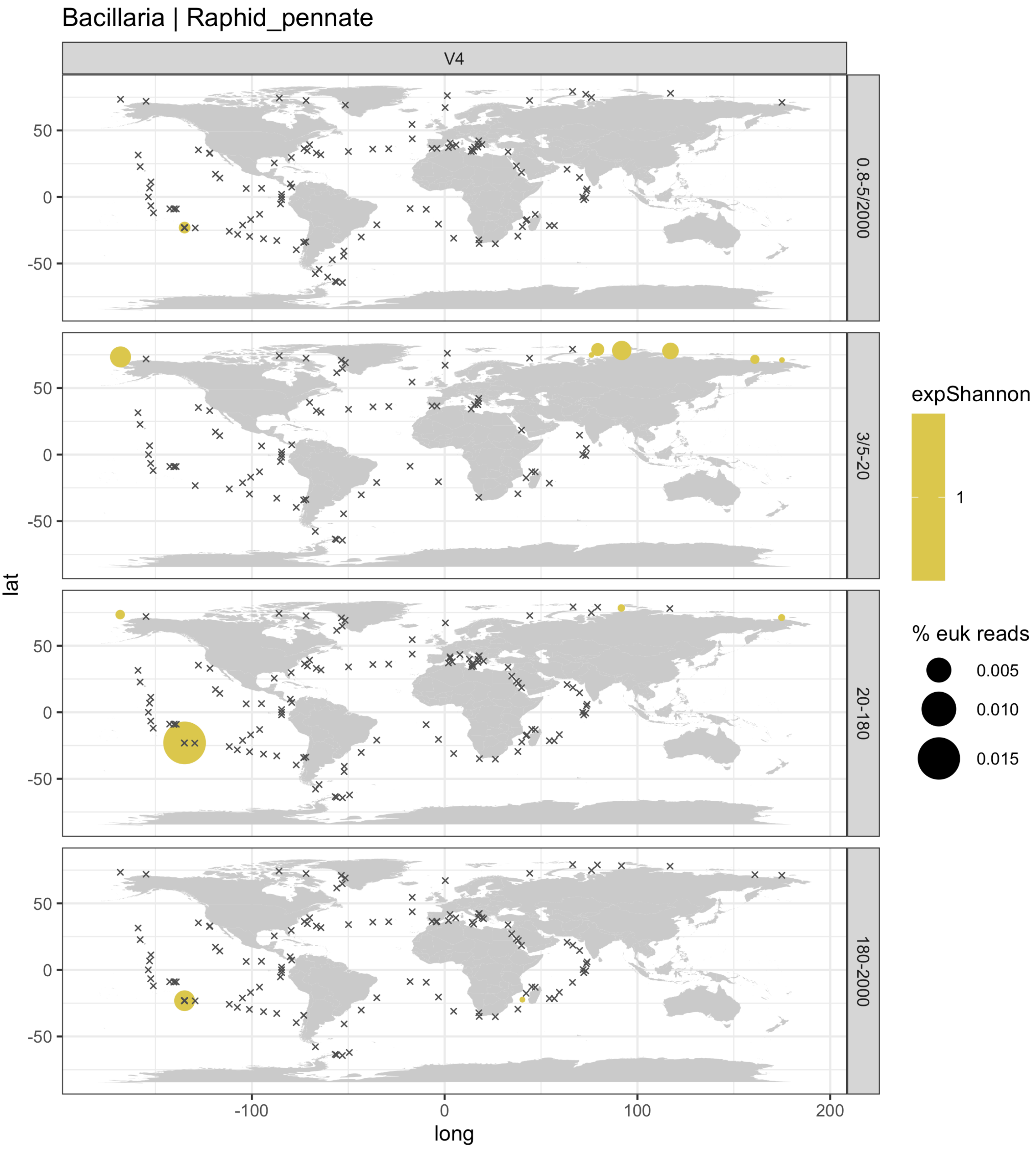

Bacillaria | Raphid_pennate
V4
0.8-5/2000
3/5-20
20-180
180-2000
lat
long
-100
0
100
200
50
0
-50
expShannon
1
% euk reads
0.005
0.010
0.015

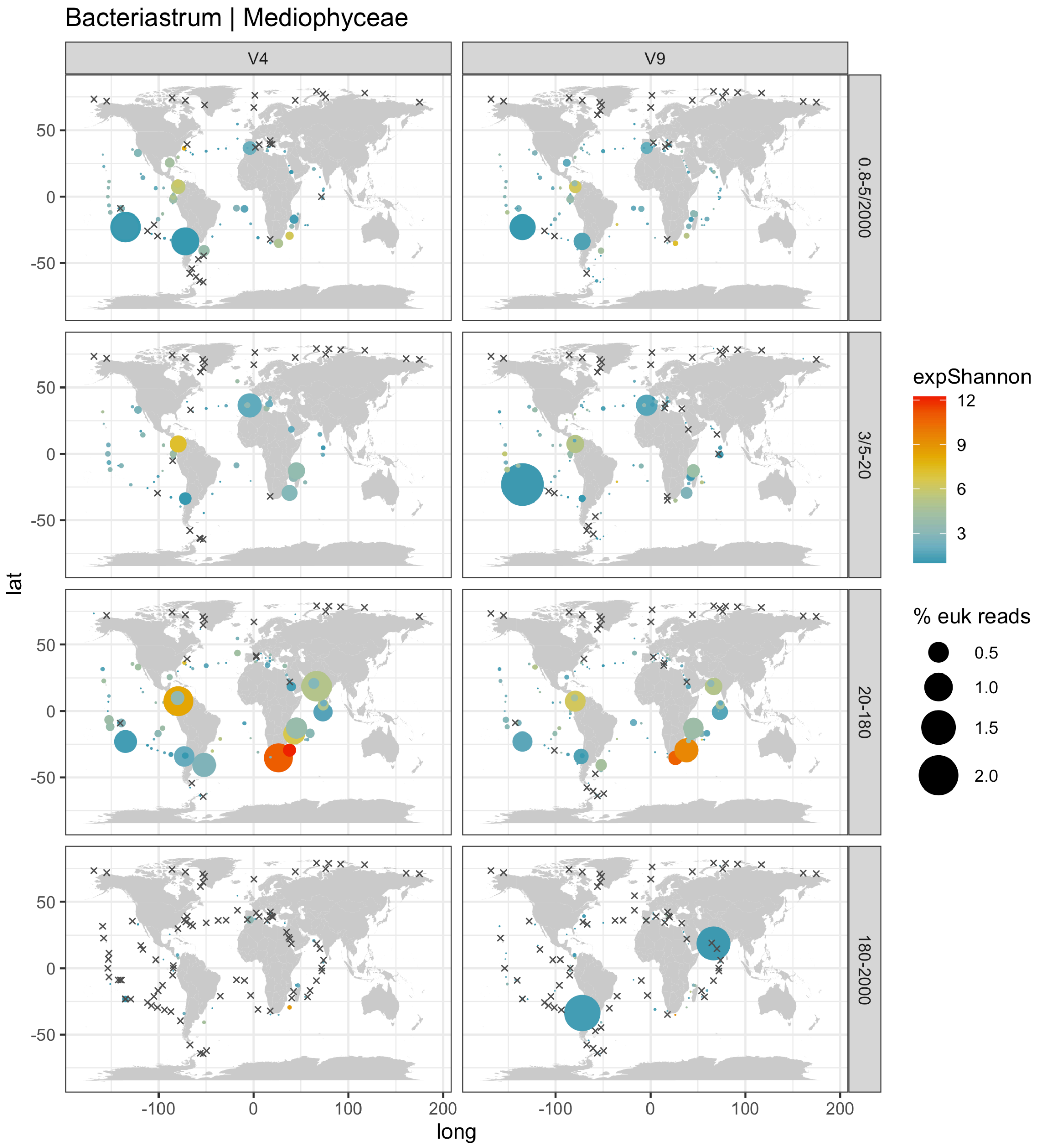

Bacteriastrum | Mediophyceae
V4
V9
0.8-5/2000
3/5-20
20-180
180-2000
lat
long
50
0
-50
-100
0
100
200
expShannon
12
9
6
3
% euk reads
0.5
1.0
1.5
2.0

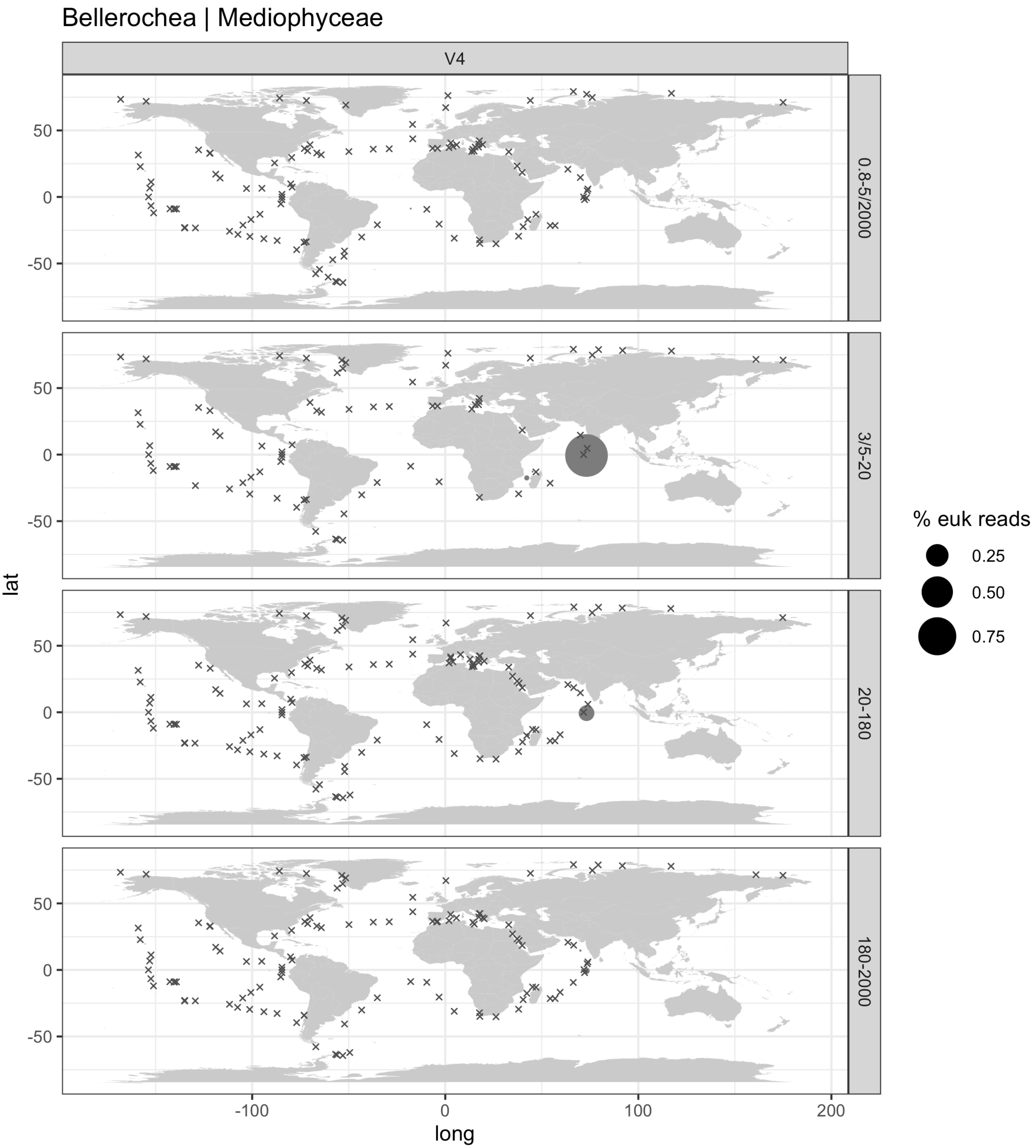

Bellerochea | Mediophyceae
V4
0.8-5/2000
3/5-20
20-180
180-2000
lat
long
50
0
-50
-100
0
100
200
% euk reads
0.25
0.50
0.75

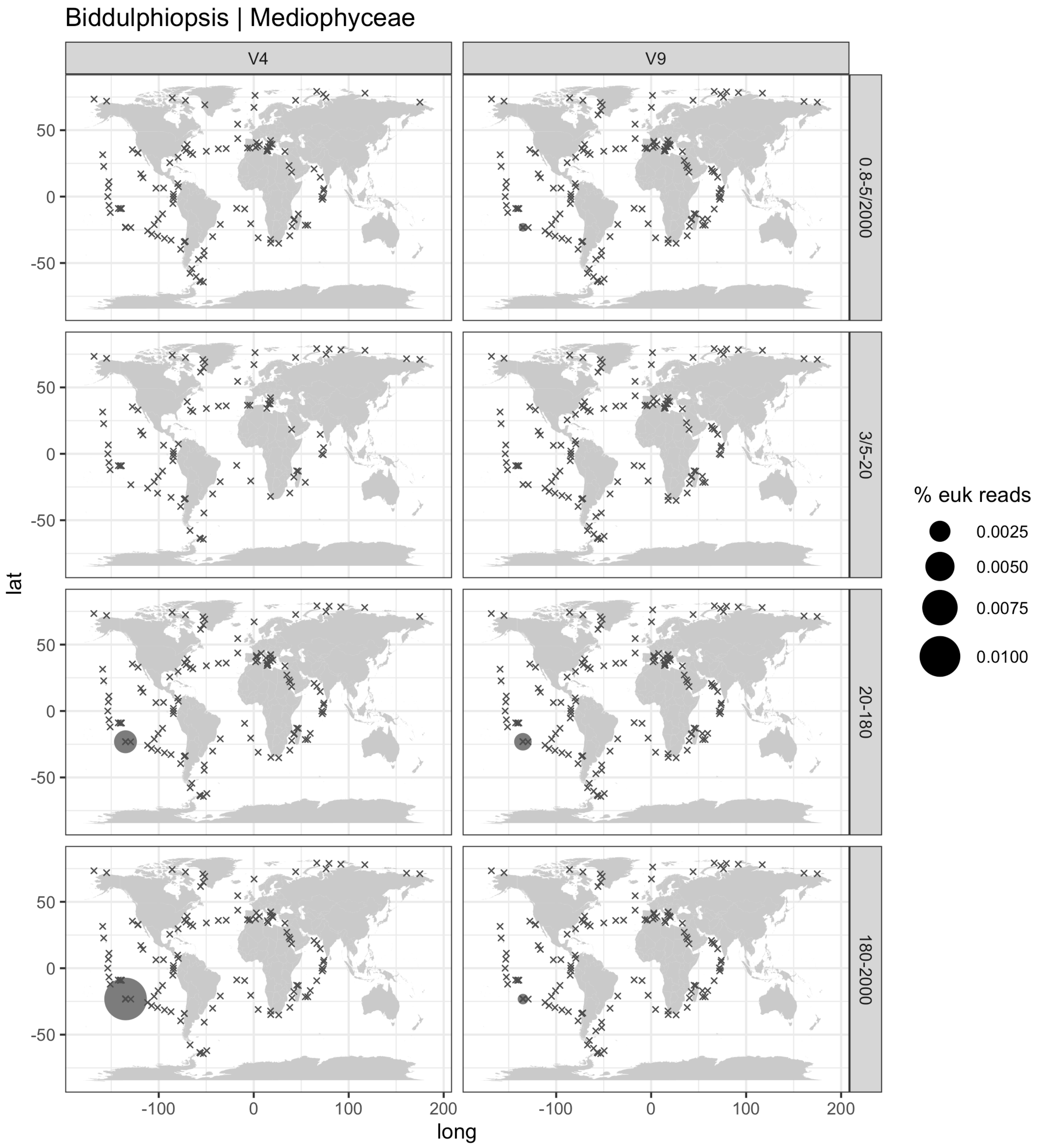

Biddulphiopsis | Mediophyceae
V4
V9
0.8-5/2000
3/5-20
20-180
180-2000
lat
long
50
0
-50
-100
0
100
200
% euk reads
0.0025
0.0050
0.0075
0.0100

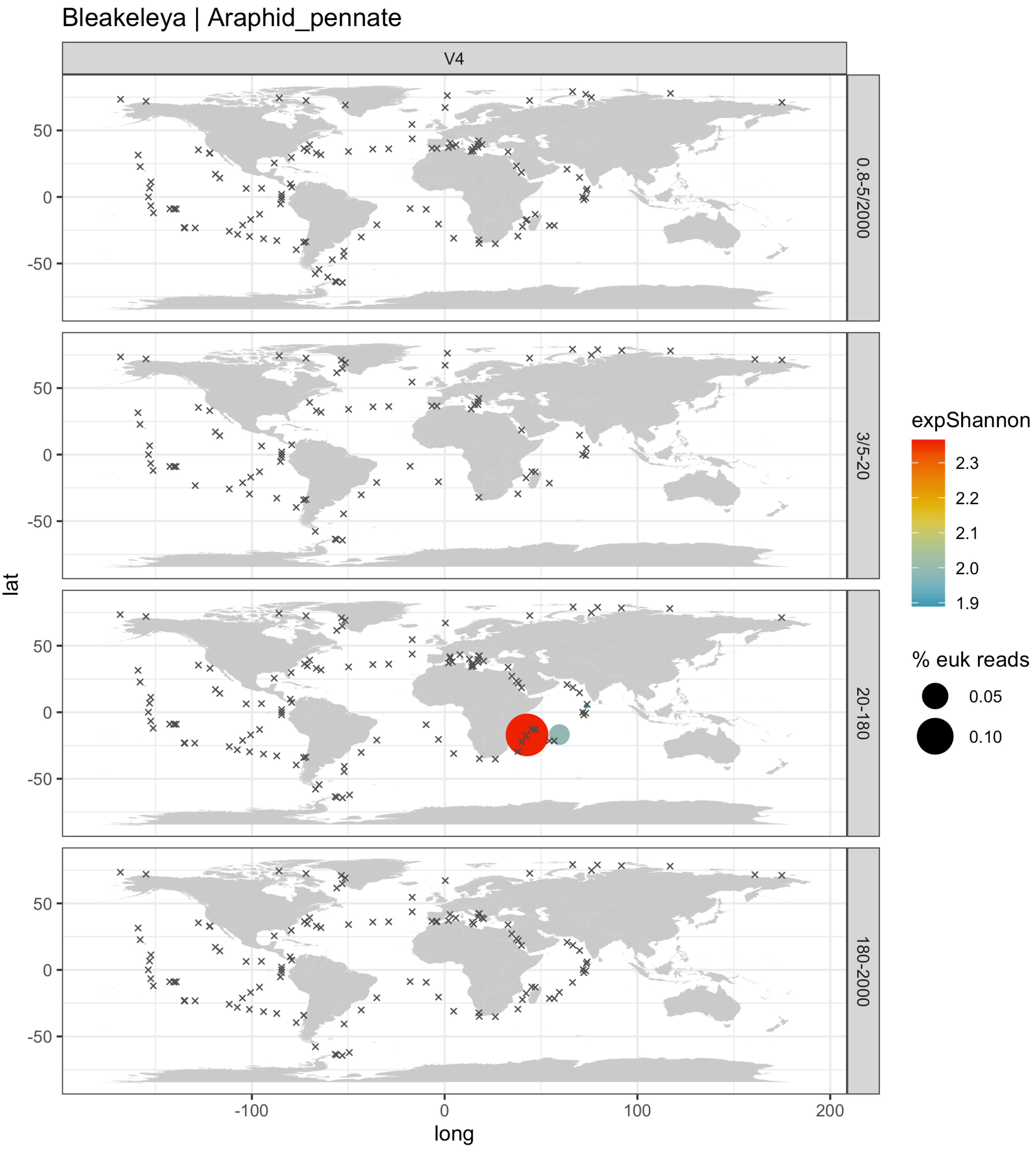

Bleakeleya | Araphid_pennate
V4
0.8-5/2000
3/5-20
20-180
180-2000
lat
long
50
0
-50
-100
0
100
200
expShannon
2.3
2.2
2.1
2.0
1.9
% euk reads
0.05
0.10

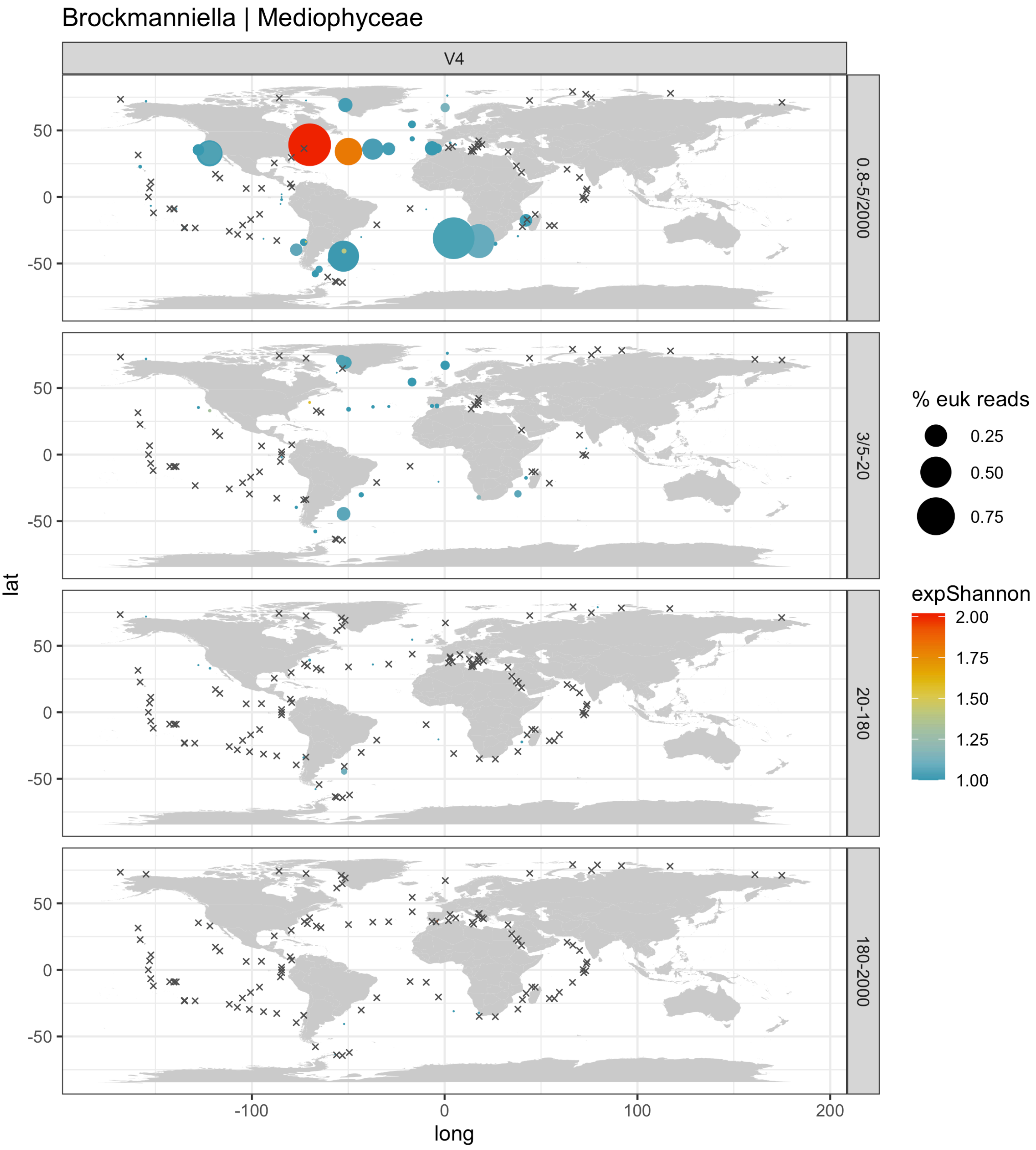

Brockmanniella | Mediophyceae
V4
0.8-5/2000
3/5-20
20-180
180-2000
lat
long
50
0
-50
-100
100
200
% euk reads
0.25
0.50
0.75
expShannon
2.00
1.75
1.50
1.25
1.00

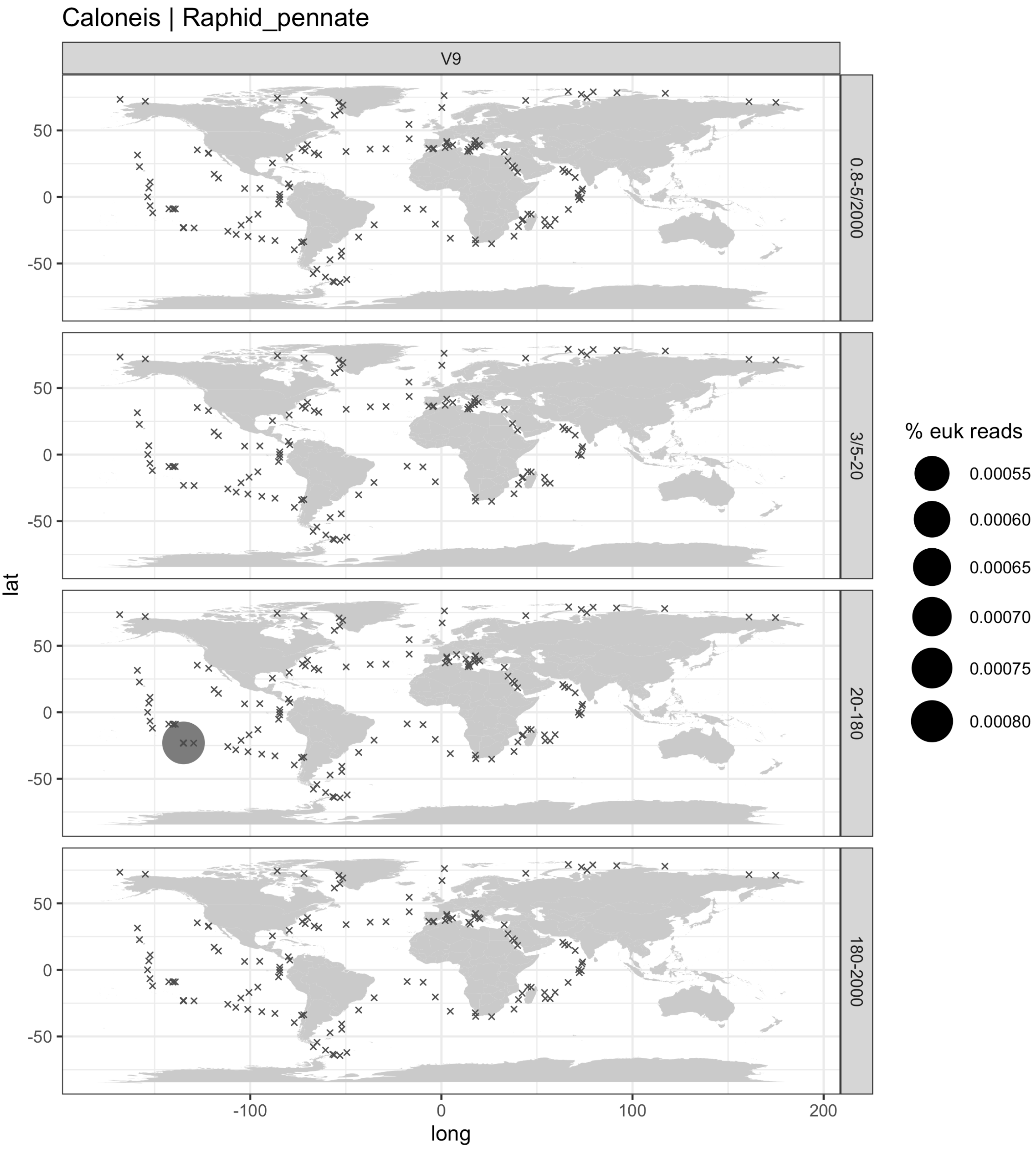

Caloneis | Raphid_pennate
V9
0.8-5/2000
3/5-20
20-180
180-2000
lat
long
50
0
-50
-100
100
200
% euk reads
0.00055
0.00060
0.00065
0.00070
0.00075
0.00080

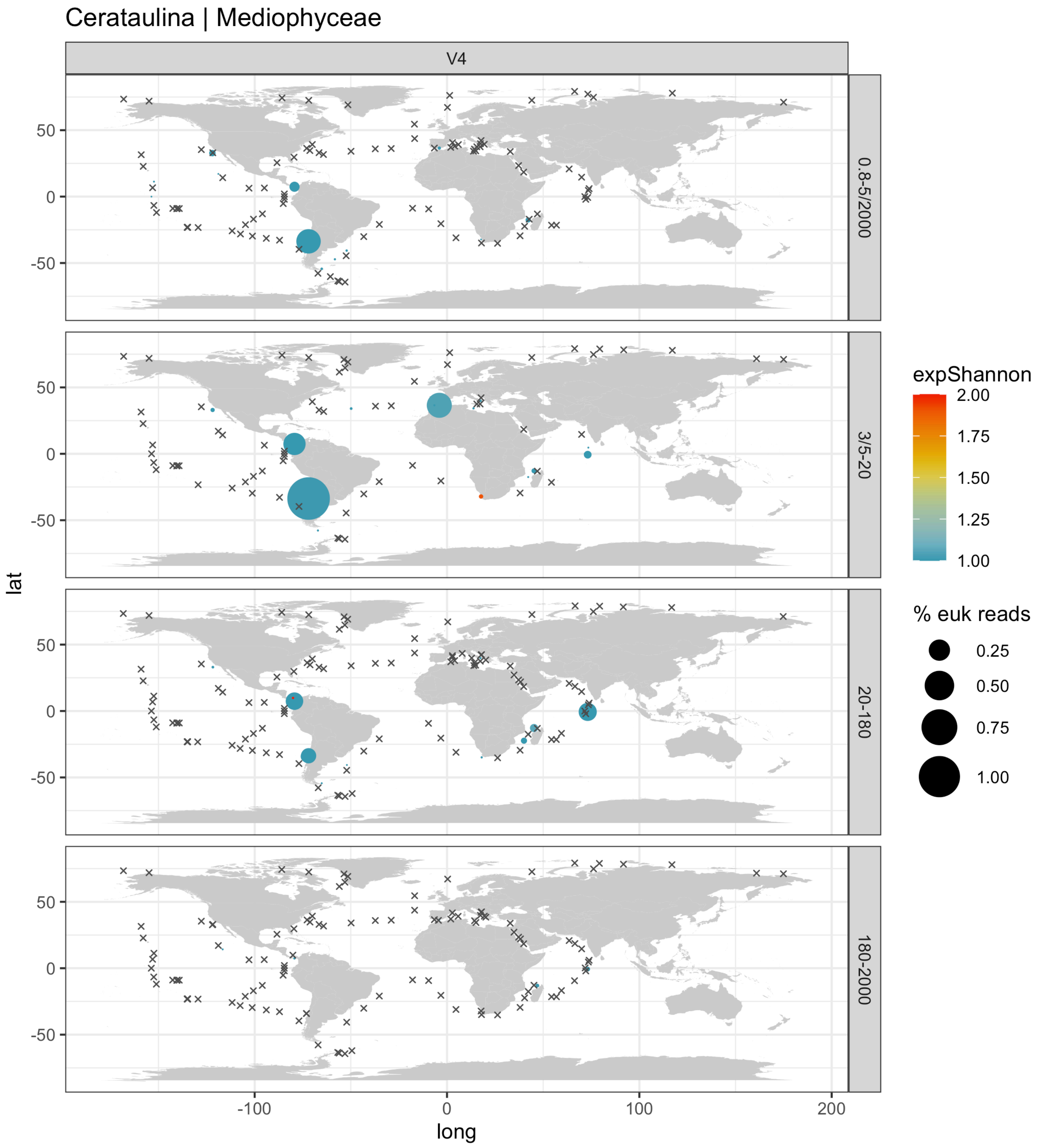

Cerataulina | Mediophyceae
V4
0.8-5/2000
3/5-20
20-180
180-2000
lat
long
50
0
-50
-100
0
100
200
expShannon
2.00
1.75
1.50
1.25
1.00
% euk reads
0.25
0.50
0.75
1.00

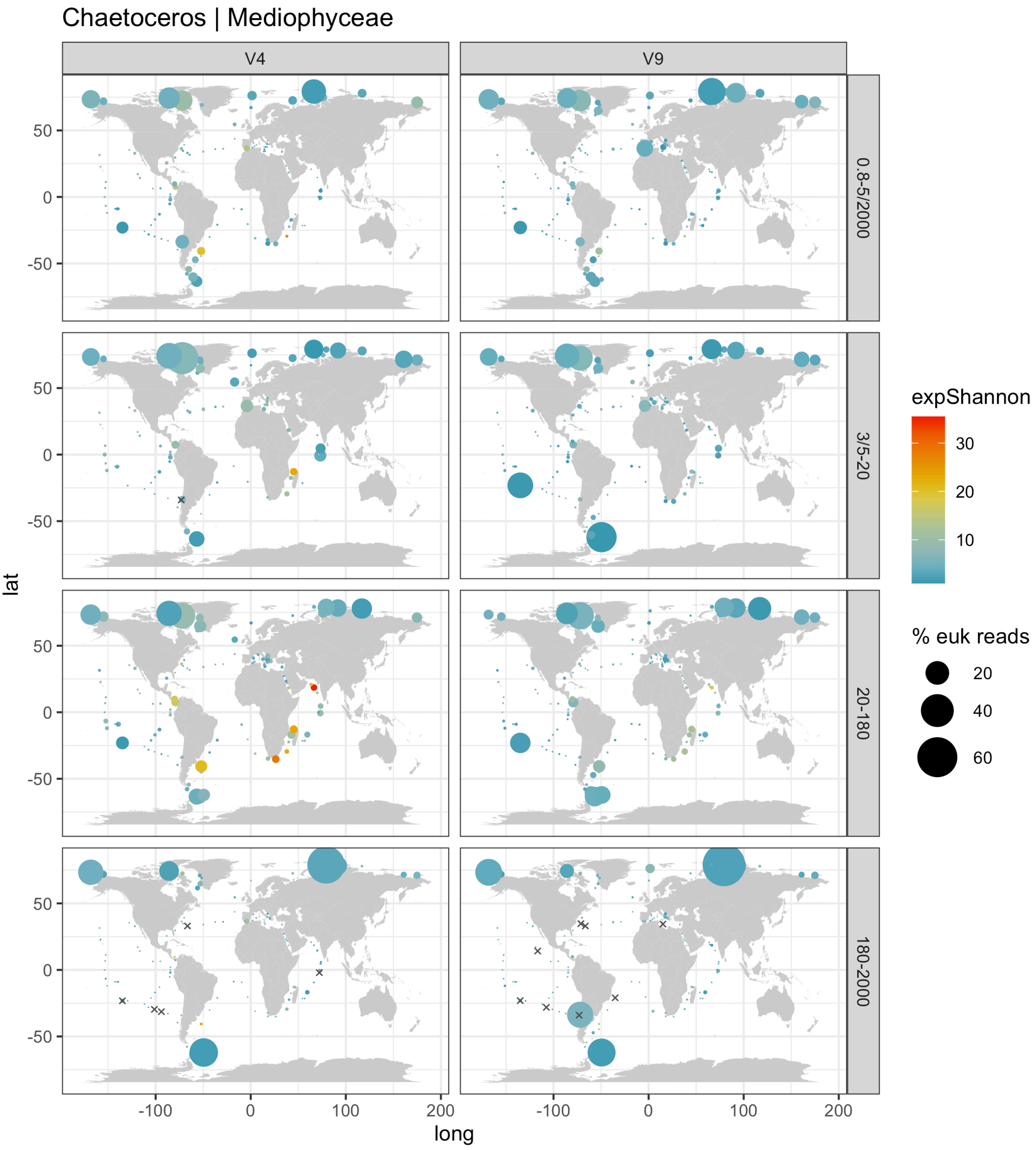

Chaetoceros | Mediophyceae
V4
V9
0.8-5/2000
3/5-20
20-180
180-2000
lat
long
50
0
-50
-100
100
200
expShannon
30
20
10
% euk reads
20
40
60

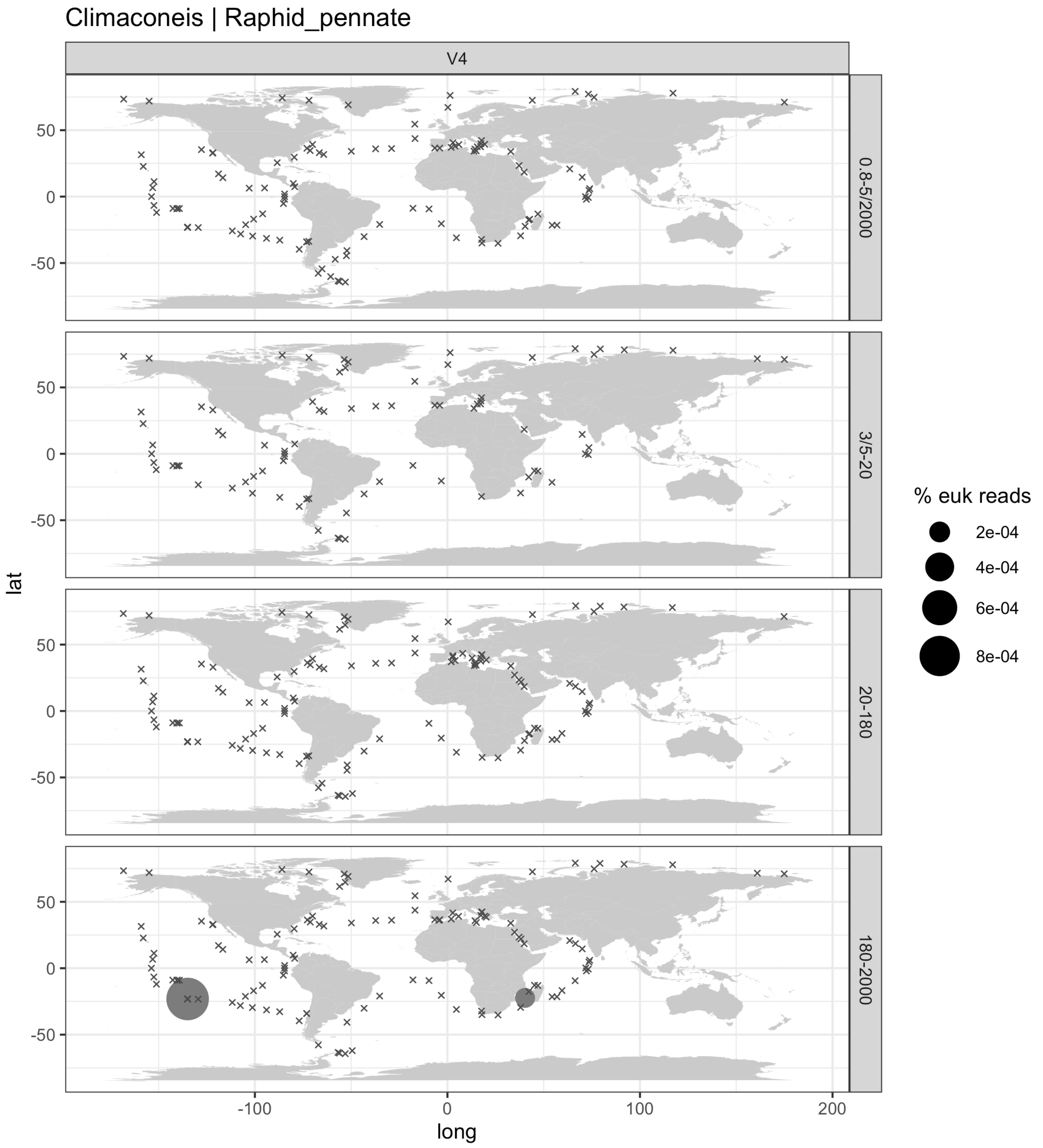

Climaconeis | Raphid_pennate
V4
0.8-5/2000
3/5-20
20-180
180-2000
lat
long
50
0
-50
-100
0
100
200
% euk reads
2e-04
4e-04
6e-04
8e-04

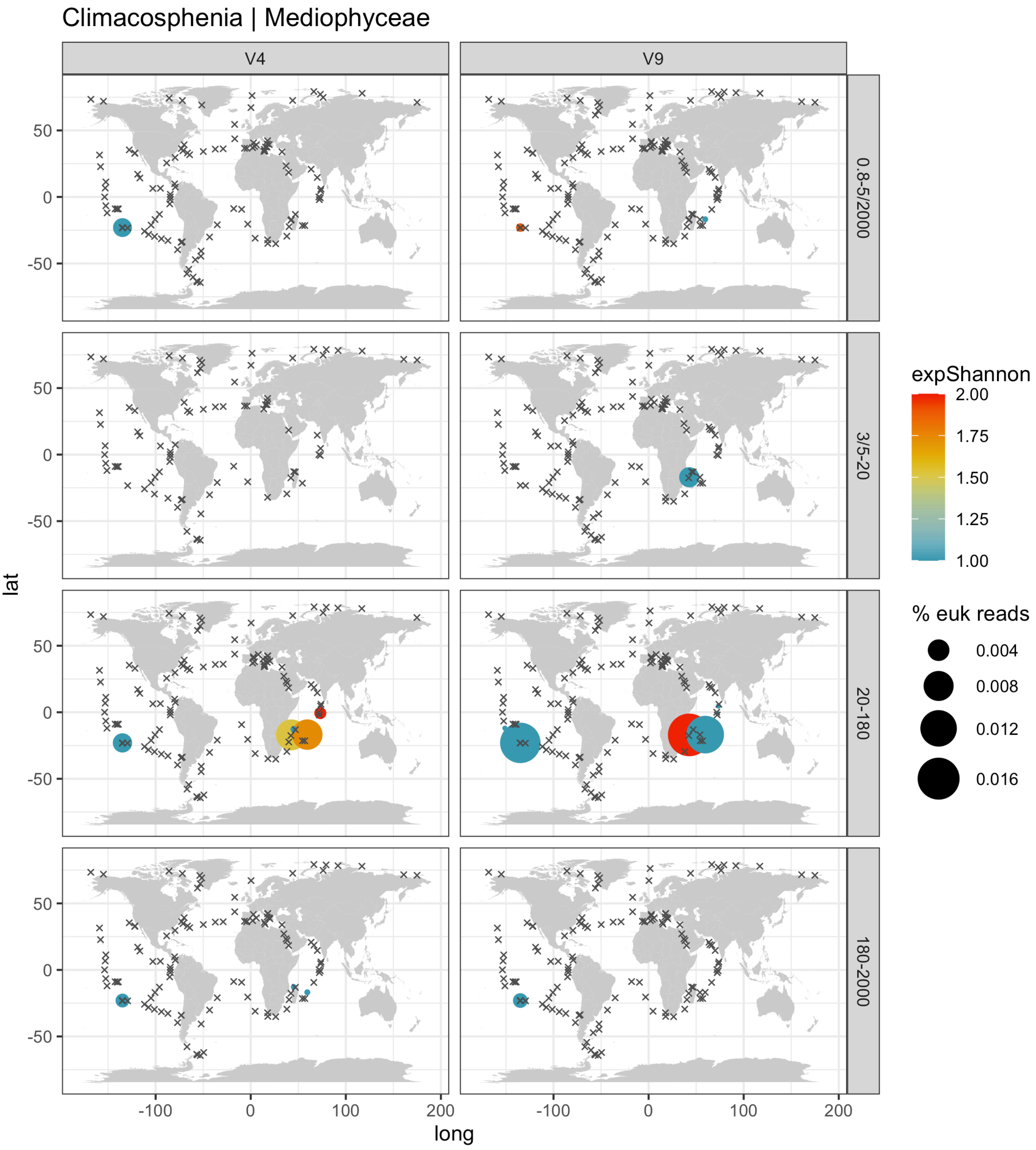
Climacosphenia | Mediophyceae
V4
V9
0.8-5/2000
3/5-20
20-180
180-2000
lat
long
expShannon
2.00
1.75
1.50
1.25
1.00
% euk reads
0.004
0.008
0.012
0.016
50
0
-50
-100
0
100
200

Cocconeis | Raphid_pennate

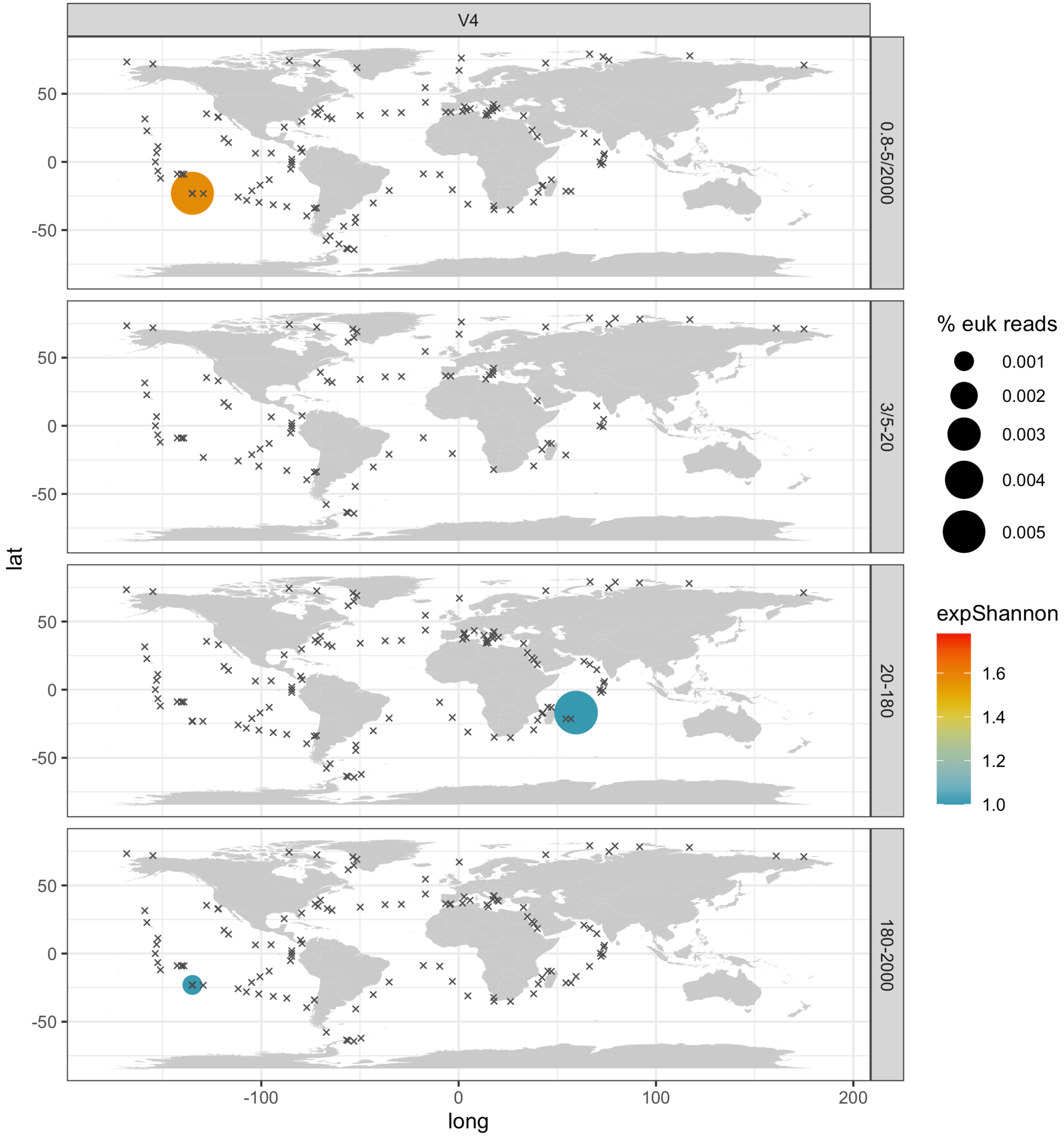

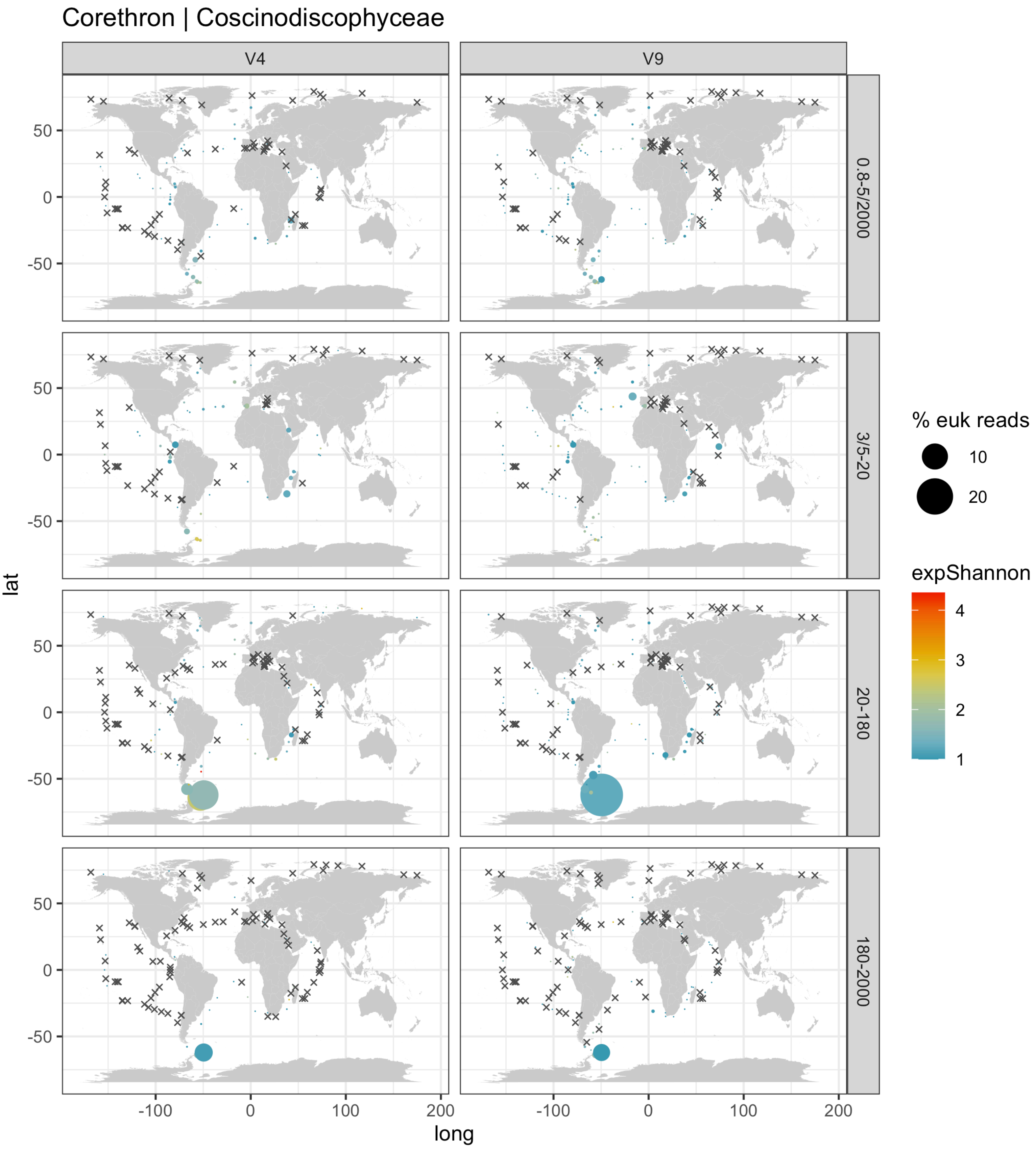

Corethron | Coscinodiscophyceae
V4
V9
0.8-5/2000
3/5-20
20-180
180-2000
lat
long
50
0
-50
-100
100
200
% euk reads
10
20
expShannon
4
3
2
1

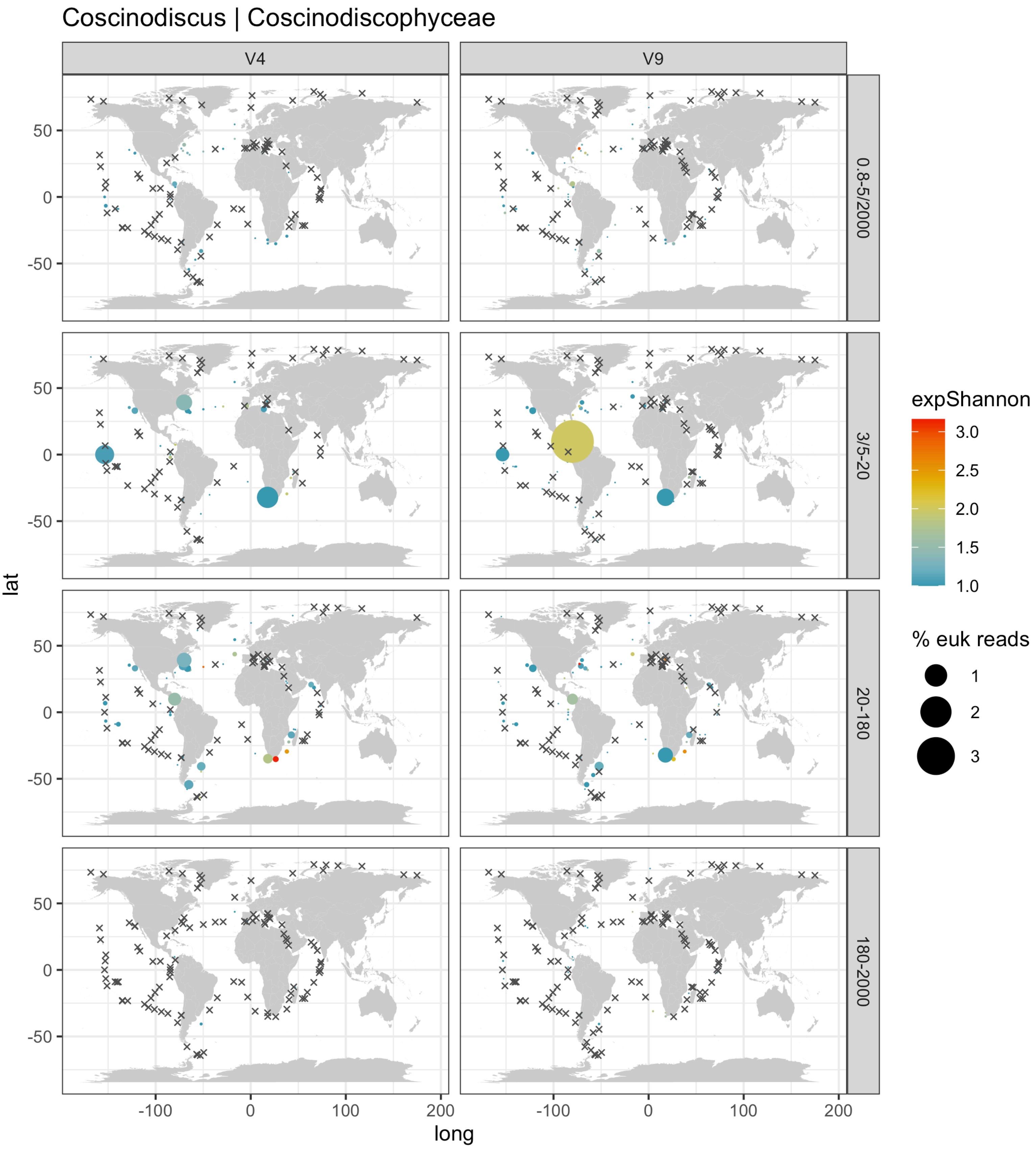
Coscinodiscus | Coscinodiscophyceae
V4
V9
0.8-5/2000
3/5-20
20-180
180-2000
lat
long
50
0
-50
-100
100
200
expShannon
3.0
2.5
2.0
1.5
1.0
% euk reads
1
2
3

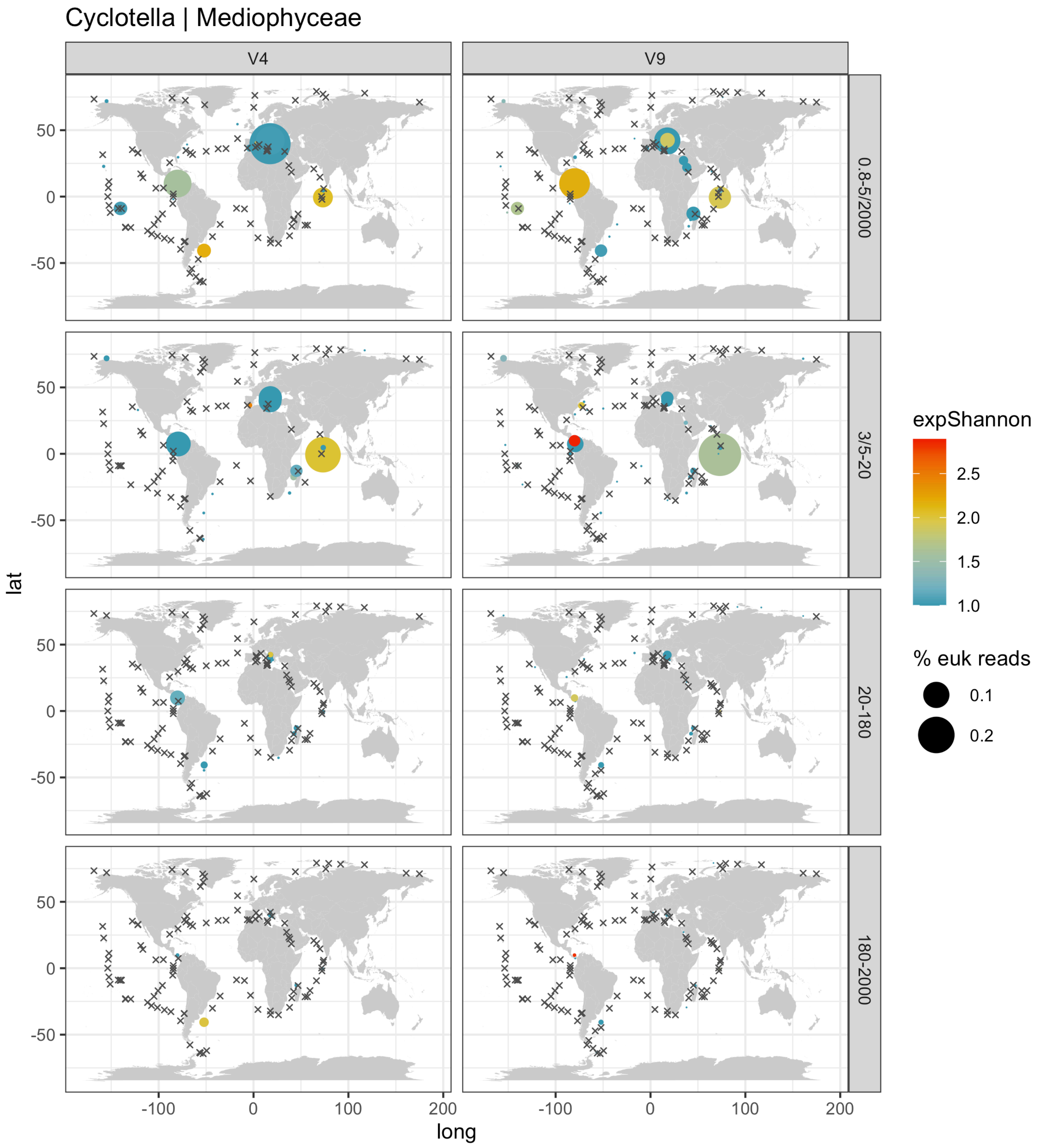

Cyclotella | Mediophyceae
V4
V9
0.8-5/2000
3/5-20
20-180
180-2000
lat
long
50
0
-50
-100
0
100
200
expShannon
2.5
2.0
1.5
1.0
% euk reads
0.1
0.2

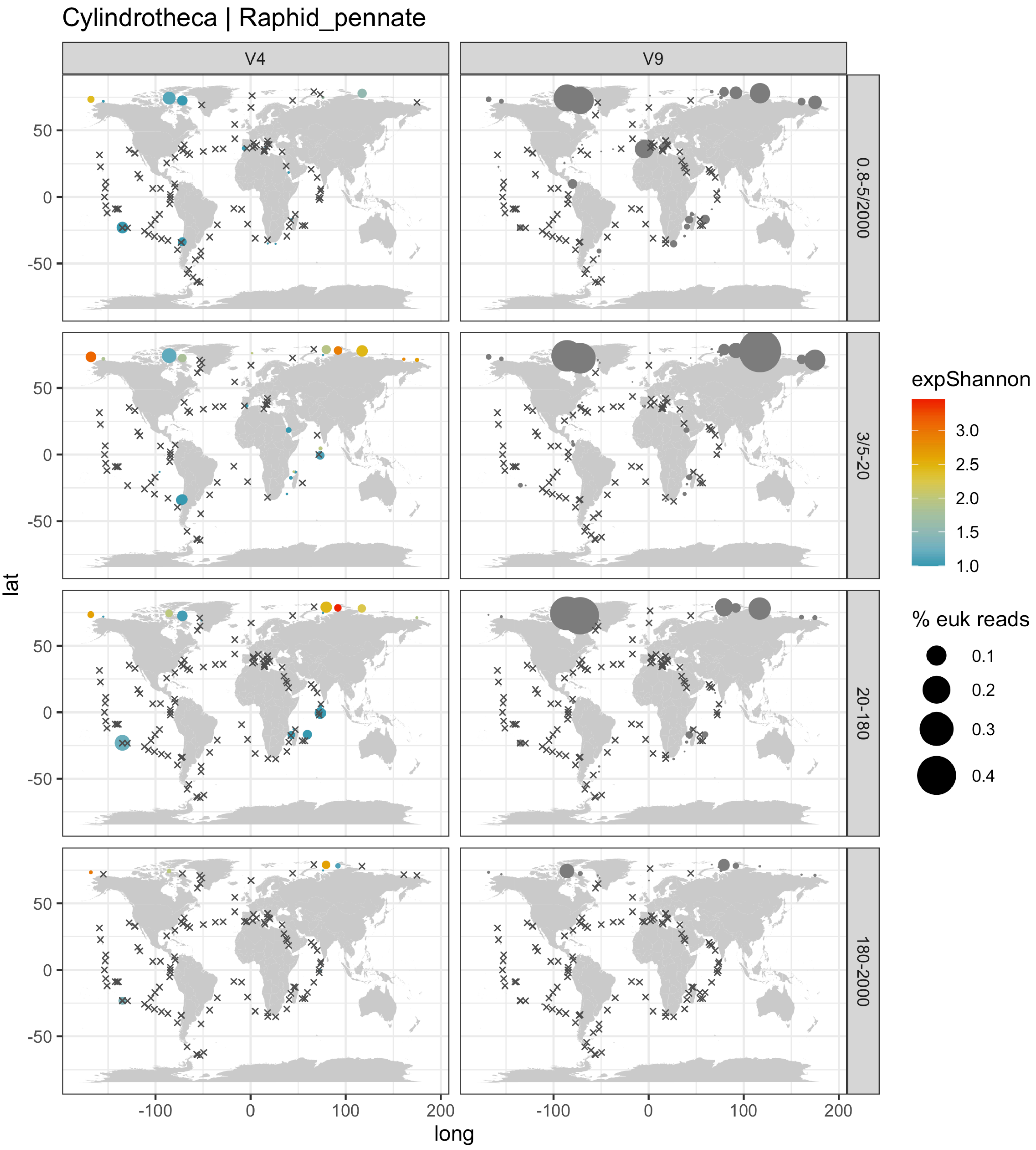

Cylindrotheca | Raphid_pennate
V4
V9
0.8-5/2000
3/5-20
20-180
180-2000
lat
long
50
0
-50
-100
100
200
expShannon
3.0
2.5
2.0
1.5
1.0
% euk reads
0.1
0.2
0.3
0.4

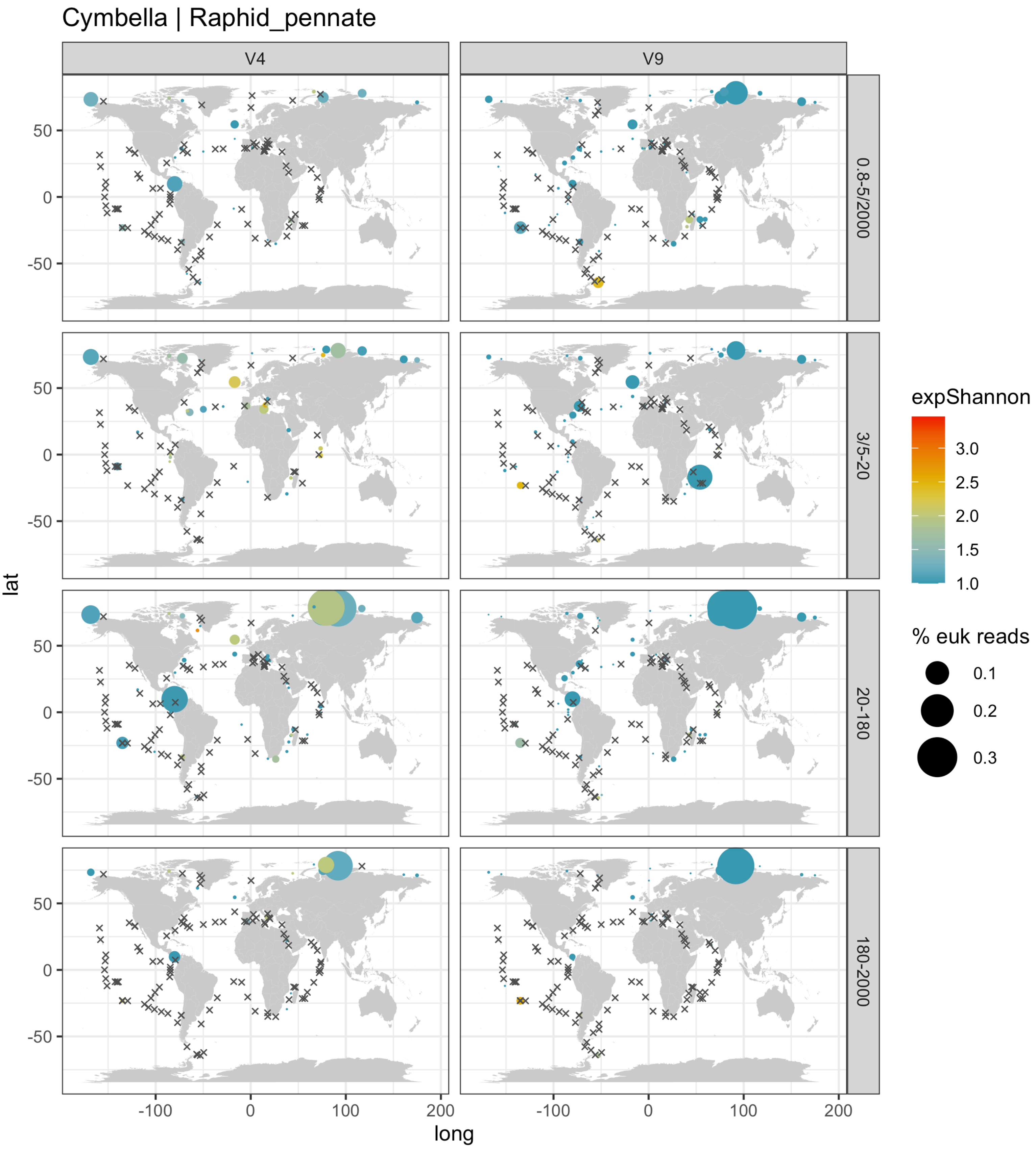

Cymbella | Raphid_pennate
V4
V9
0.8-5/2000
3/5-20
20-180
180-2000
lat
long
50
0
-50
-100
100
200
expShannon
3.0
2.5
2.0
1.5
1.0
% euk reads
0.1
0.2
0.3

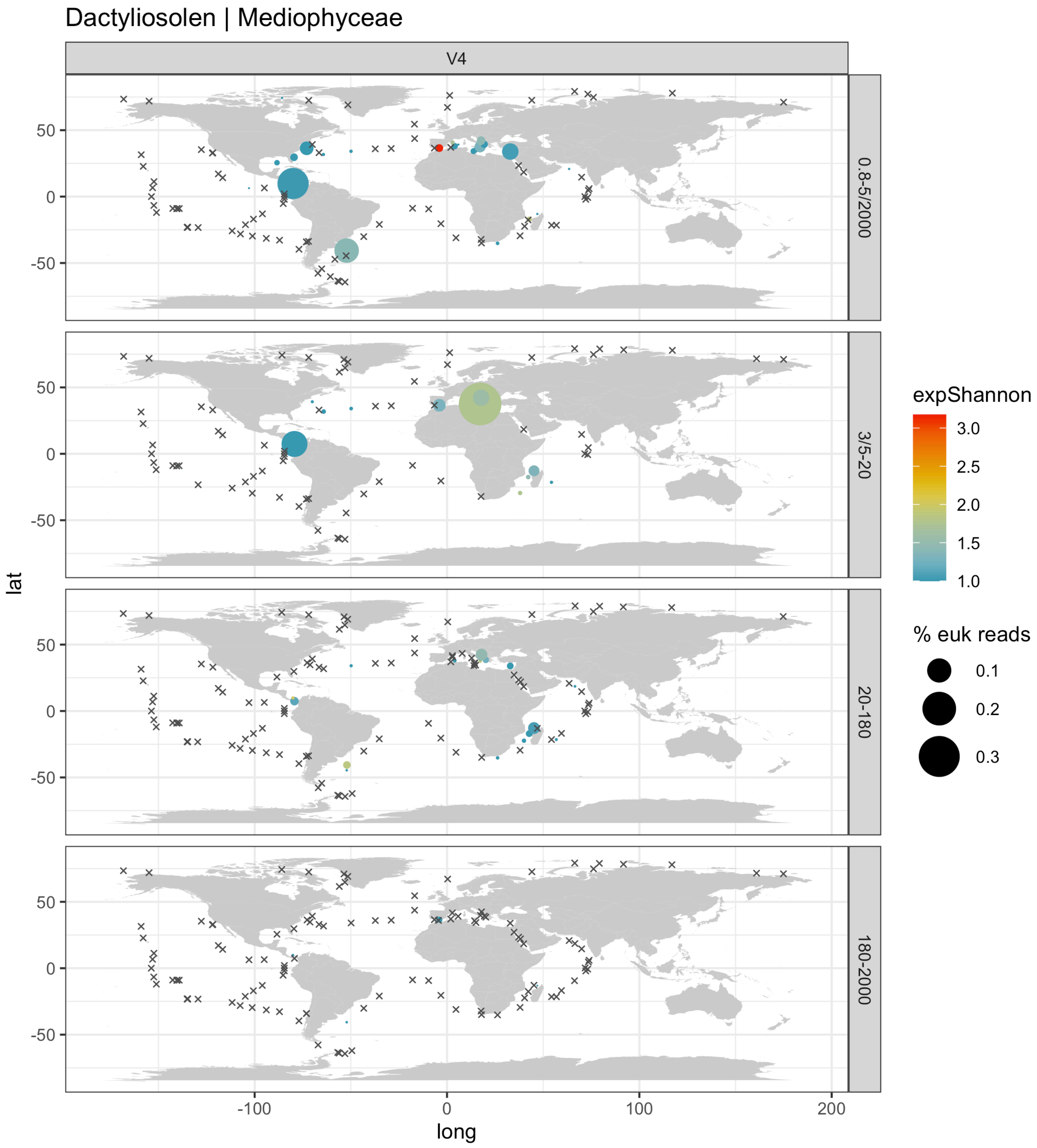

Dactyliosolen | Mediophyceae
V4
0.8-5/2000
3/5-20
20-180
180-2000
lat
long
50
0
-50
-100
100
200
expShannon
3.0
2.5
2.0
1.5
1.0
% euk reads
0.1
0.2
0.3

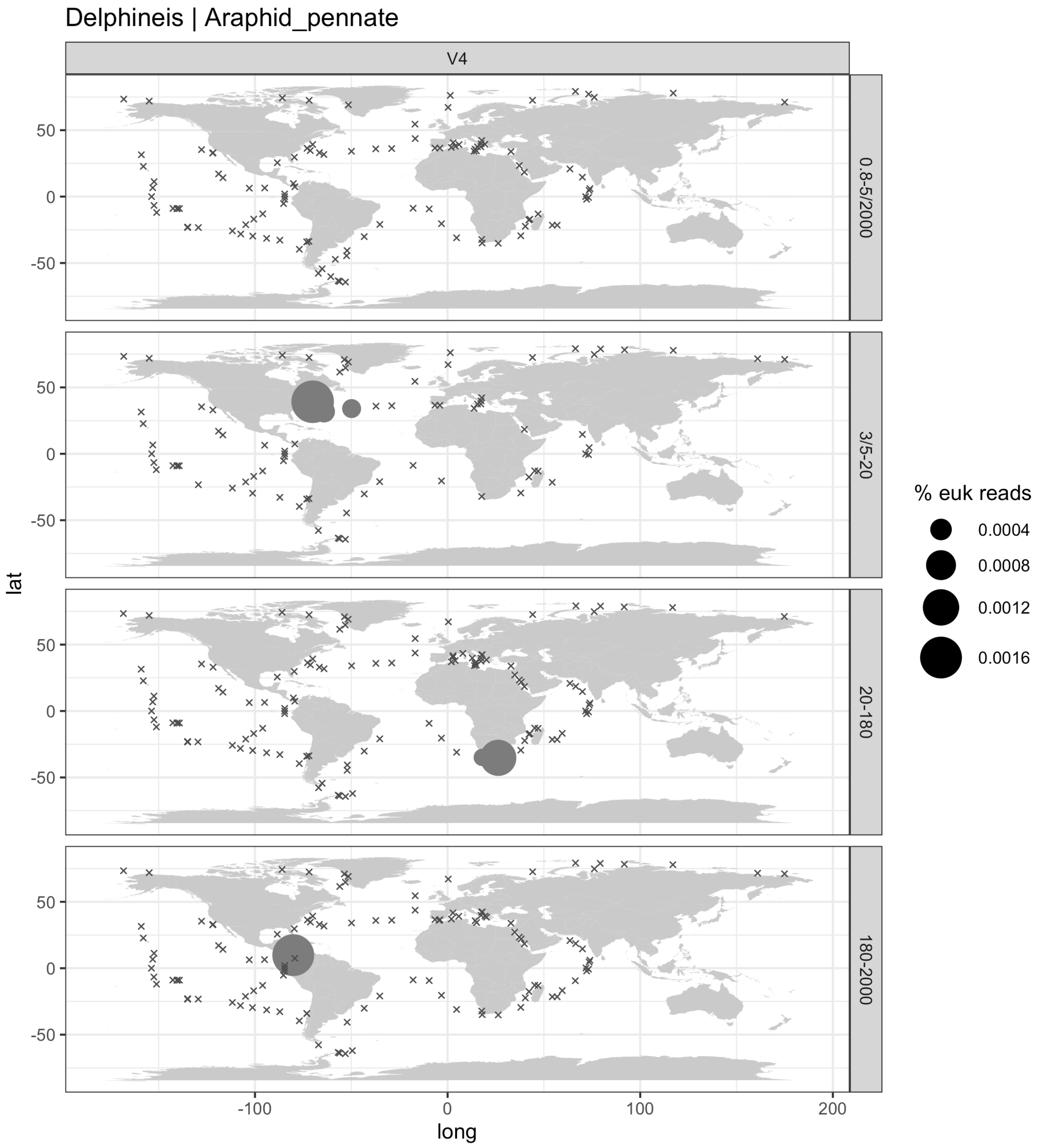

Delphineis | Araphid_pennate
V4
0.8-5/2000
3/5-20
20-180
180-2000
50
0
-50
lat
-100
0
100
200
long
% euk reads
0.0004
0.0008
0.0012
0.0016

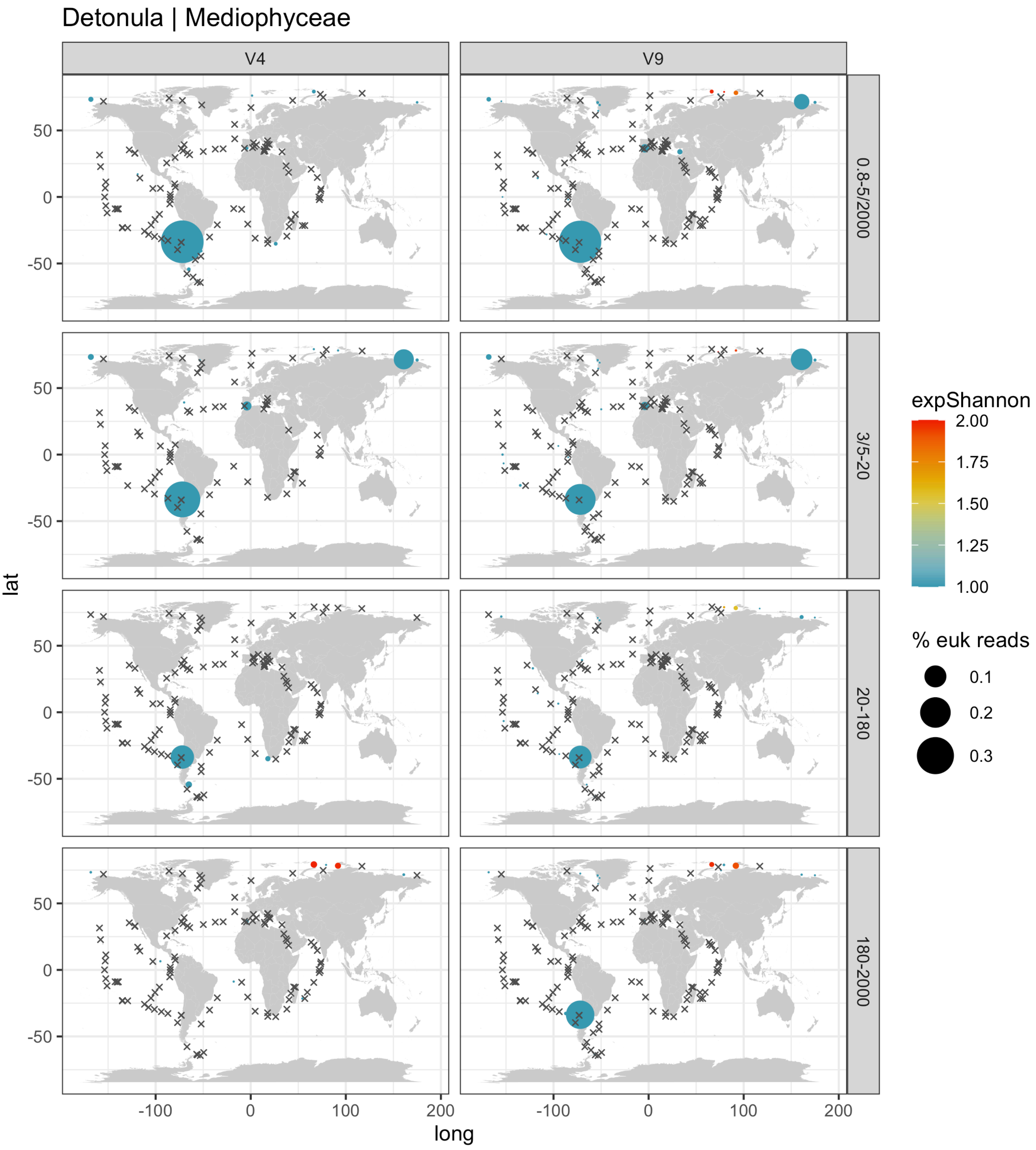

Detonula | Mediophyceae
V4
V9
0.8-5/2000
3/5-20
20-180
180-2000
lat
long
50
0
-50
-100
0
100
200
expShannon
2.00
1.75
1.50
1.25
1.00
% euk reads
0.1
0.2
0.3

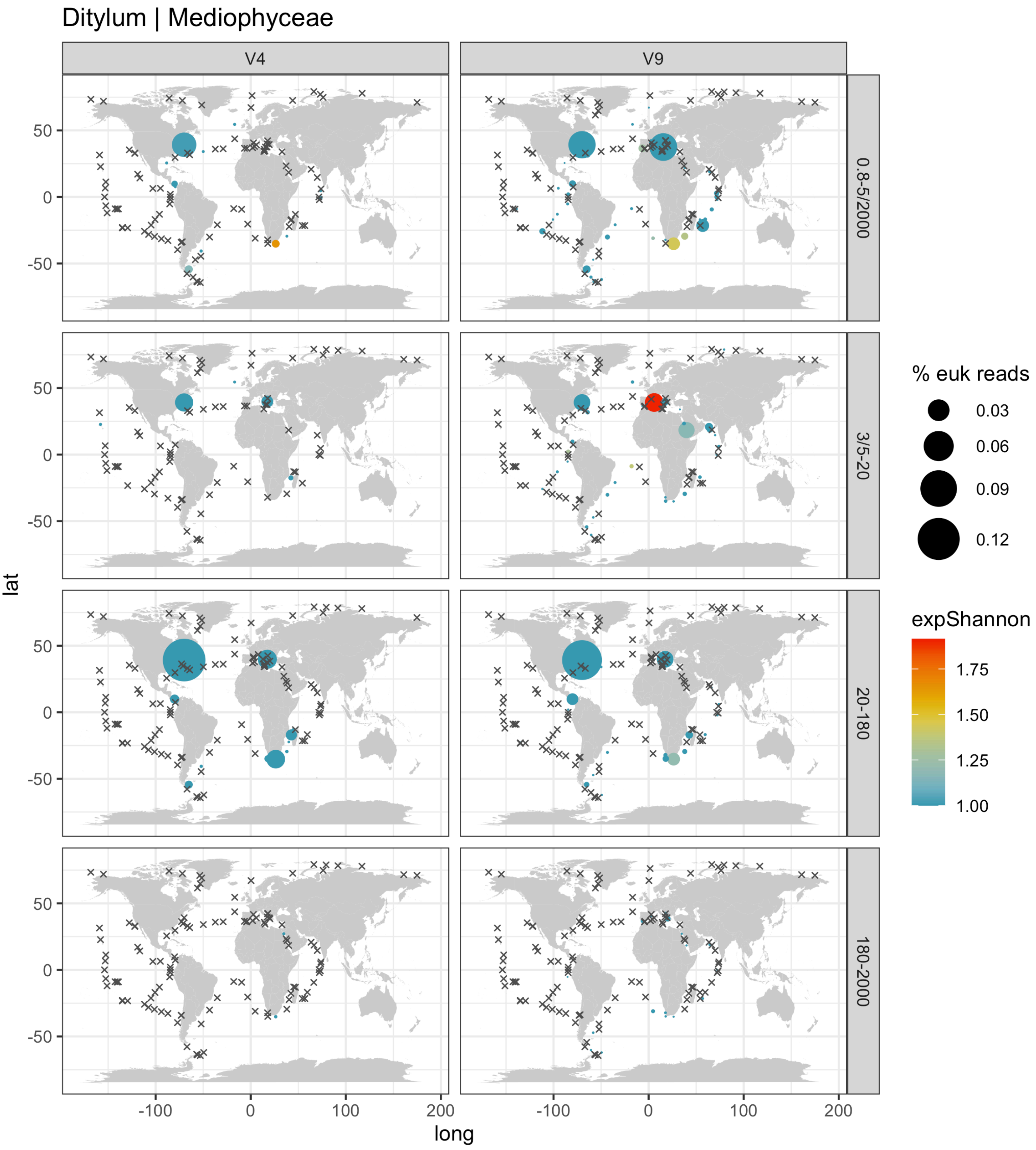

Ditylum | Mediophyceae
V4
V9
0.8-5/2000
3/5-20
20-180
180-2000
lat
long
50
0
-50
-100
100
200
% euk reads
0.03
0.06
0.09
0.12
expShannon
1.75
1.50
1.25
1.00

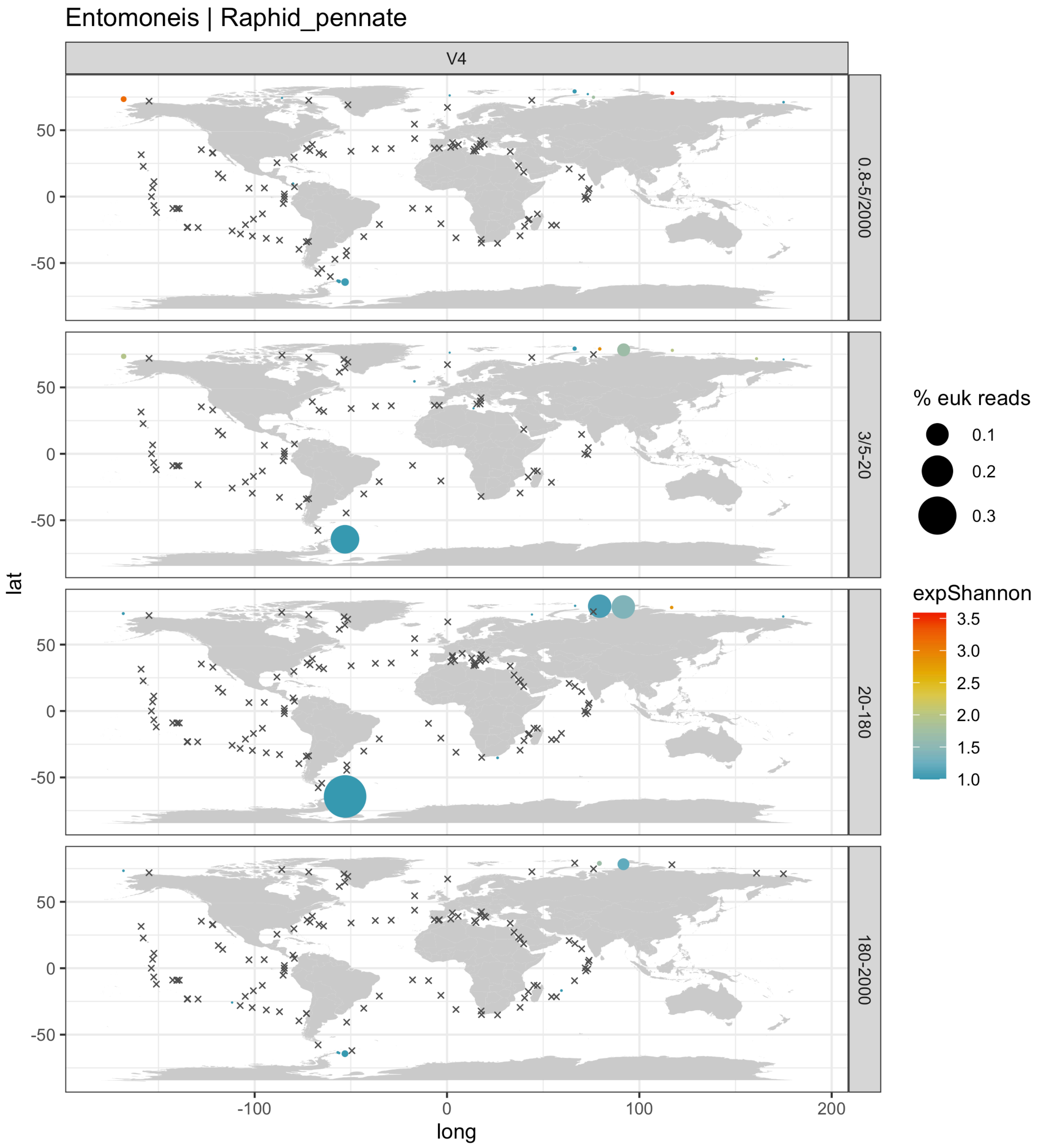

Entomoneis | Raphid_pennate
V4
0.8-5/2000
3/5-20
20-180
180-2000
lat
long
50
0
-50
-100
0
100
200
% euk reads
0.1
0.2
0.3
expShannon
3.5
3.0
2.5
2.0
1.5
1.0

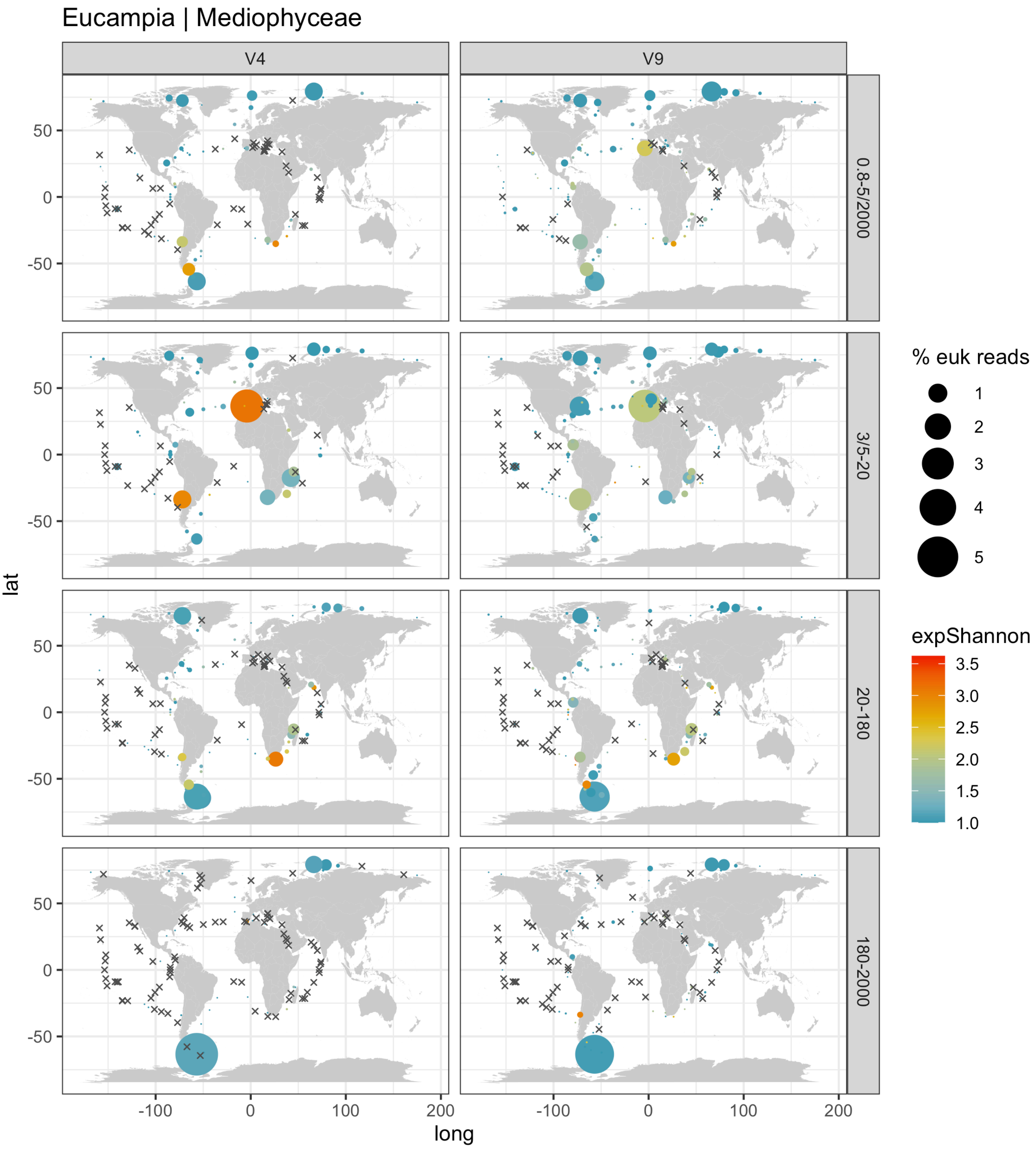

Eucampia | Mediophyceae
V4
V9
0.8-5/2000
3/5-20
20-180
180-2000
lat
long
50
0
-50
-100
0
100
200
% euk reads
1
2
3
4
5
expShannon
3.5
3.0
2.5
2.0
1.5
1.0

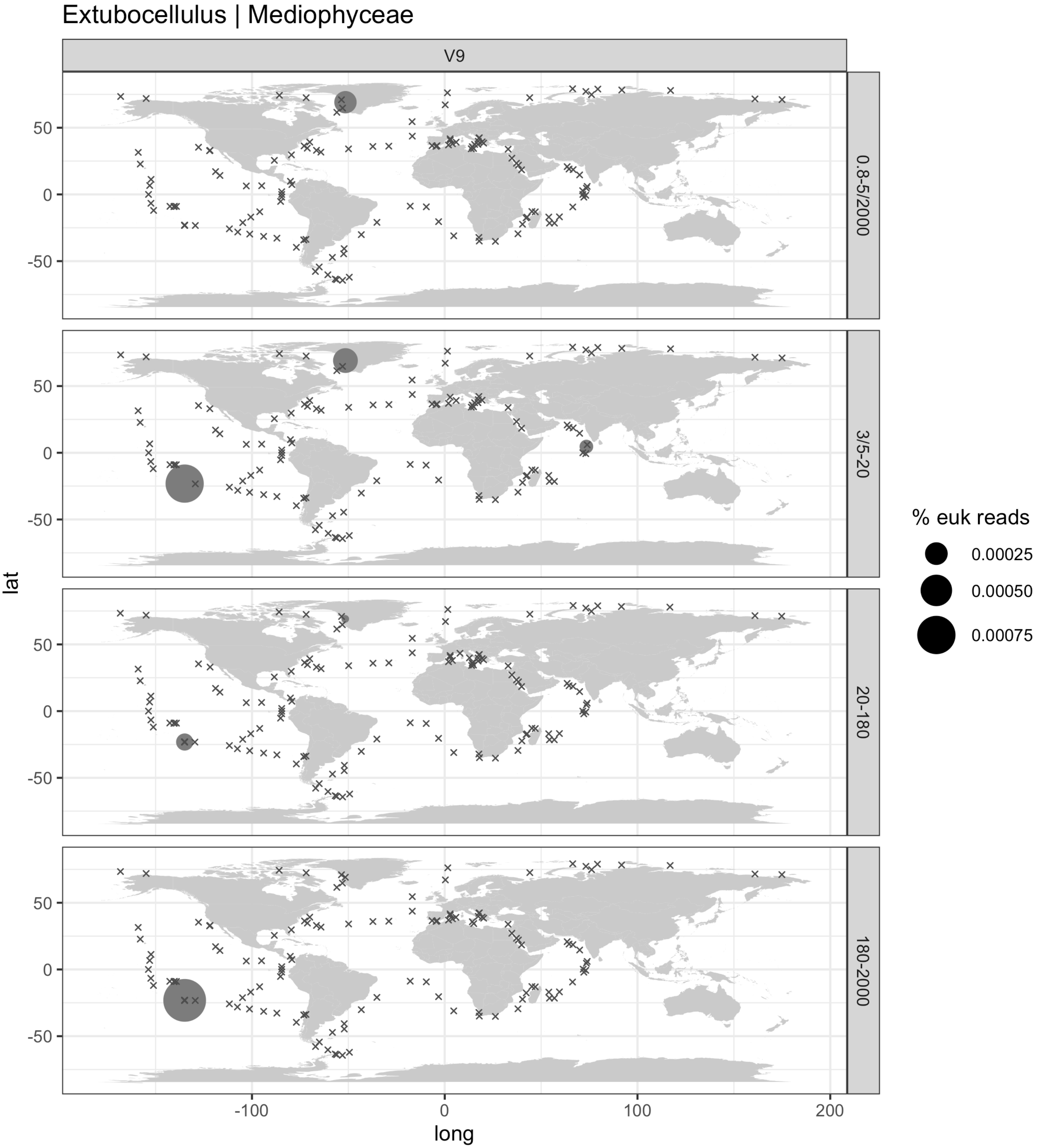
Extubocellulus | Mediophyceae
V9
0.8-5/2000
3/5-20
20-180
180-2000
lat
long
50
0
-50
-100
100
200
% euk reads
0.00025
0.00050
0.00075

Florella | Araphid_pennate

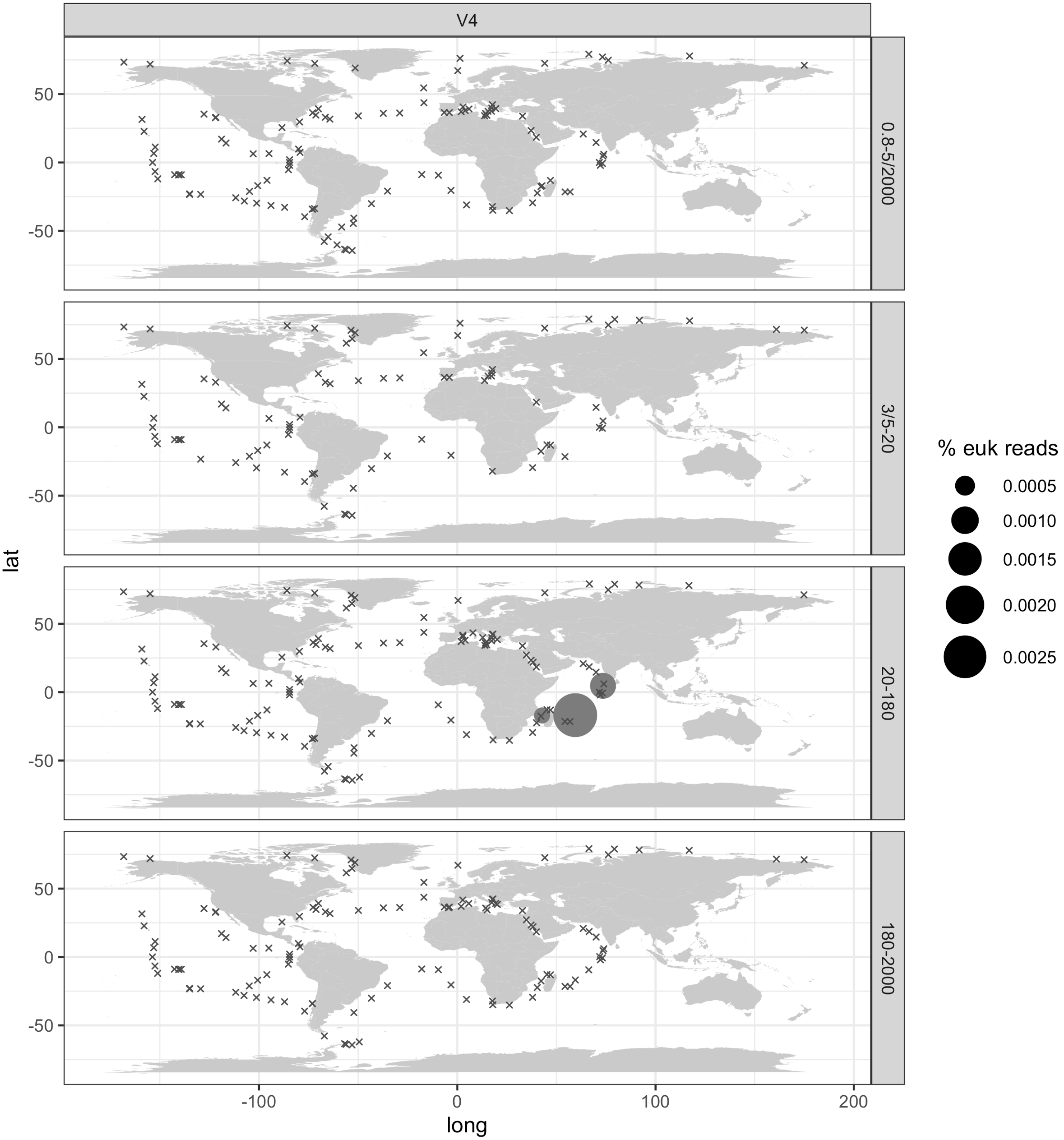

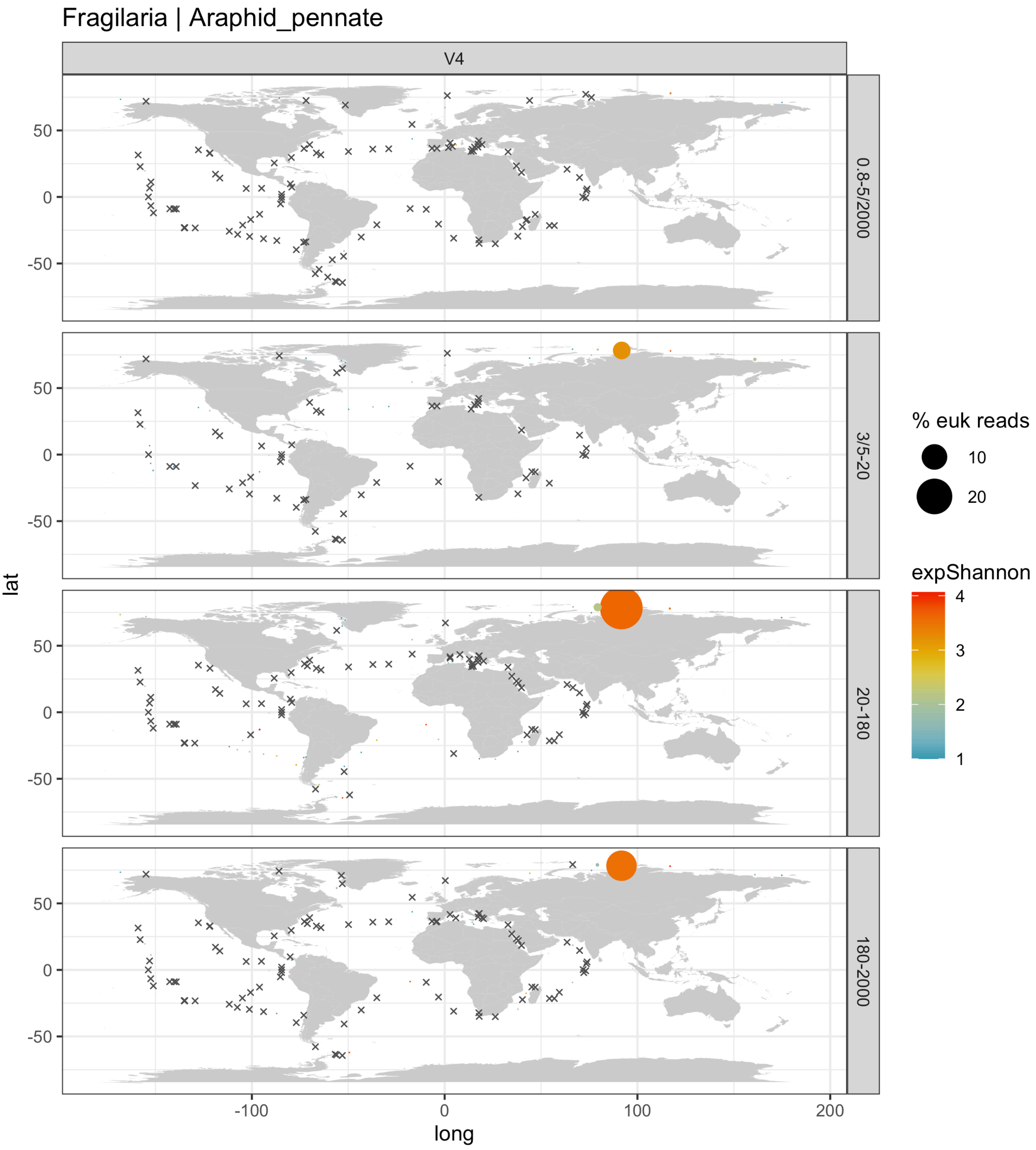

Fragilaria | Araphid_pennate
V4
0.8-5/2000
3/5-20
20-180
180-2000
lat
long
50
0
-50
-100
0
100
200
% euk reads
10
20
expShannon
4
3
2
1

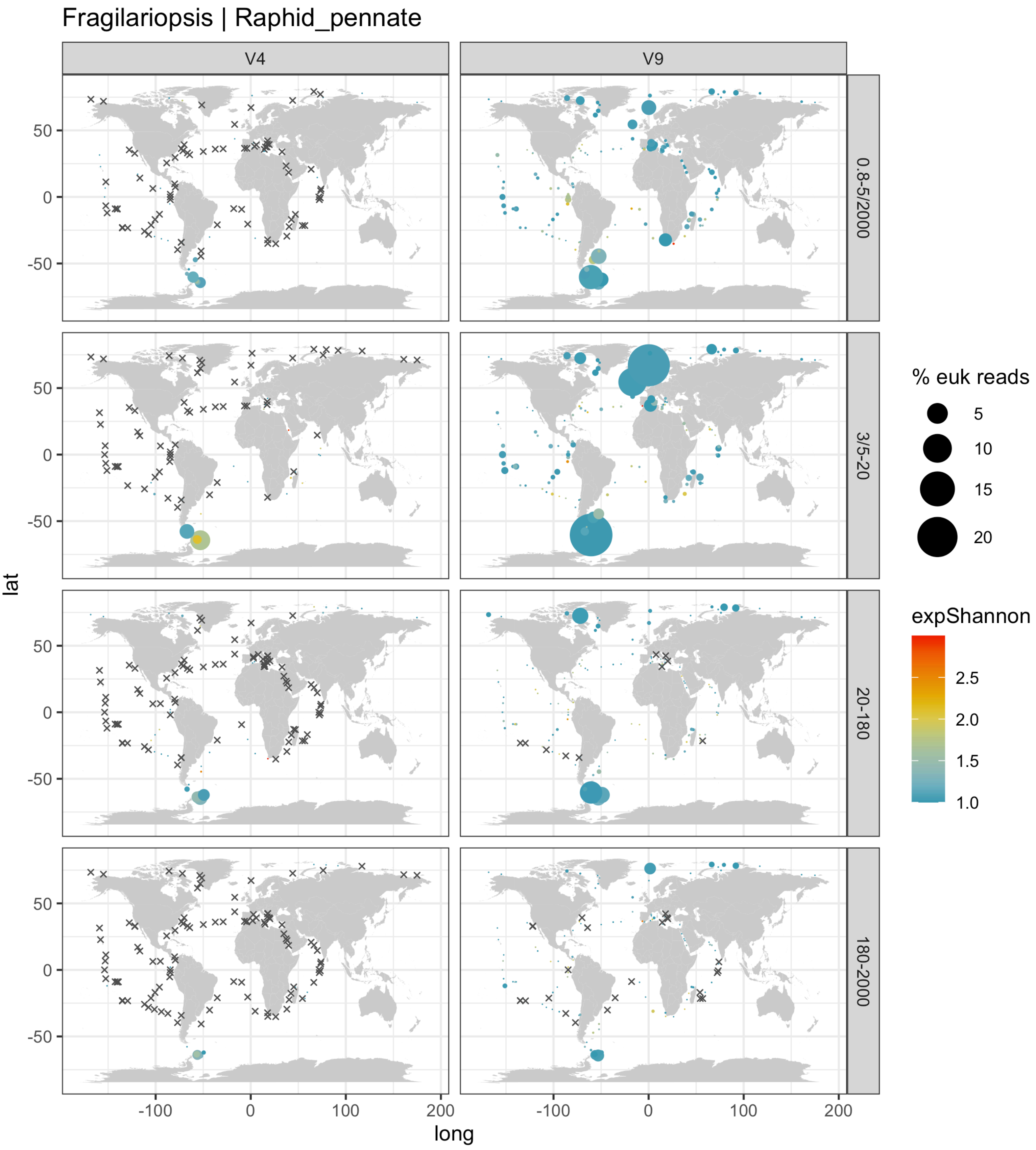
Fragilariopsis | Raphid_pennate
V4
V9
0.8-5/2000
3/5-20
20-180
180-2000
lat
long
50
0
-50
-100
100
200
% euk reads
5
10
15
20
expShannon
2.5
2.0
1.5
1.0

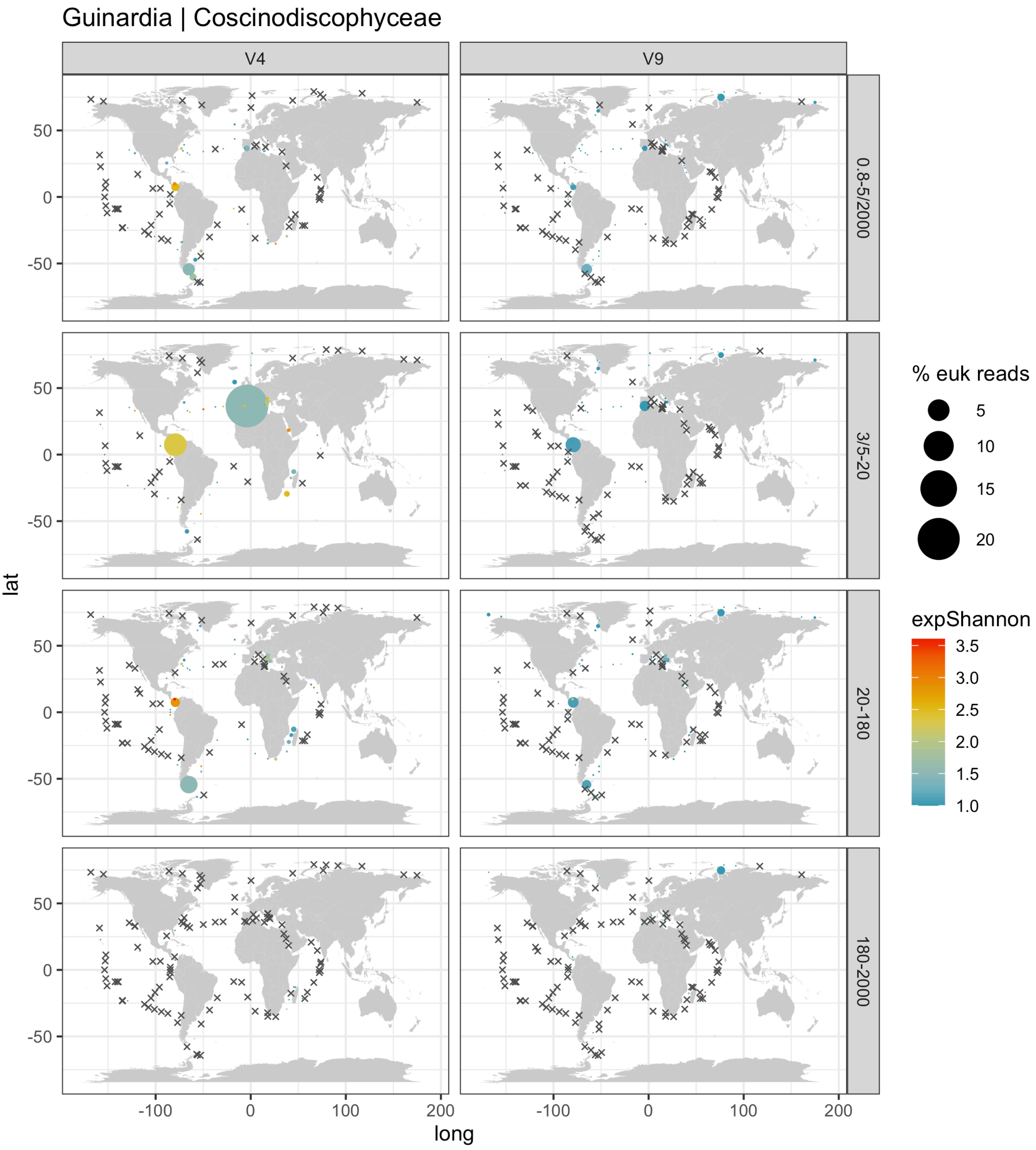
Guinardia | Coscinodiscophyceae
V4
V9
0.8-5/2000
3/5-20
20-180
180-2000
lat
long
50
0
-50
-100
0
100
200
% euk reads
5
10
15
20
expShannon
3.5
3.0
2.5
2.0
1.5
1.0

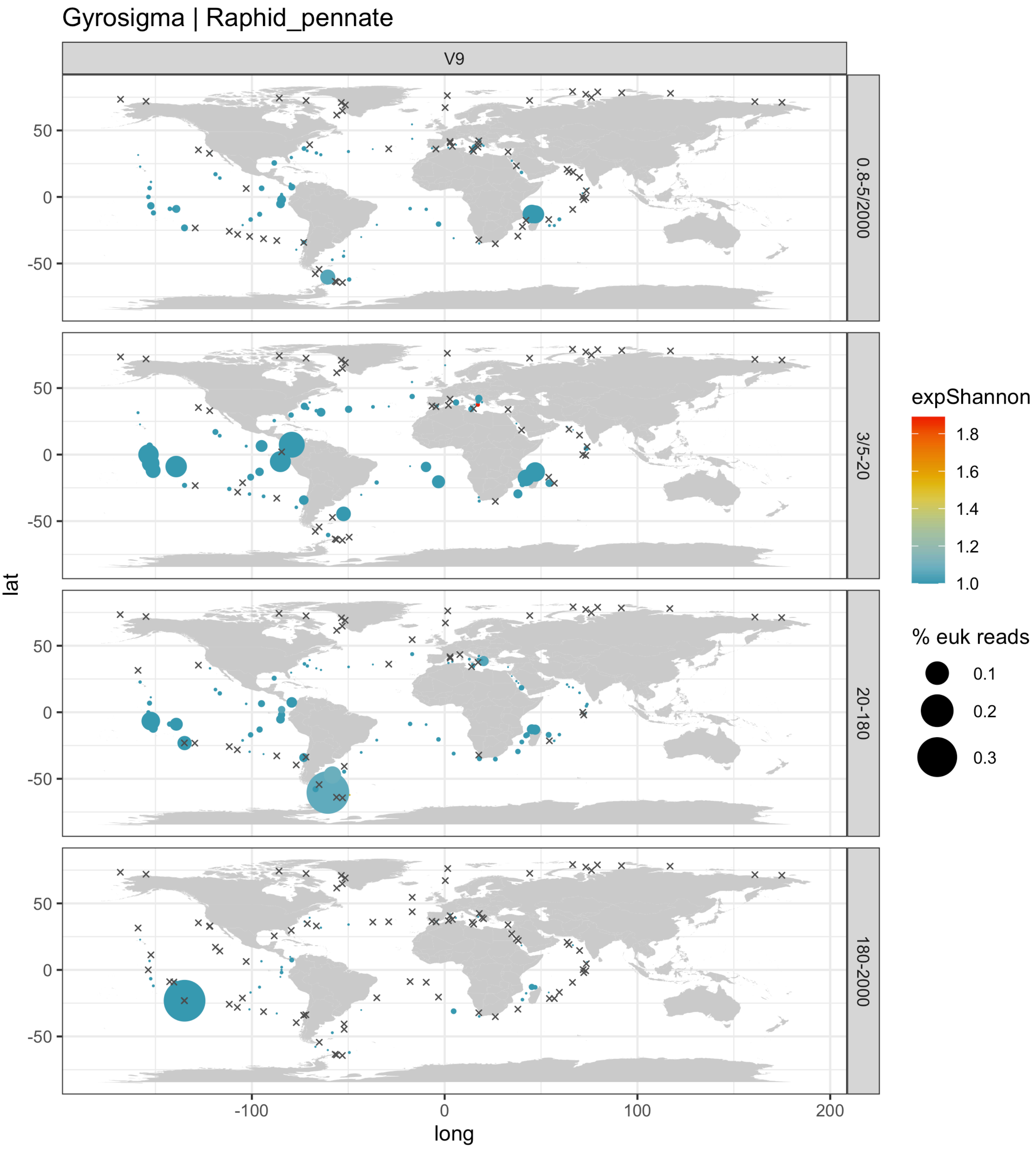

Gyrosigma | Raphid_pennate
V9
0.8-5/2000
3/5-20
20-180
180-2000
lat
long
50
0
-50
-100
100
200
expShannon
1.8
1.6
1.4
1.2
1.0
% euk reads
0.1
0.2
0.3

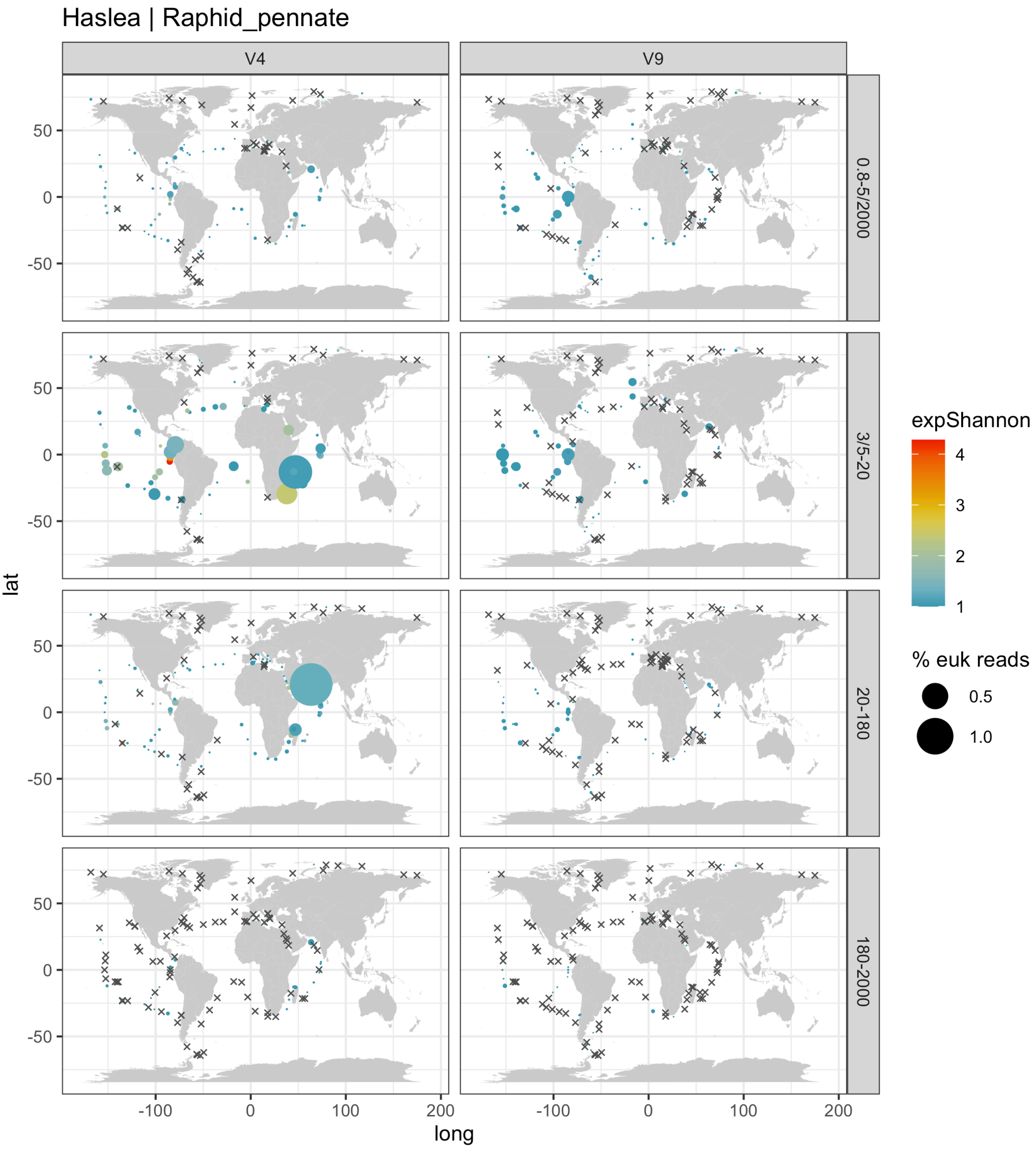

Haslea | Raphid_pennate
V4
V9
0.8-5/2000
3/5-20
20-180
180-2000
lat
long
50
0
-50
-100
0
100
200
expShannon
4
3
2
1
% euk reads
0.5
1.0

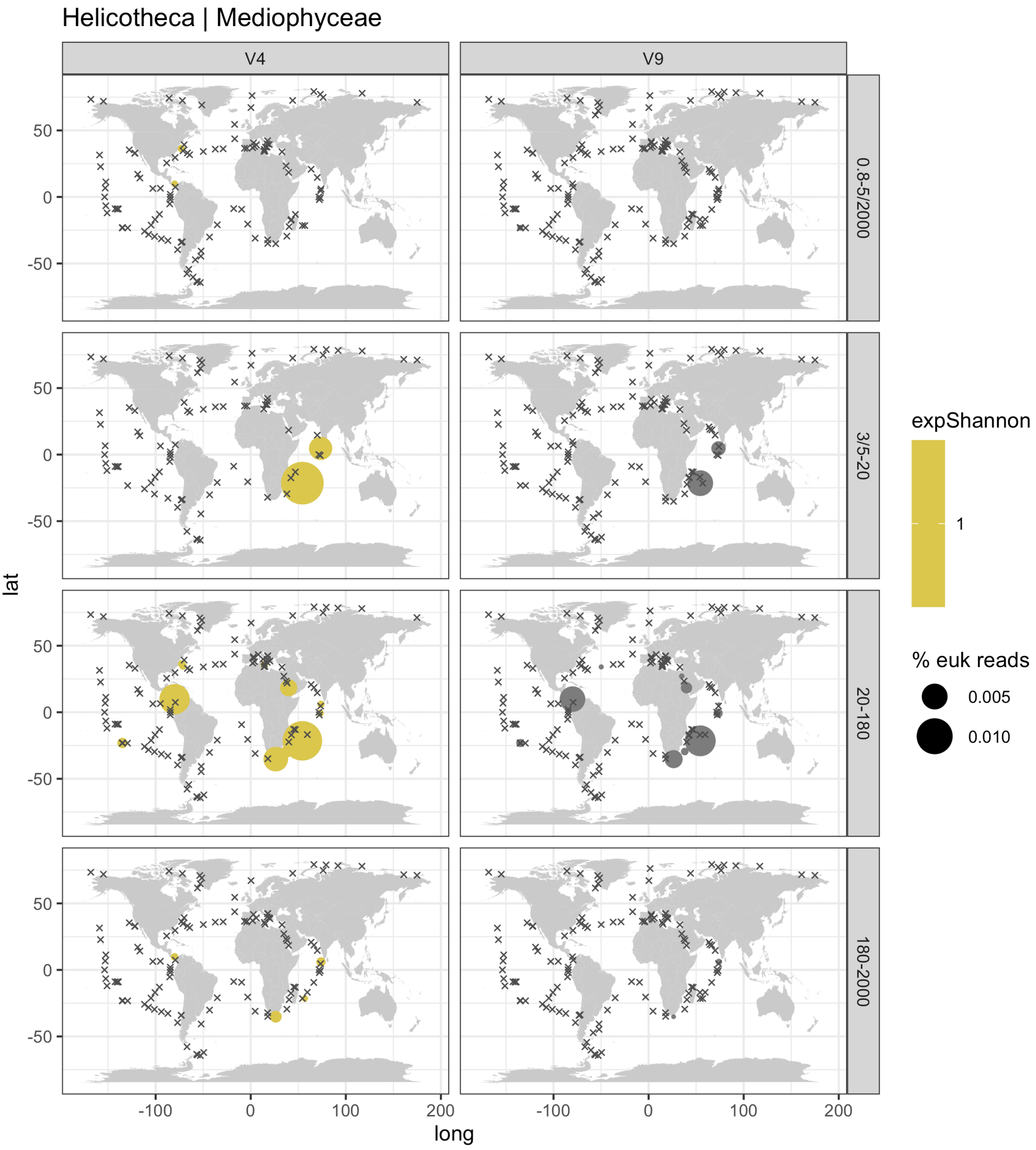

Helicotheca | Mediophyceae
V4
V9
0.8-5/2000
3/5-20
20-180
180-2000
lat
long
50
0
-50
-100
0
100
200
expShannon
1
% euk reads
0.005
0.010

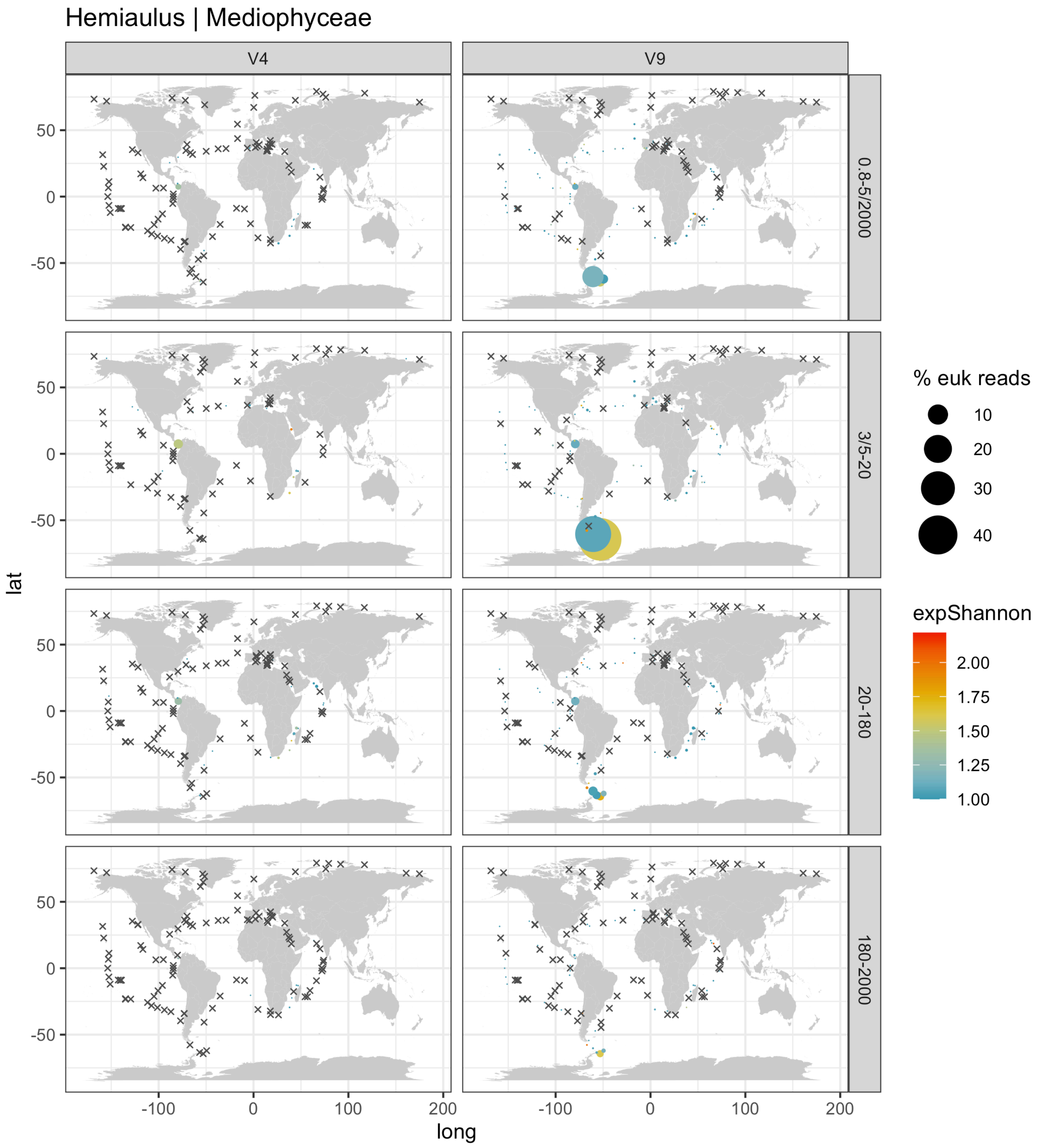

Hemiaulus | Mediophyceae
V4
V9
0.8-5/2000
3/5-20
20-180
180-2000
lat
long
50
0
-50
-100
0
100
200
% euk reads
10
20
30
40
expShannon
2.00
1.75
1.50
1.25
1.00

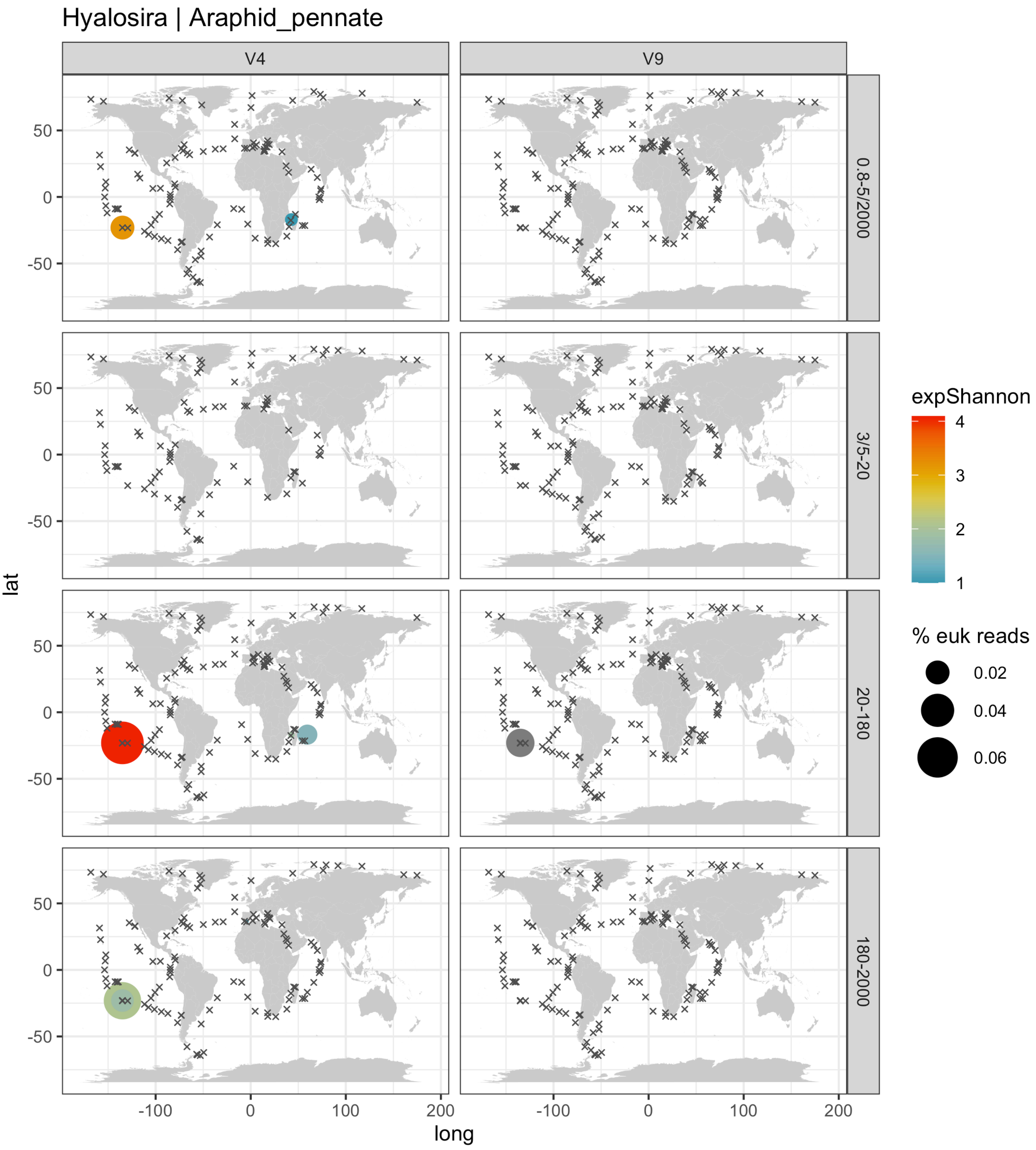

Hyalosira | Araphid_pennate
V4
V9
0.8-5/2000
3/5-20
20-180
180-2000
lat
long
50
0
-50
-100
100
200
expShannon
4
3
2
1
% euk reads
0.02
0.04
0.06

Hyalosynedra | Araphid_pennate

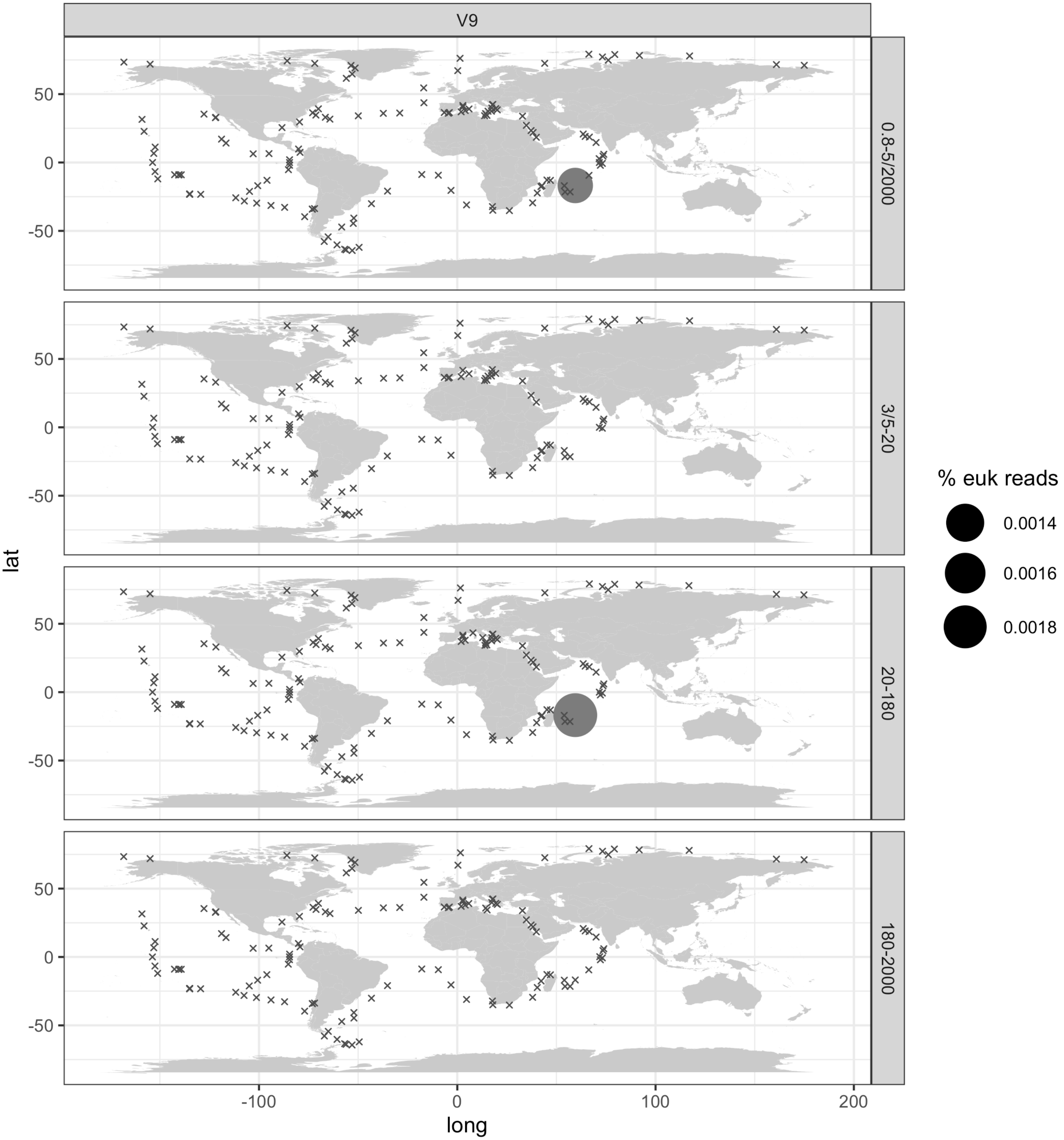

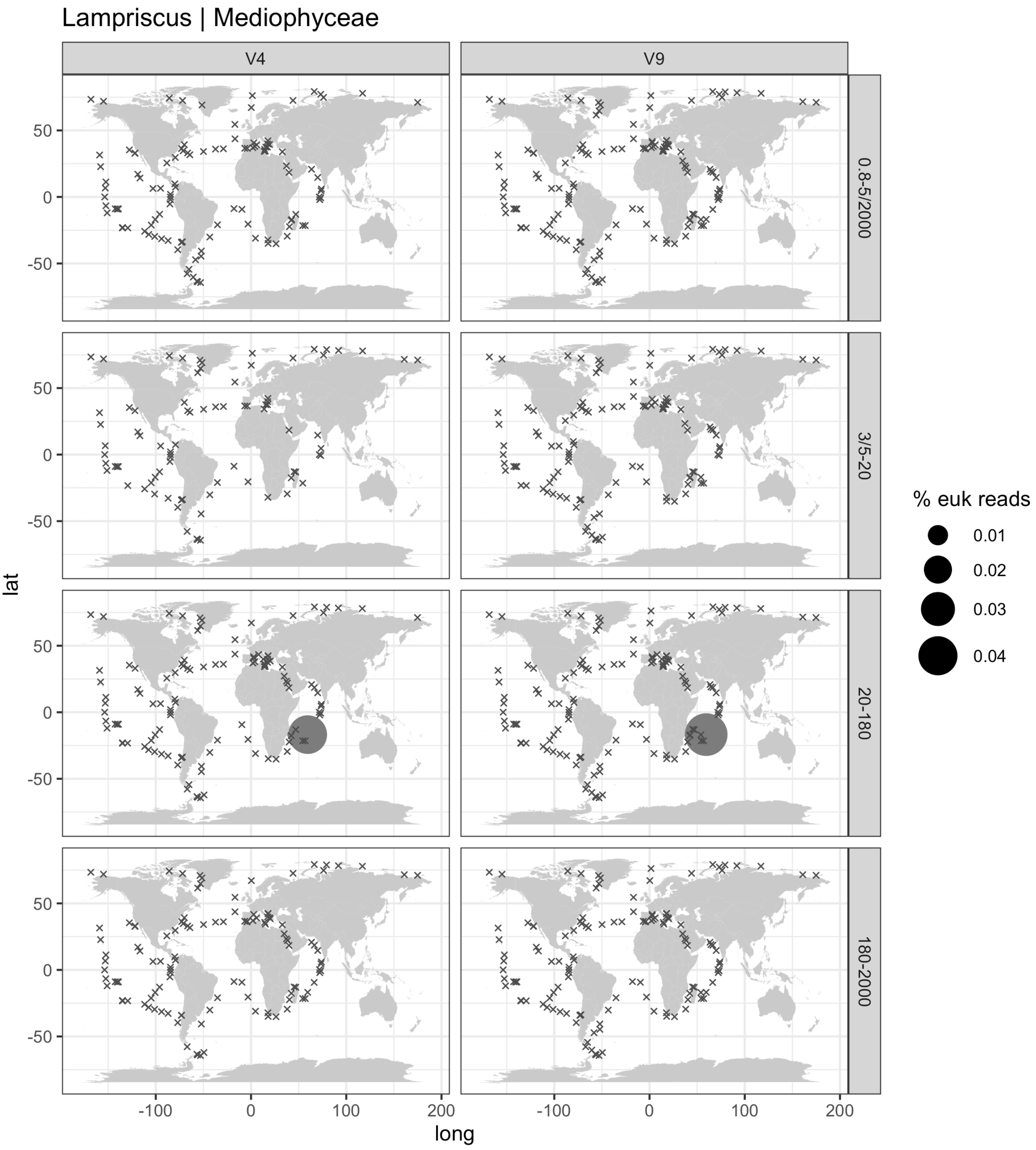

Lampriscus | Mediophyceae
V4
V9
0.8-5/2000
3/5-20
20-180
180-2000
lat
long
50
0
-50
-100
100
200
% euk reads
0.01
0.02
0.03
0.04

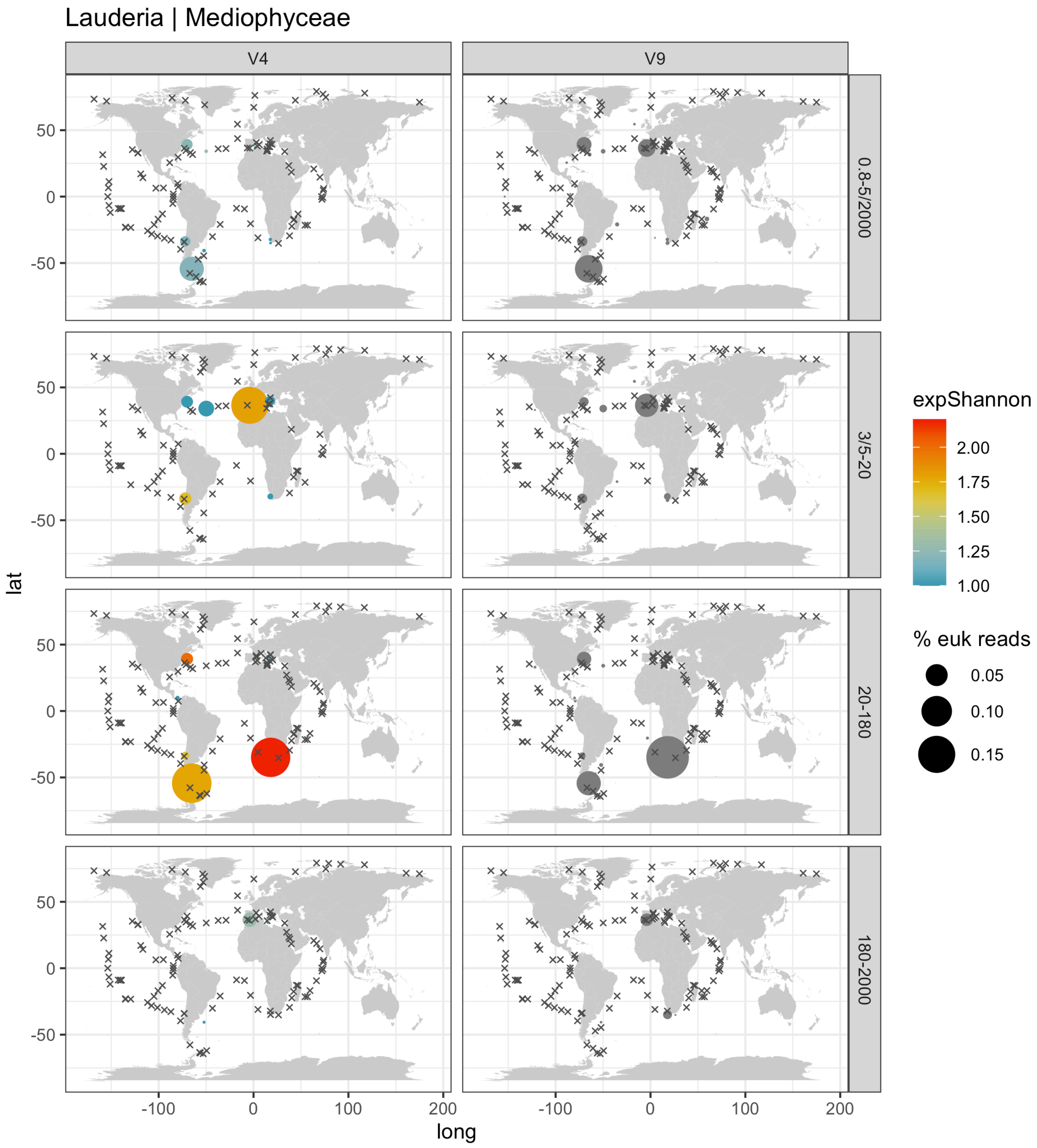
Lauderia | Mediophyceae
V4
V9
0.8-5/2000
3/5-20
20-180
180-2000
lat
long
50
0
-50
-100
0
100
200
expShannon
2.00
1.75
1.50
1.25
1.00
% euk reads
0.05
0.10
0.15

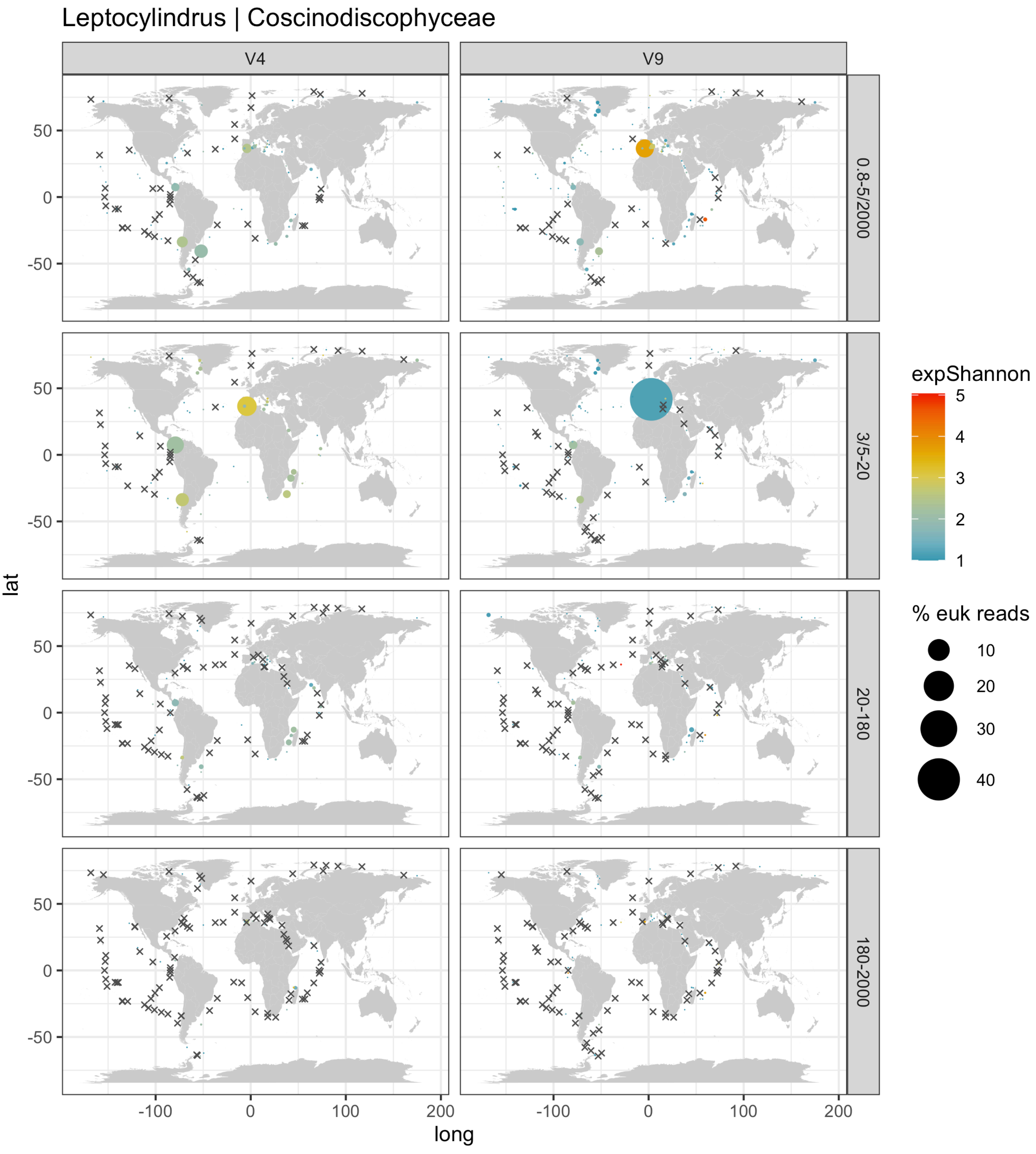
Leptocylindrus | Coscinodiscophyceae
V4
V9
0.8-5/2000
3/5-20
20-180
180-2000
50
0
-50
-100
0
100
200
lat
long
expShannon
5
4
3
2
1
% euk reads
10
20
30
40

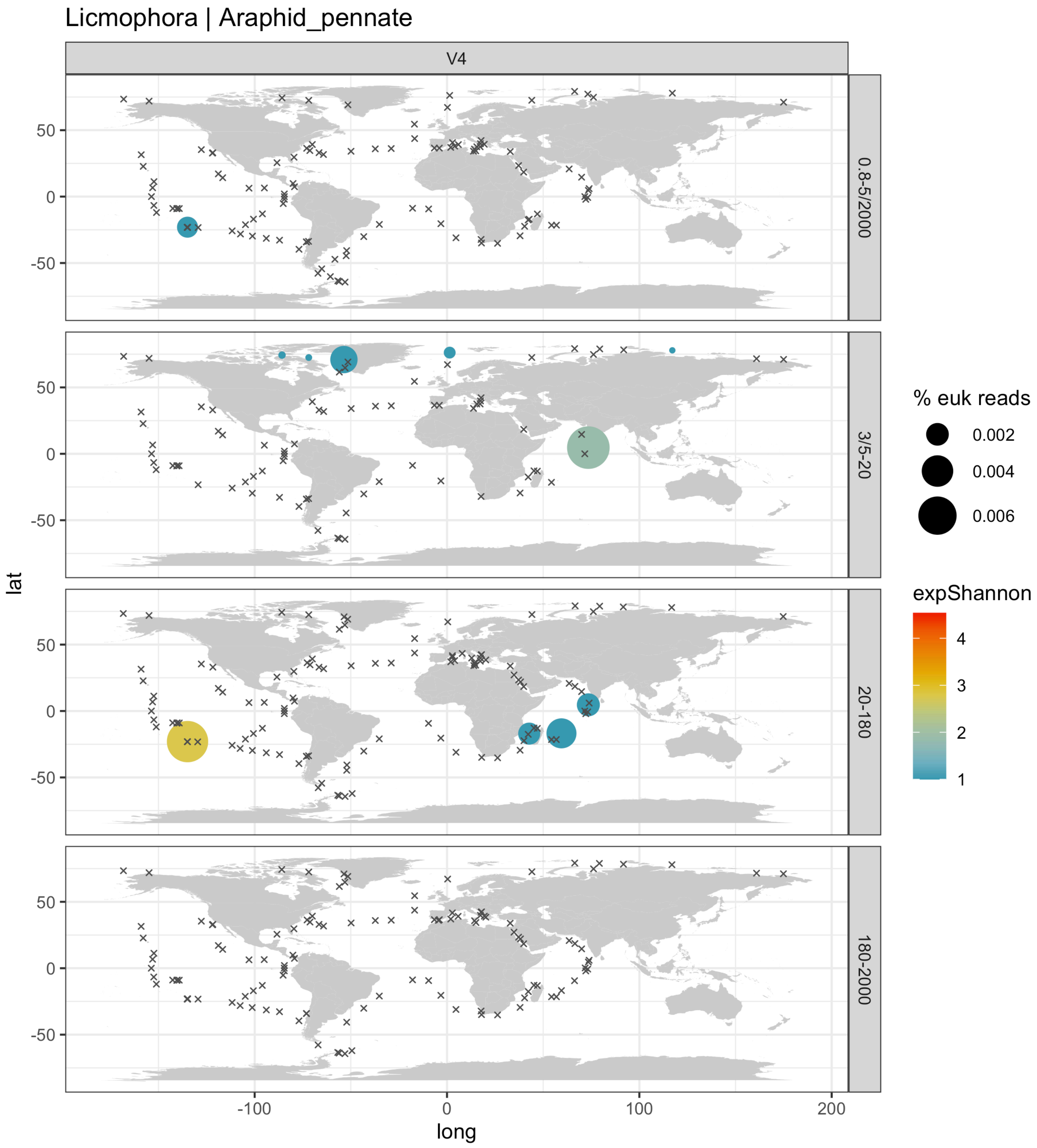
Licmophora | Araphid_pennate
V4
0.8-5/2000
3/5-20
20-180
180-2000
lat
long
-100
0
100
200
50
0
-50
% euk reads
0.002
0.004
0.006
expShannon
4
3
2
1

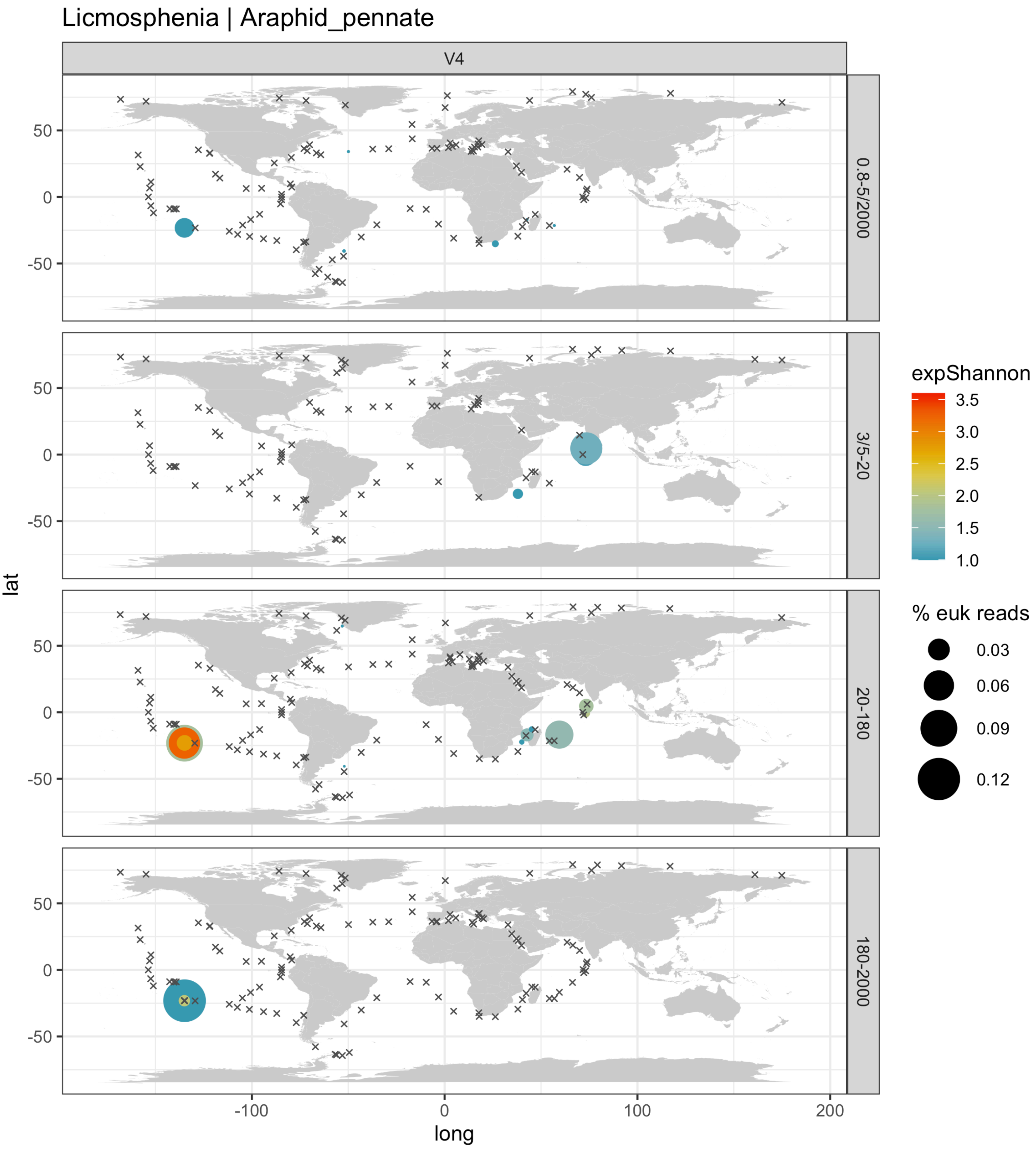

Licmosphenia | Araphid_pennate
V4
0.8-5/2000
3/5-20
20-180
180-2000
lat
long
50
0
-50
-100
100
200
expShannon
3.5
3.0
2.5
2.0
1.5
1.0
% euk reads
0.03
0.06
0.09
0.12

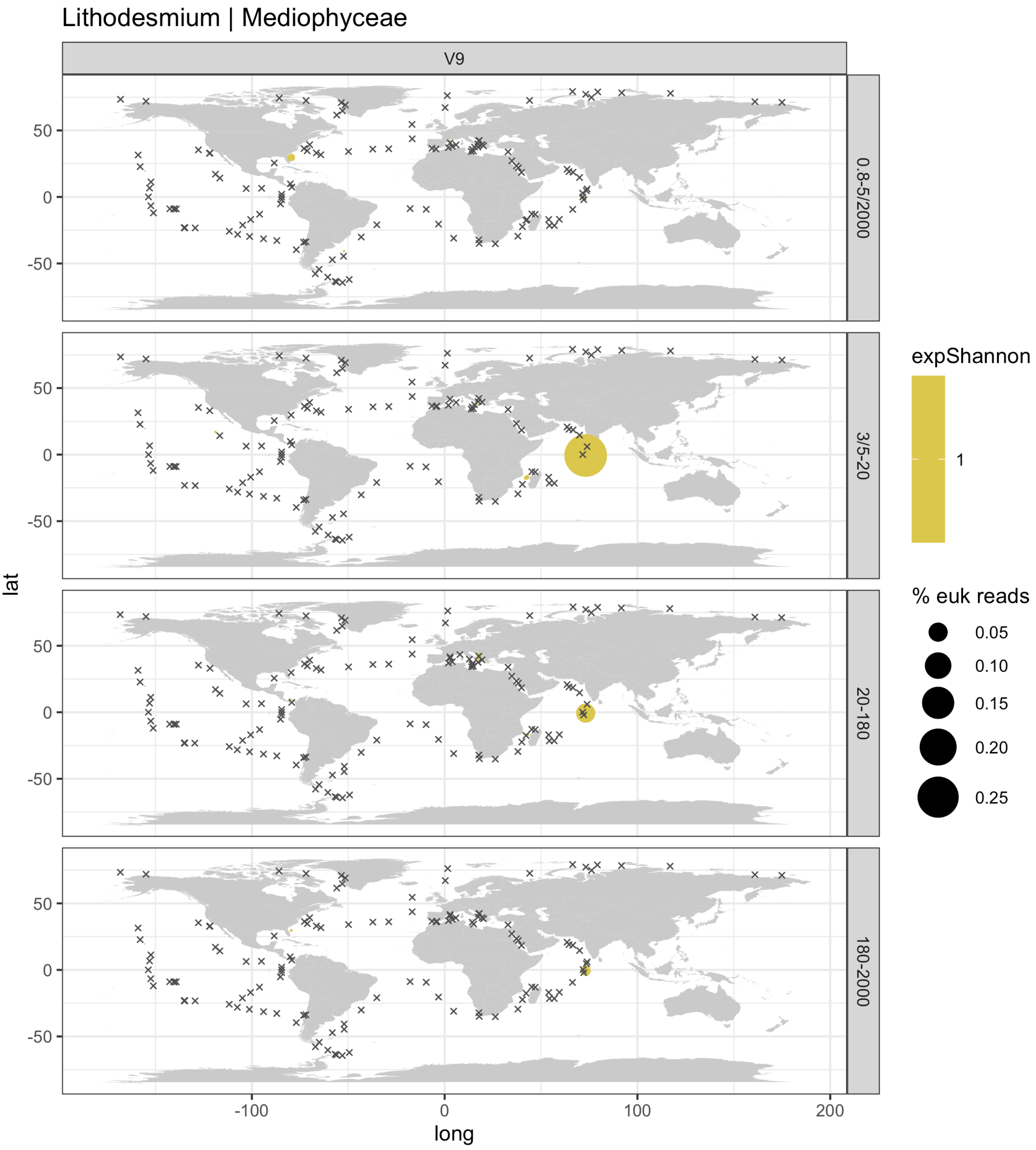

Lithodesmium | Mediophyceae
V9
0.8-5/2000
3/5-20
20-180
180-2000
lat
long
50
0
-50
-100
0
100
200
expShannon
1
% euk reads
0.05
0.10
0.15
0.20
0.25

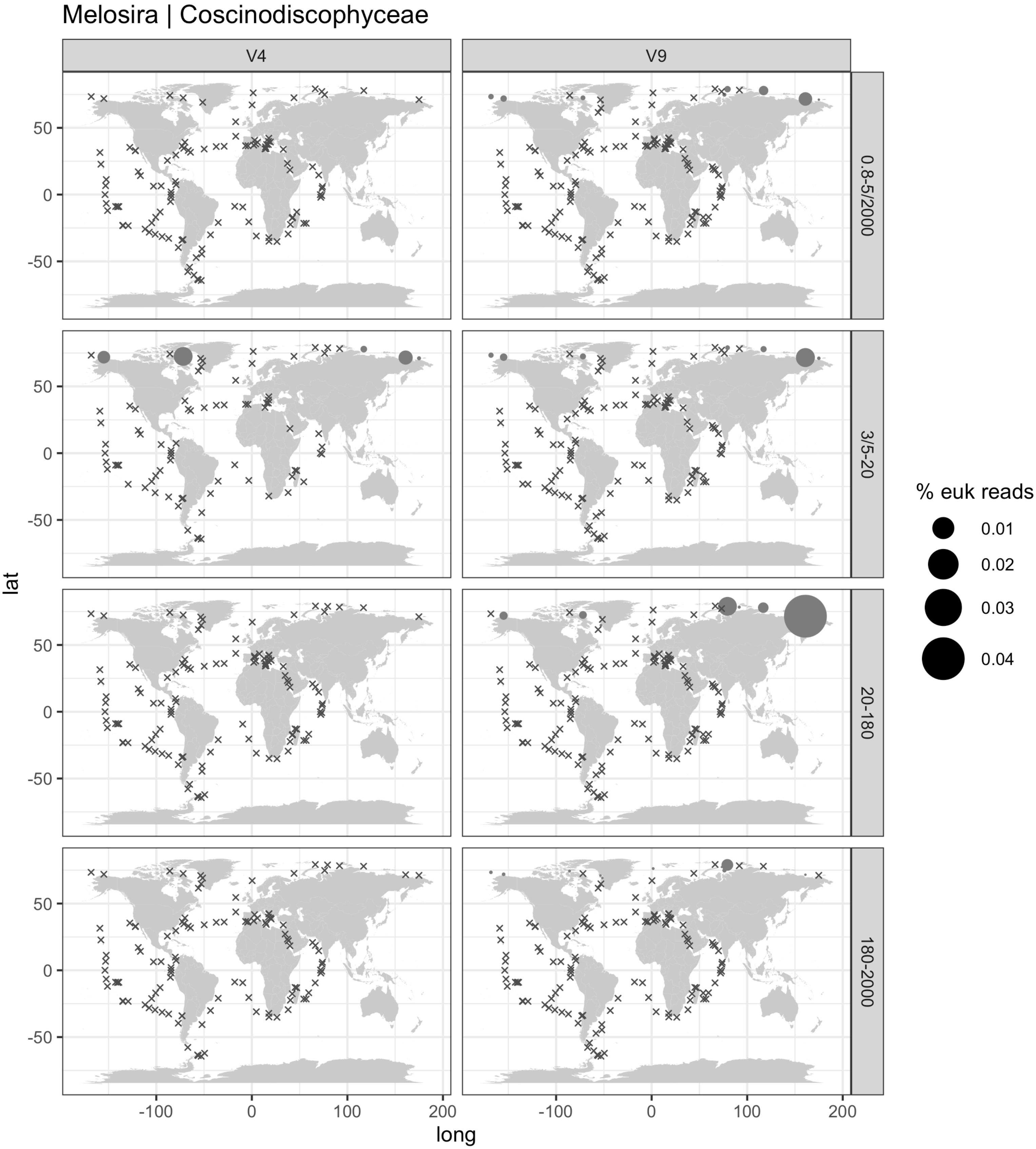

Melosira | Coscinodiscophyceae
V4
V9
0.8-5/2000
3/5-20
20-180
180-2000
lat
long
50
0
-50
-100
100
200
% euk reads
0.01
0.02
0.03
0.04

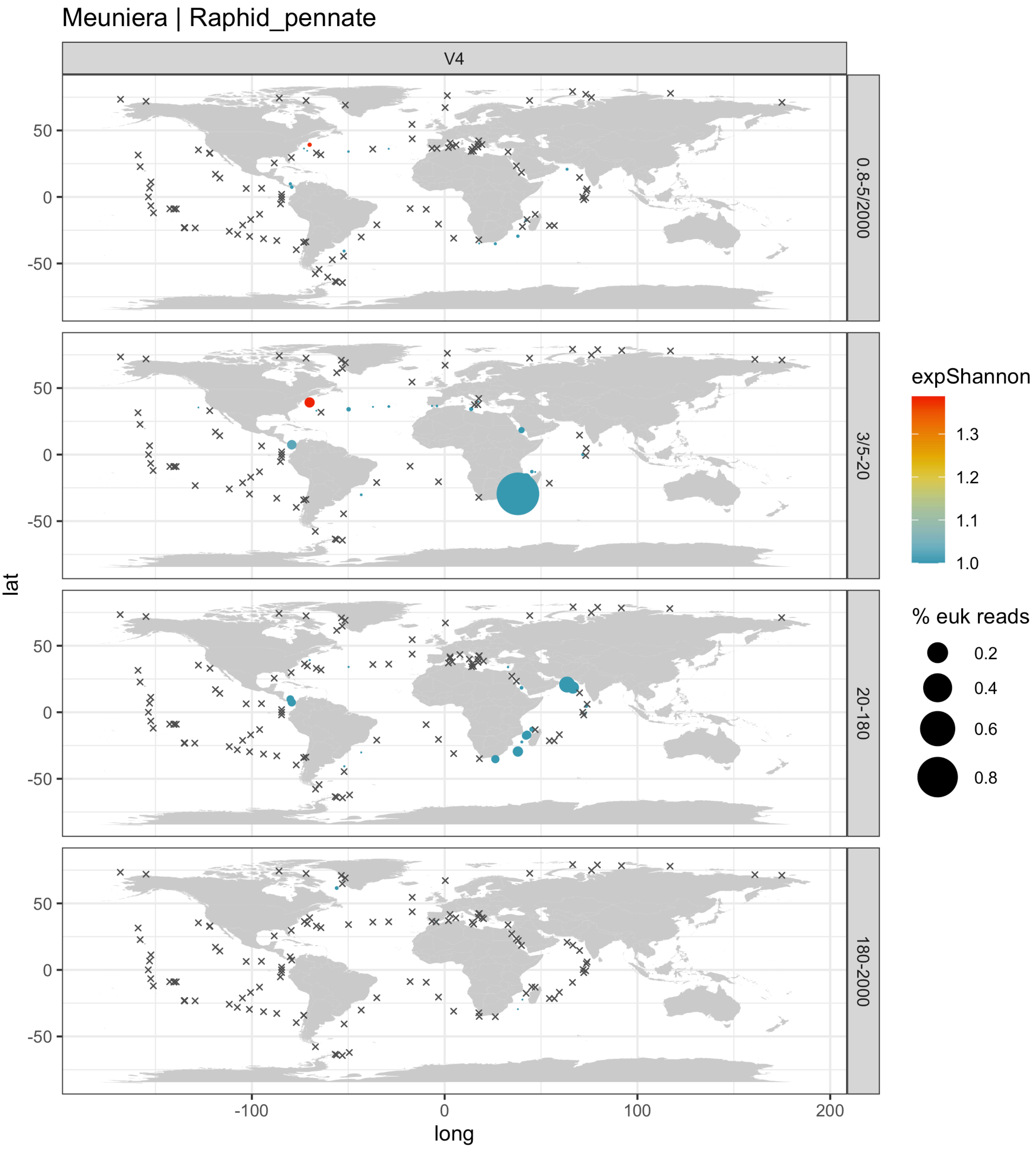

Meuniera | Raphid_pennate
V4
0.8-5/2000
3/5-20
20-180
180-2000
lat
long
50
0
-50
-100
0
100
200
expShannon
1.3
1.2
1.1
1.0
% euk reads
0.2
0.4
0.6
0.8

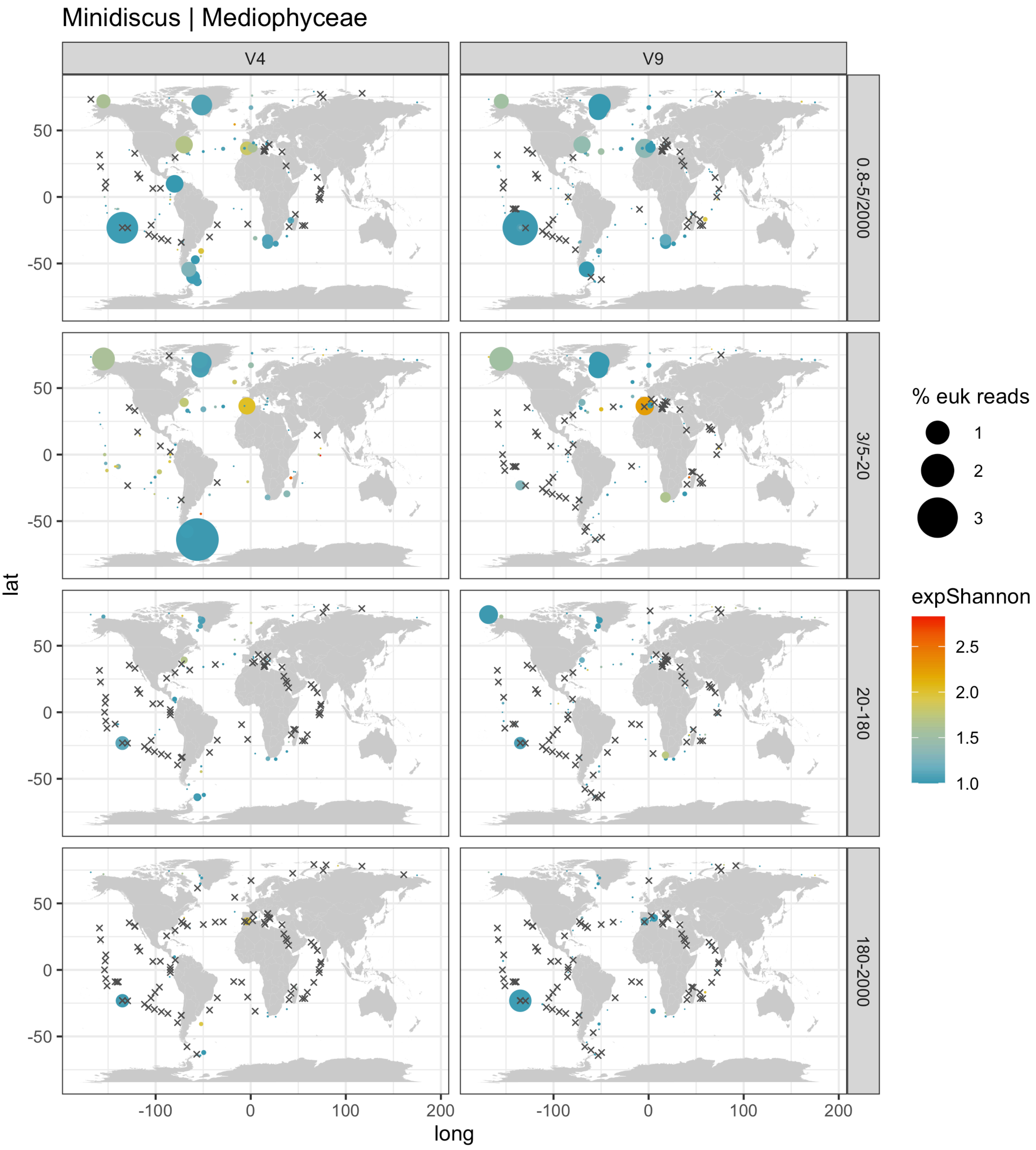
Minidiscus | Mediophyceae
V4
V9
0.8-5/2000
3/5-20
20-180
180-2000
lat
long
50
0
-50
-100
100
200
% euk reads
1
2
3
expShannon
2.5
2.0
1.5
1.0

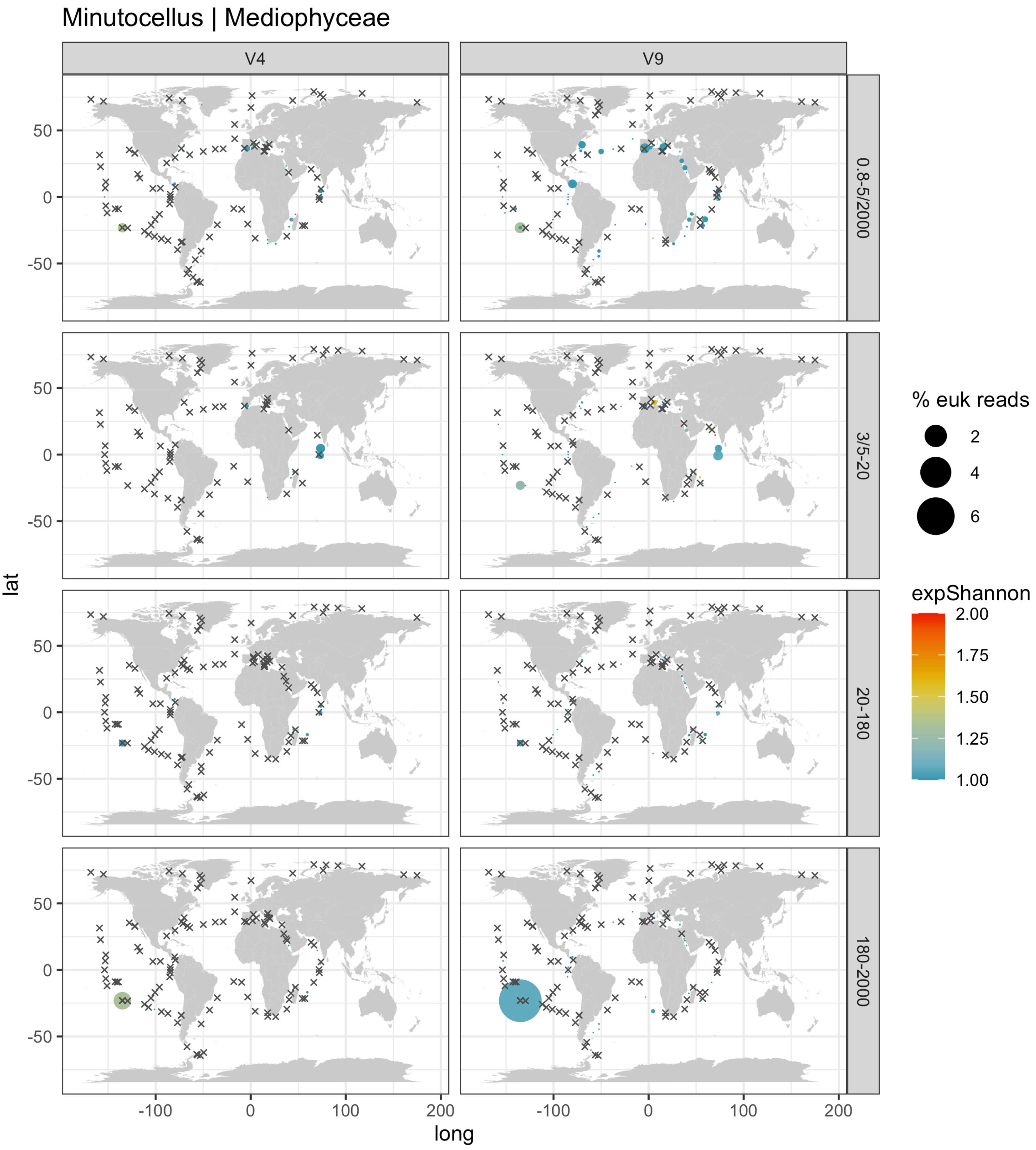

Minutocellus | Mediophyceae
V4
V9
0.8-5/2000
3/5-20
20-180
180-2000
lat
long
50
0
-50
-100
0
100
200
% euk reads
2
4
6
expShannon
2.00
1.75
1.50
1.25
1.00

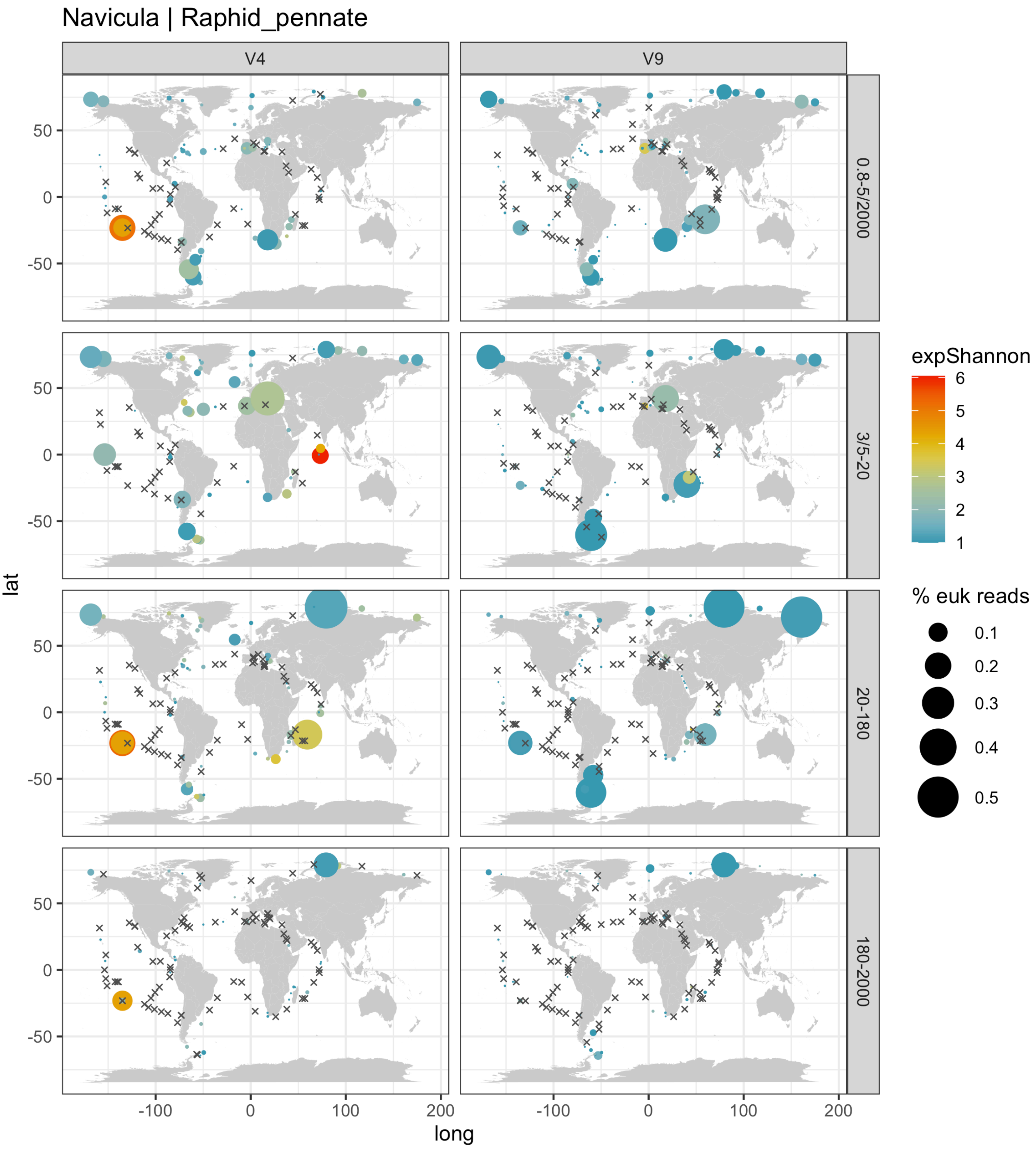

Navicula | Raphid_pennate
V4
V9
0.8-5/2000
3/5-20
20-180
180-2000
lat
long
50
0
-50
-100
0
100
200
expShannon
6
5
4
3
2
1
% euk reads
0.1
0.2
0.3
0.4
0.5

Nitzschia | Raphid_pennate

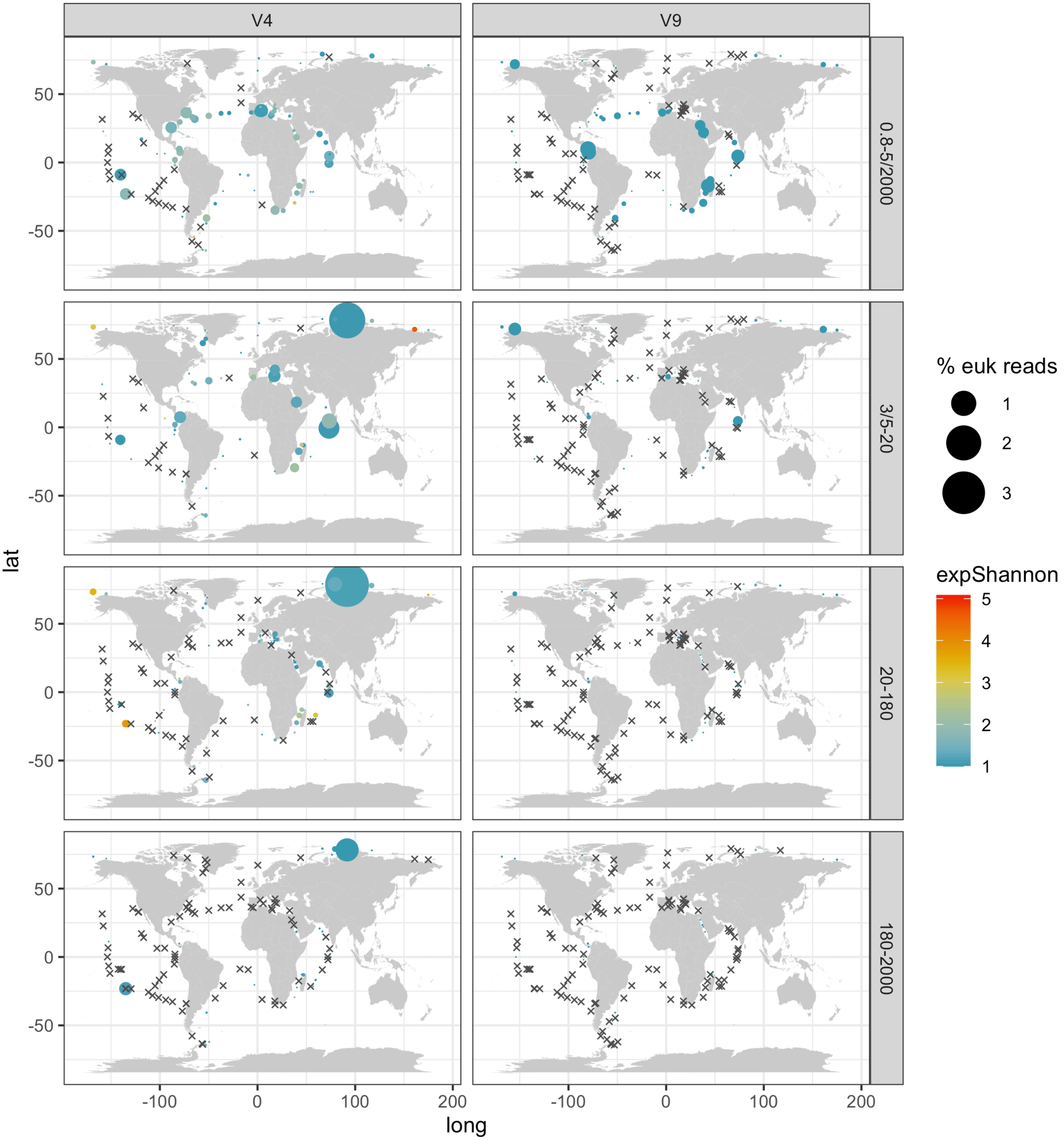

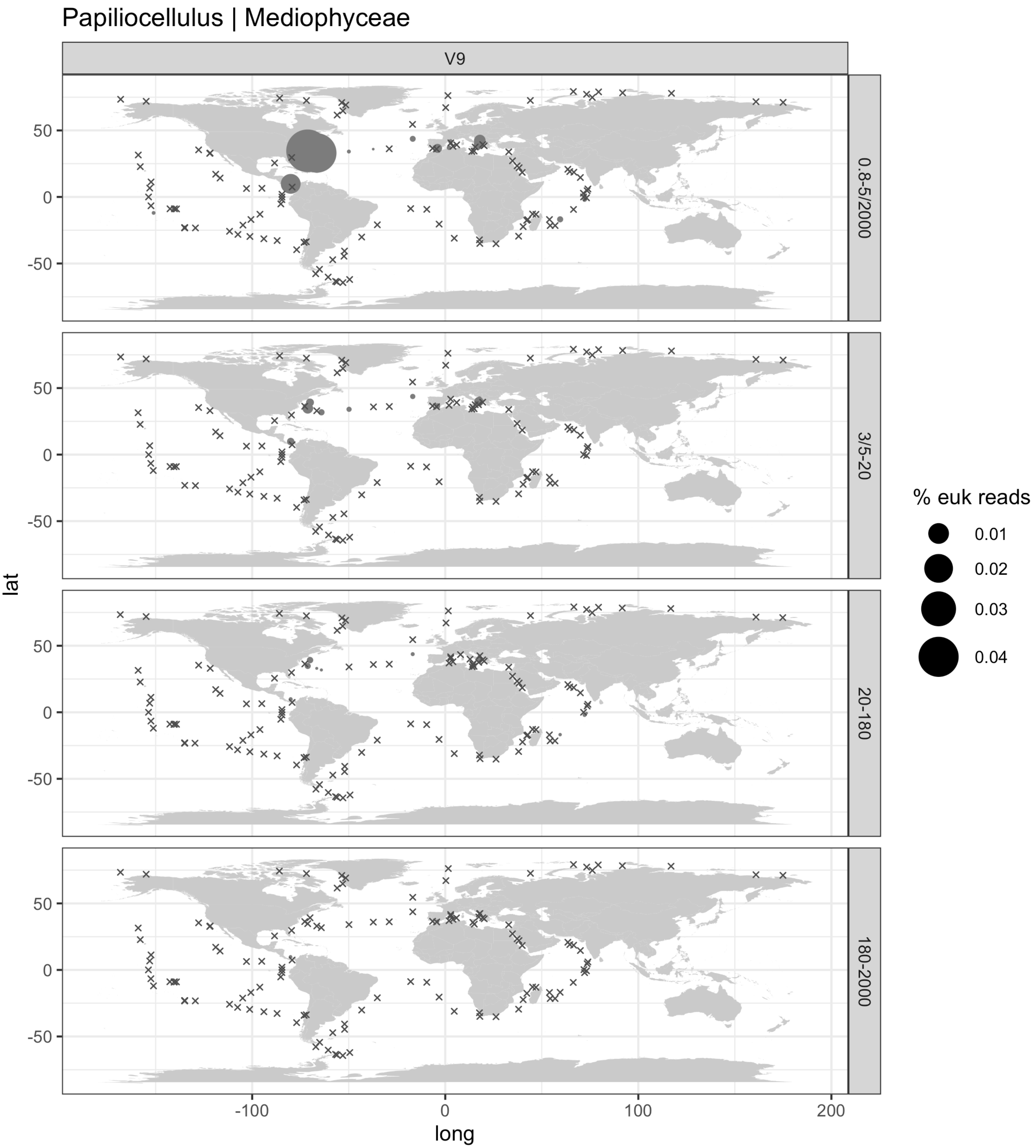

Papiliocellulus | Mediophyceae
V9
0.8-5/2000
3/5-20
20-180
180-2000
lat
long
50
0
-50
-100
0
100
200
% euk reads
0.01
0.02
0.03
0.04

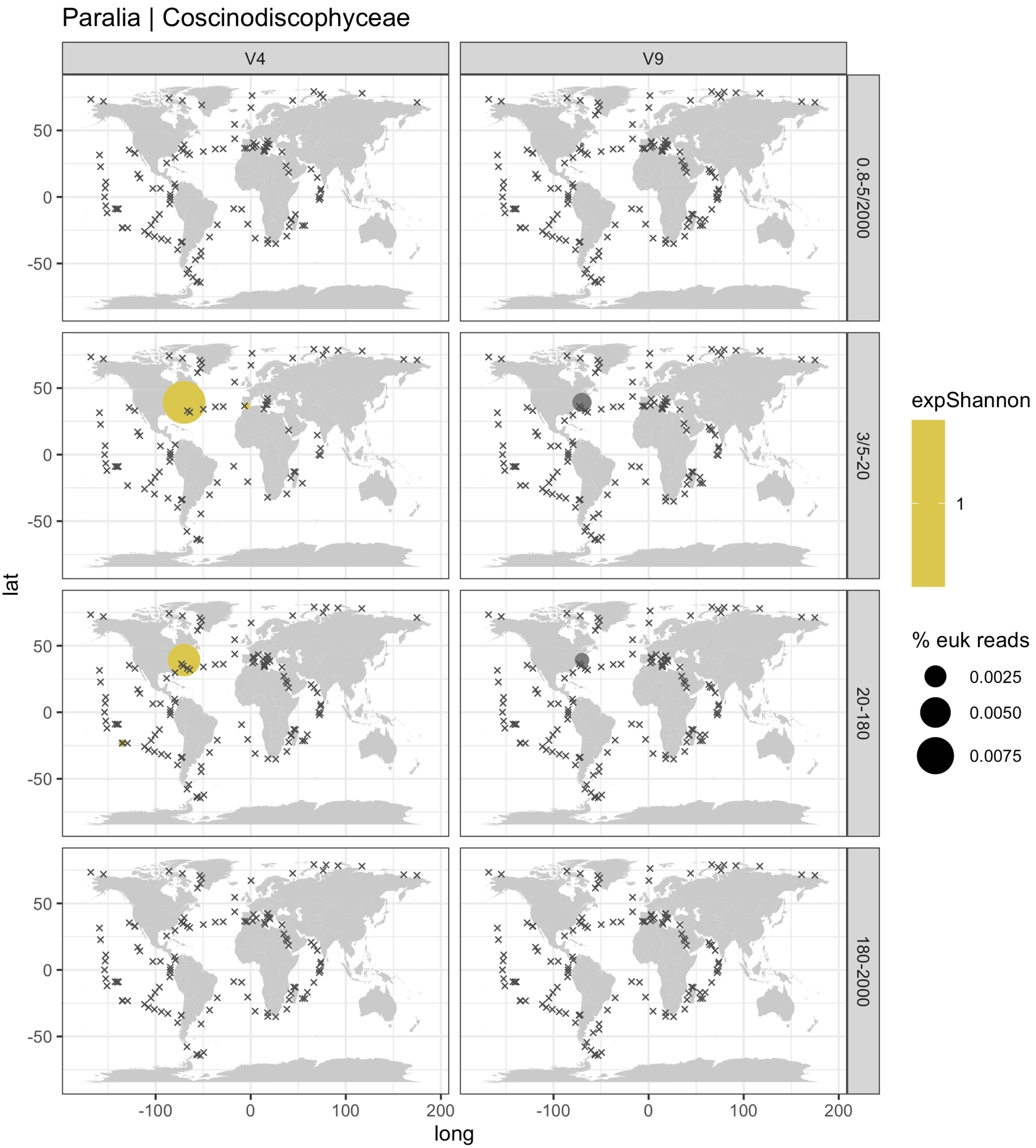
Paralia | Coscinodiscophyceae
V4
V9
0.8-5/2000
3/5-20
20-180
180-2000
lat
long
50
0
-50
-100
0
100
200
expShannon
1
% euk reads
0.0025
0.0050
0.0075

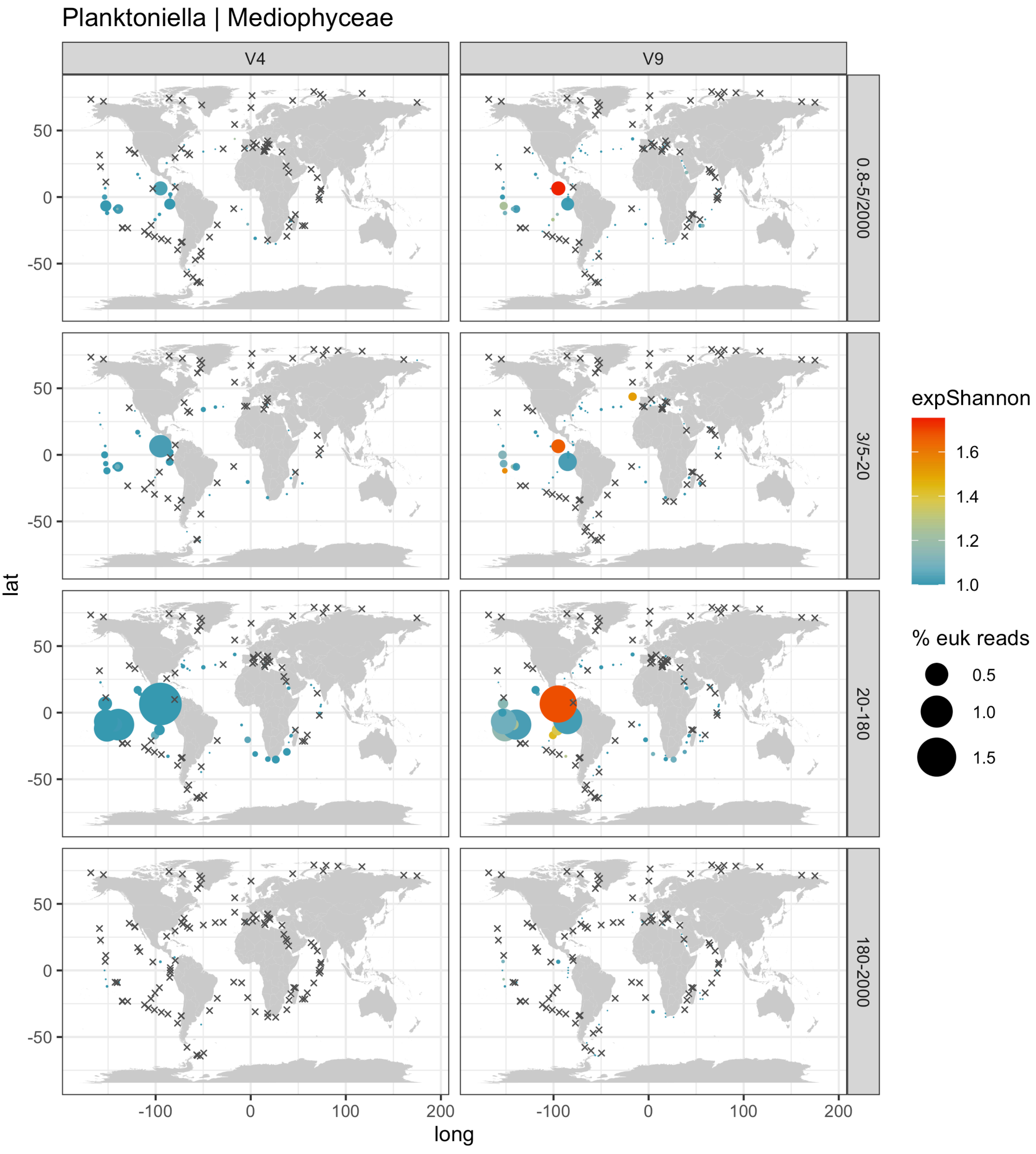

Planktoniella | Mediophyceae
V4
V9
0.8-5/2000
3/5-20
20-180
180-2000
lat
long
50
0
-50
-100
0
100
200
expShannon
1.6
1.4
1.2
1.0
% euk reads
0.5
1.0
1.5

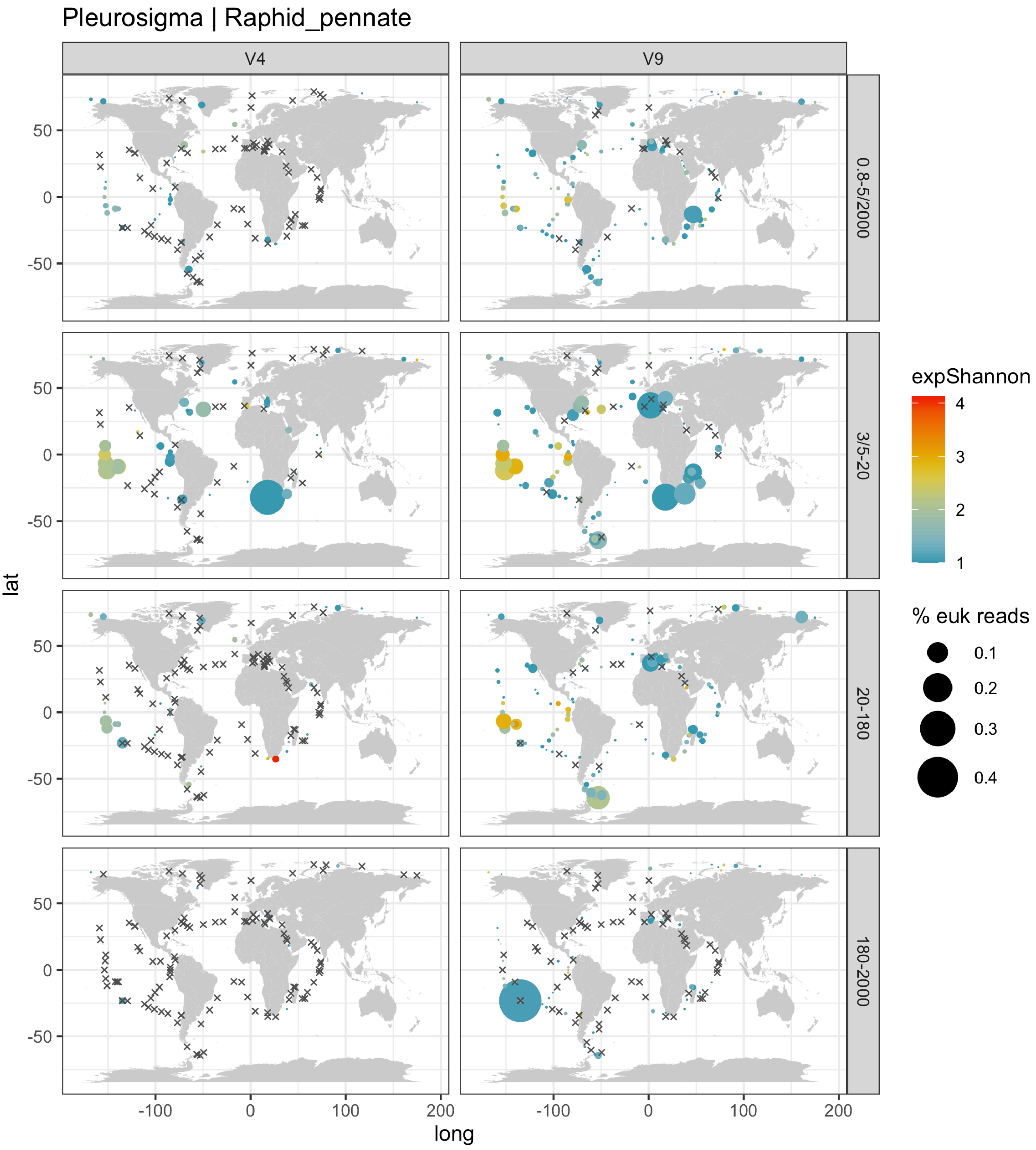

Pleurosigma | Raphid_pennate
V4
V9
0.8-5/2000
3/5-20
20-180
180-2000
lat
long
50
0
-50
-100
0
100
200
expShannon
4
3
2
1
% euk reads
0.1
0.2
0.3
0.4

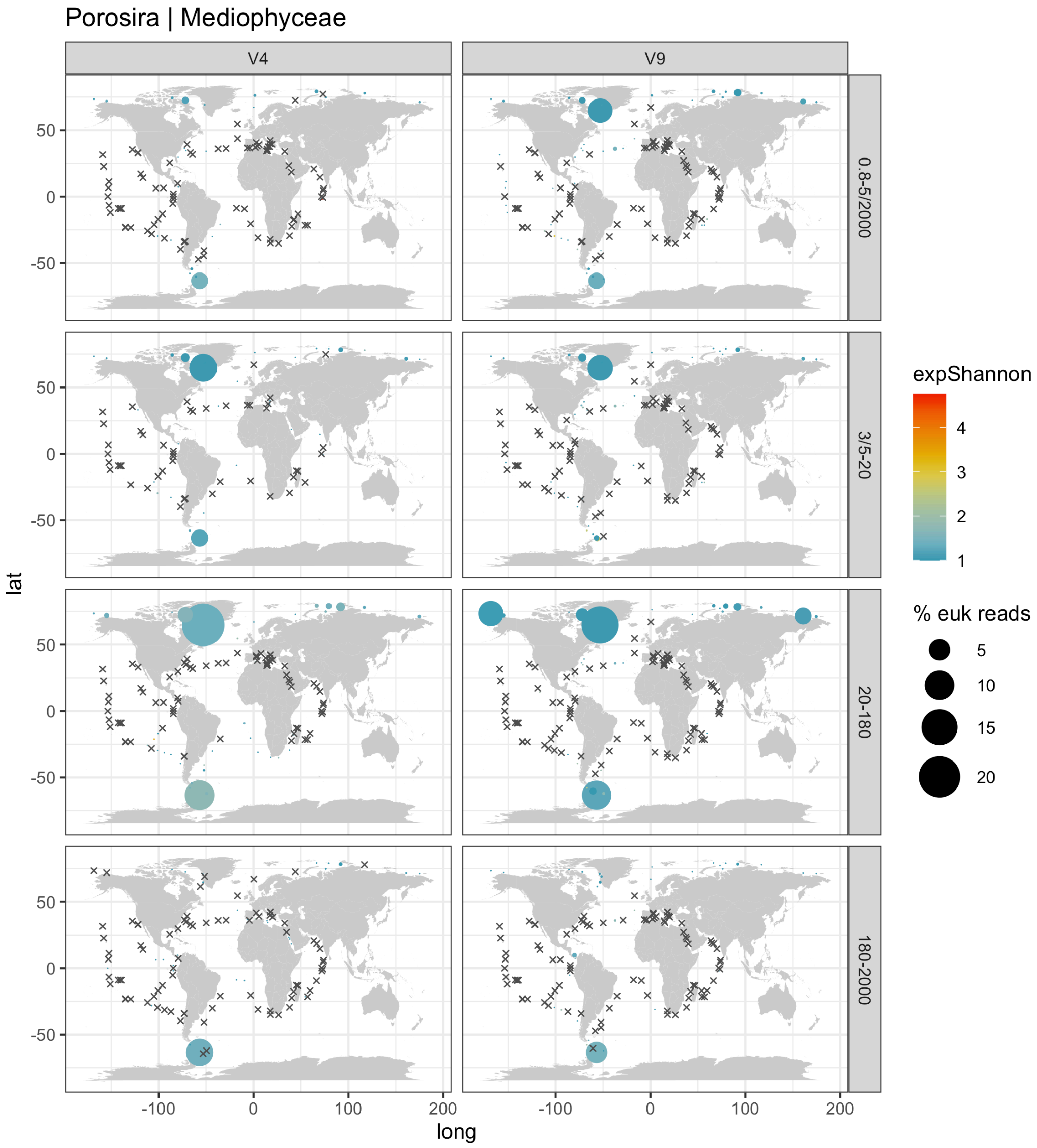
Porosira | Mediophyceae
V4
V9
0.8-5/2000
3/5-20
20-180
180-2000
lat
long
50
0
-50
-100
0
100
200
expShannon
4
3
2
1
% euk reads
5
10
15
20

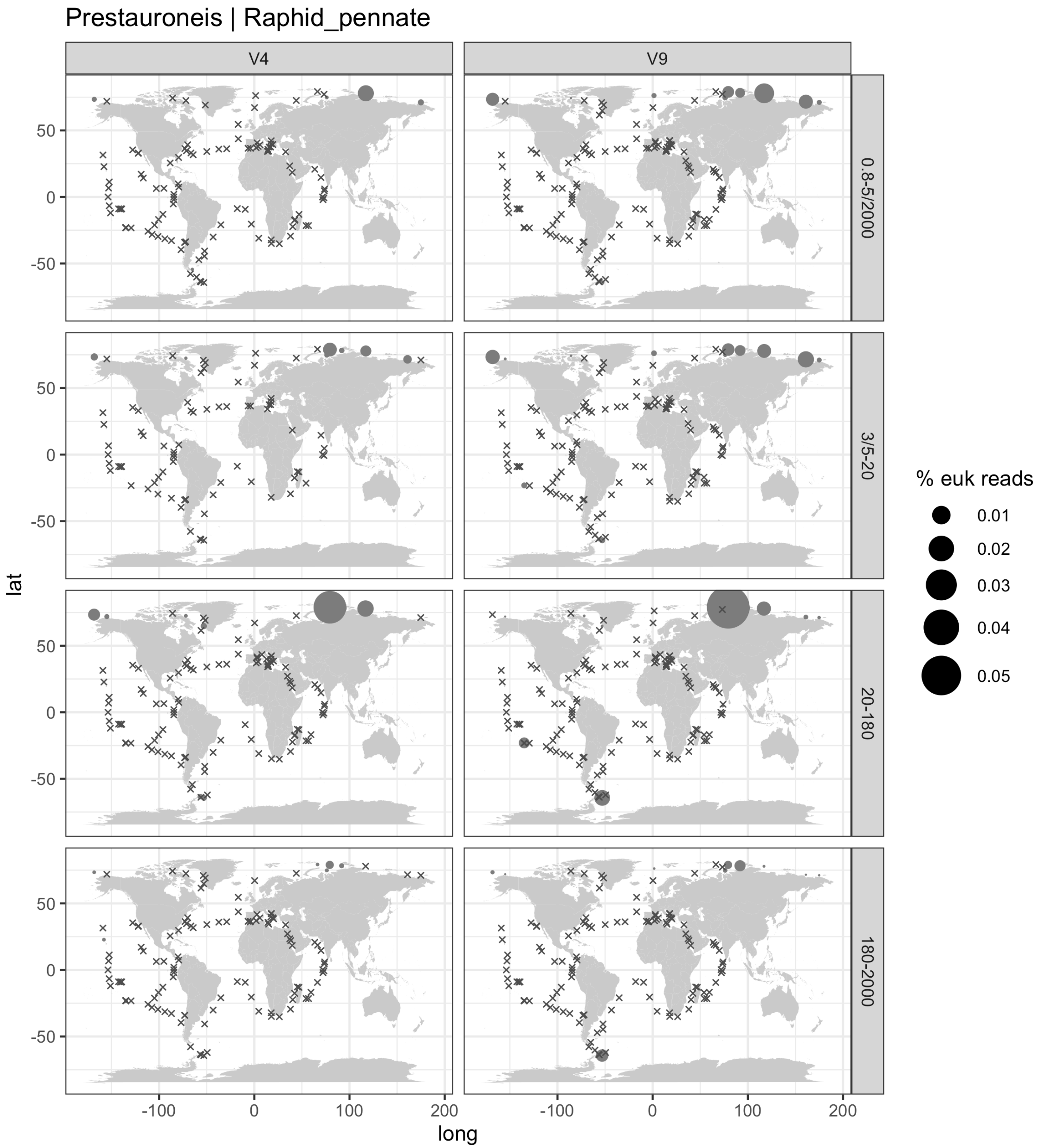

Prestauroneis | Raphid_pennate
V4
V9
0.8-5/2000
3/5-20
20-180
180-2000
lat
long
50
0
-50
-100
0
100
200
% euk reads
0.01
0.02
0.03
0.04
0.05

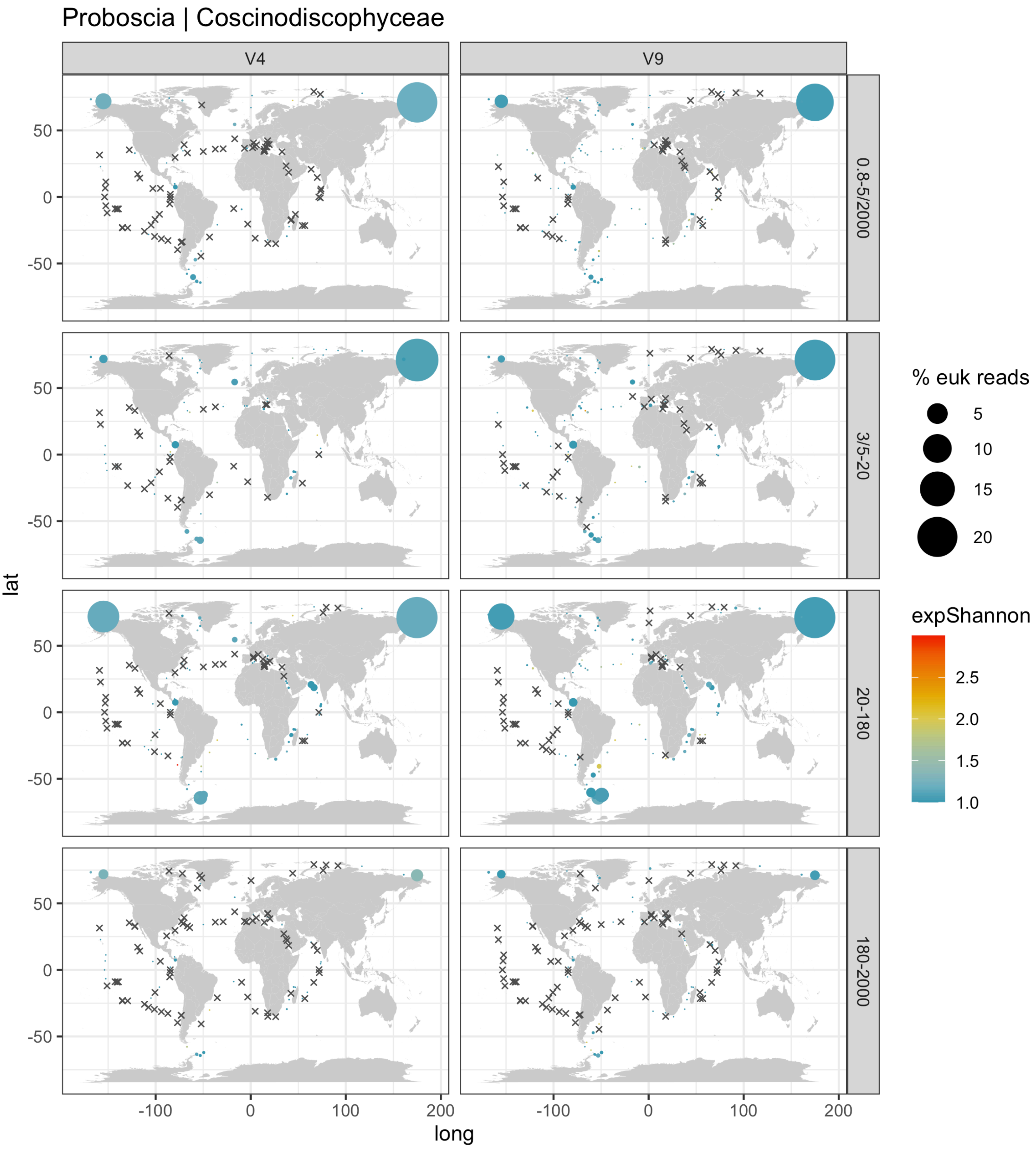

Proboscia | Coscinodiscophyceae
V4
V9
0.8-5/2000
3/5-20
20-180
180-2000
lat
long
50
0
-50
-100
100
200
% euk reads
5
10
15
20
expShannon
2.5
2.0
1.5
1.0

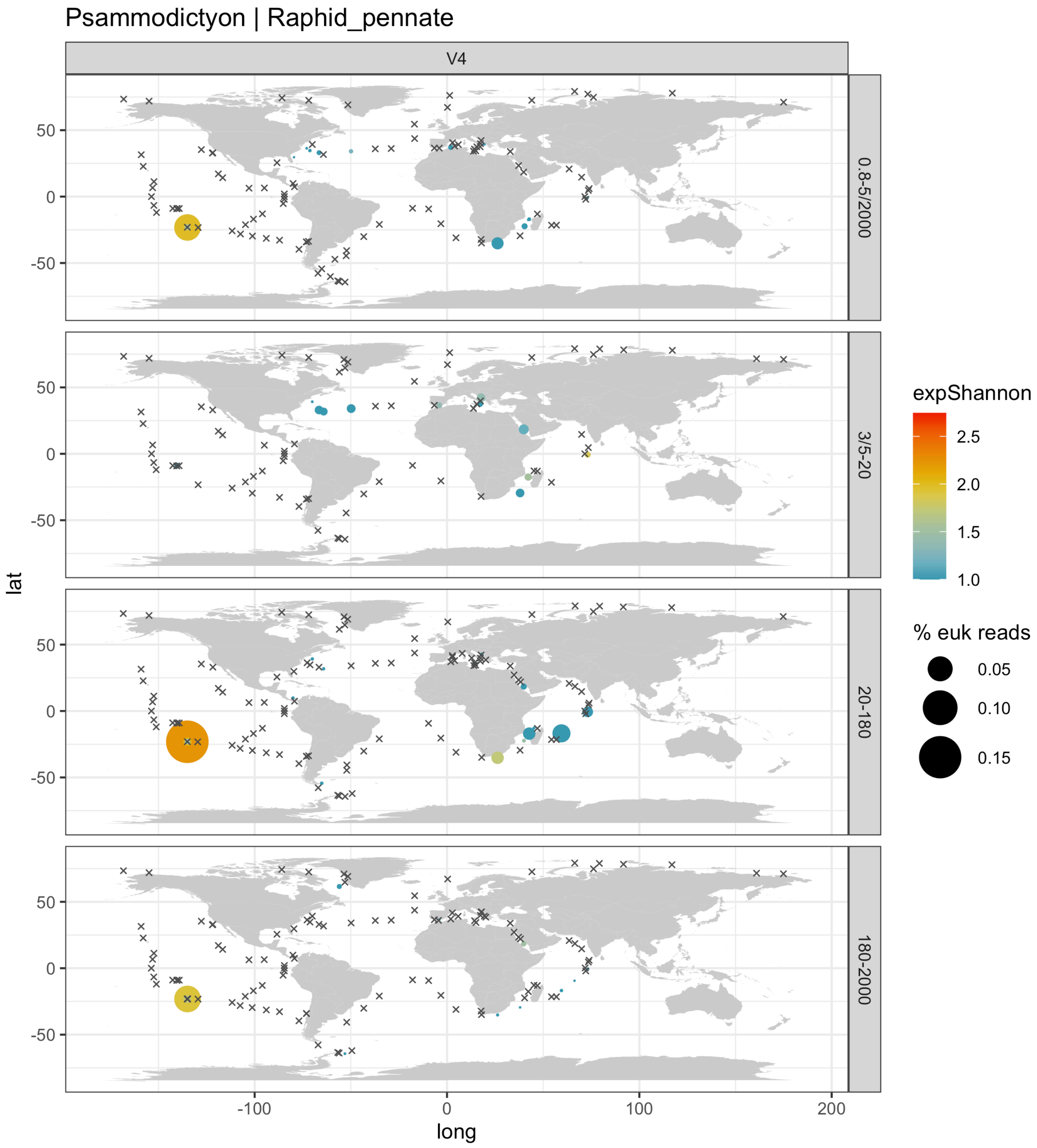

Psammodictyon | Raphid_pennate
V4
0.8-5/2000
3/5-20
20-180
180-2000
lat
long
50
0
-50
-100
0
100
200
expShannon
2.5
2.0
1.5
1.0
% euk reads
0.05
0.10
0.15

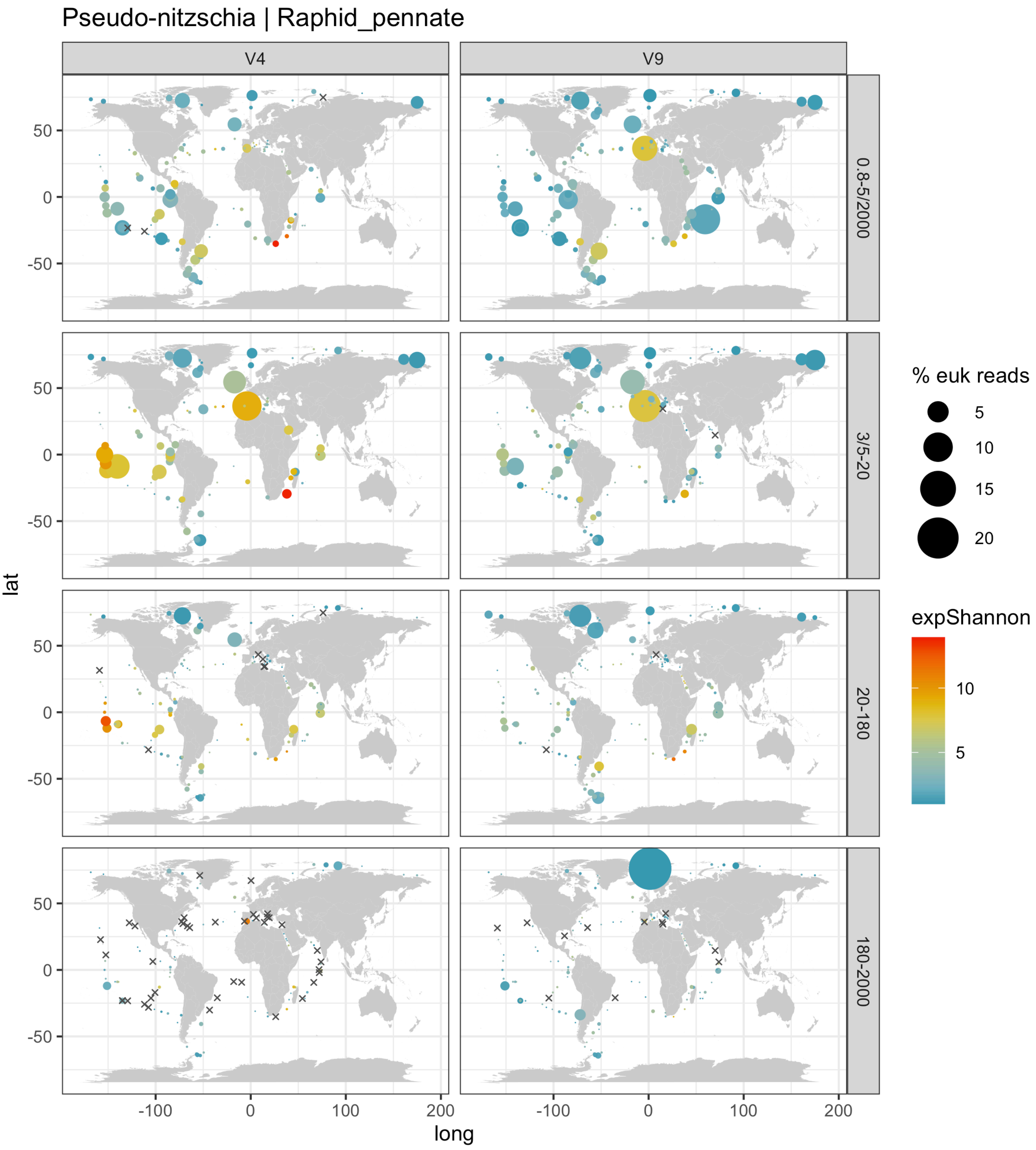
Pseudo-nitzschia | Raphid_pennate
V4
V9
0.8-5/2000
3/5-20
20-180
180-2000
lat
long
50
0
-50
-100
0
100
200
% euk reads
5
10
15
20
expShannon
10
5

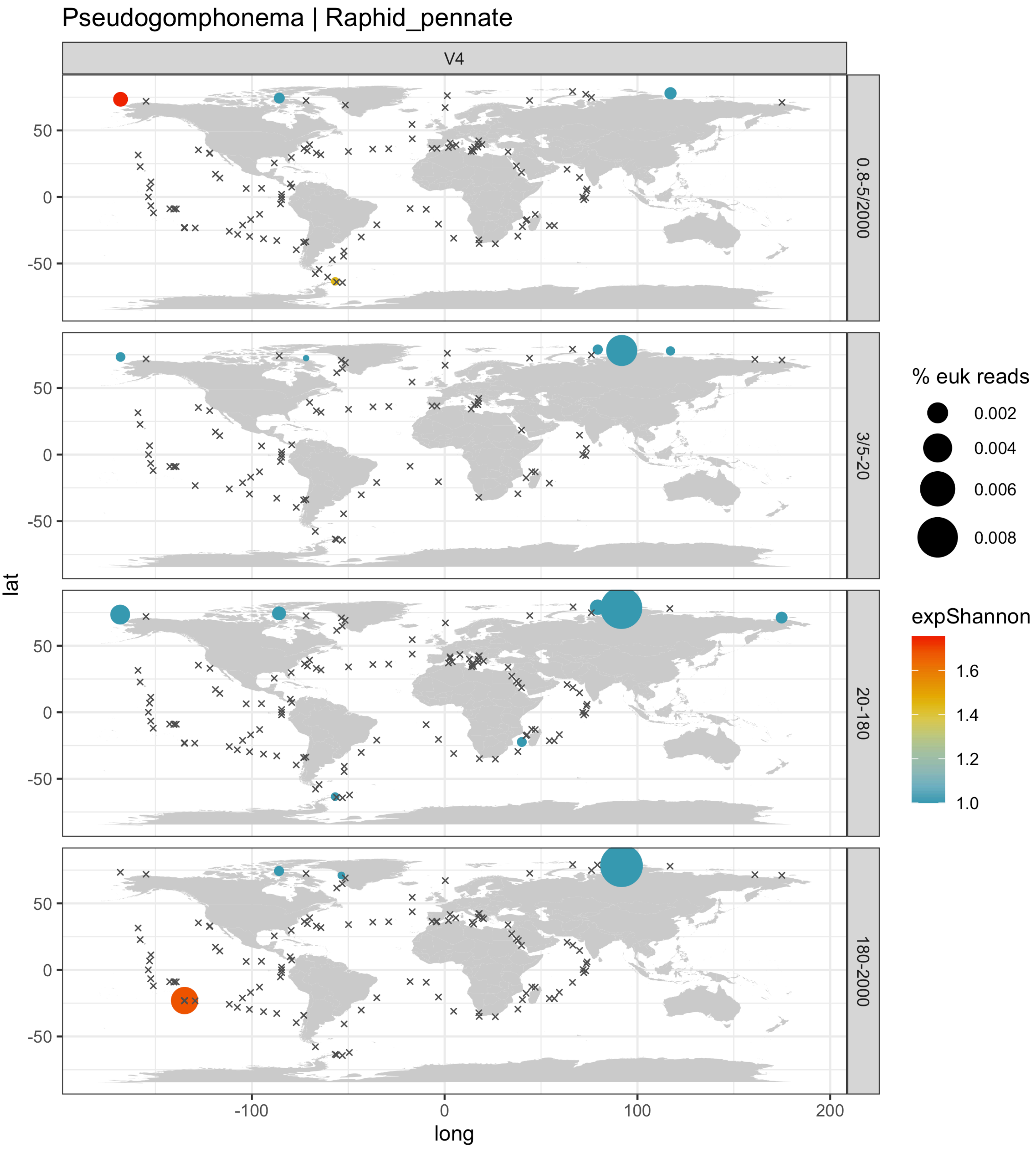

Pseudogomphonema | Raphid_pennate
V4
0.8-5/2000
3/5-20
20-180
180-2000
lat
long
-100
0
100
200
50
0
-50
% euk reads
0.002
0.004
0.006
0.008
expShannon
1.6
1.4
1.2
1.0

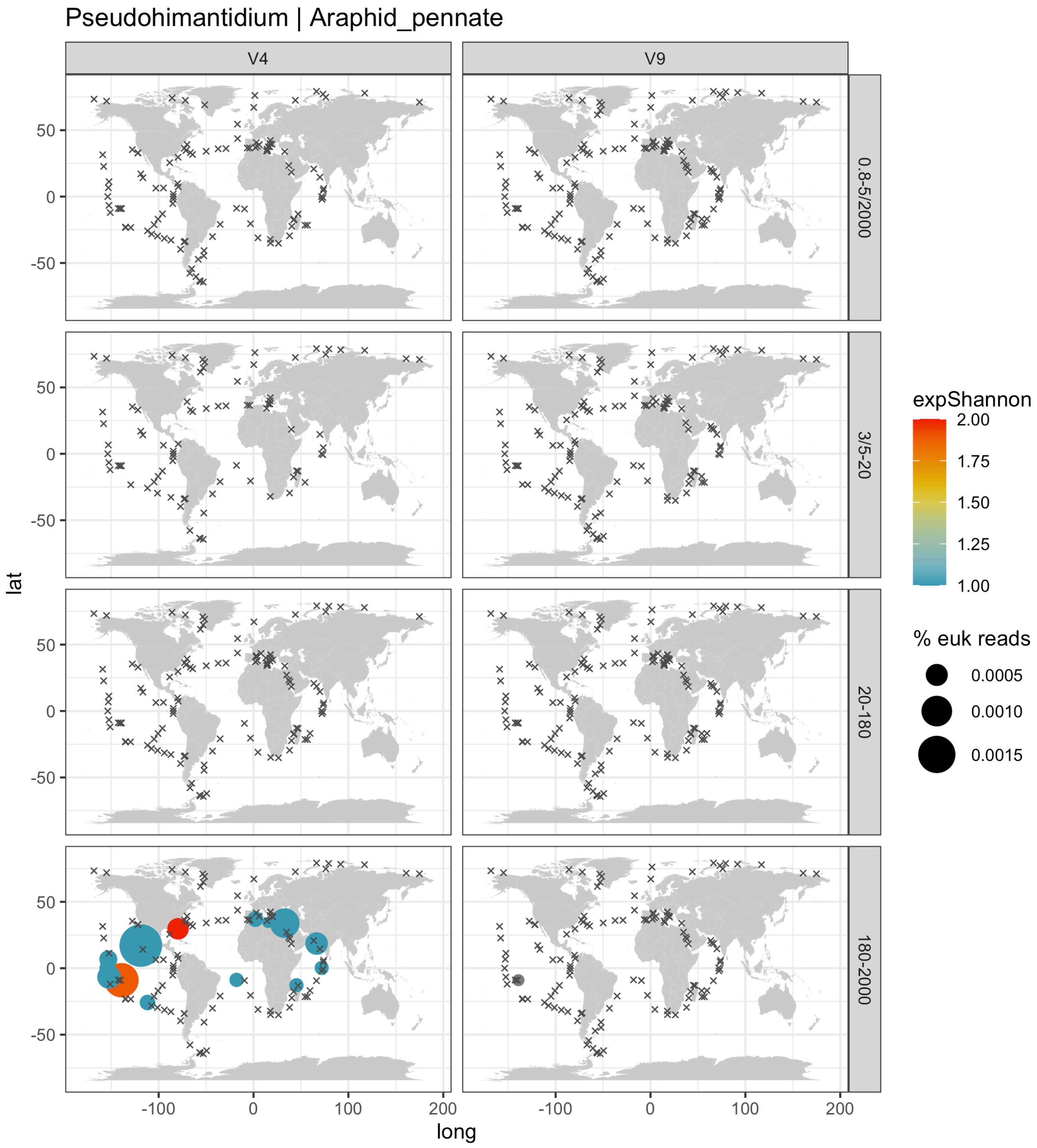

Pseudohimantidium | Araphid_pennate
V4
V9
0.8-5/2000
3/5-20
20-180
180-2000
lat
long
50
0
-50
-100
100
200
expShannon
2.00
1.75
1.50
1.25
1.00
% euk reads
0.0005
0.0010
0.0015

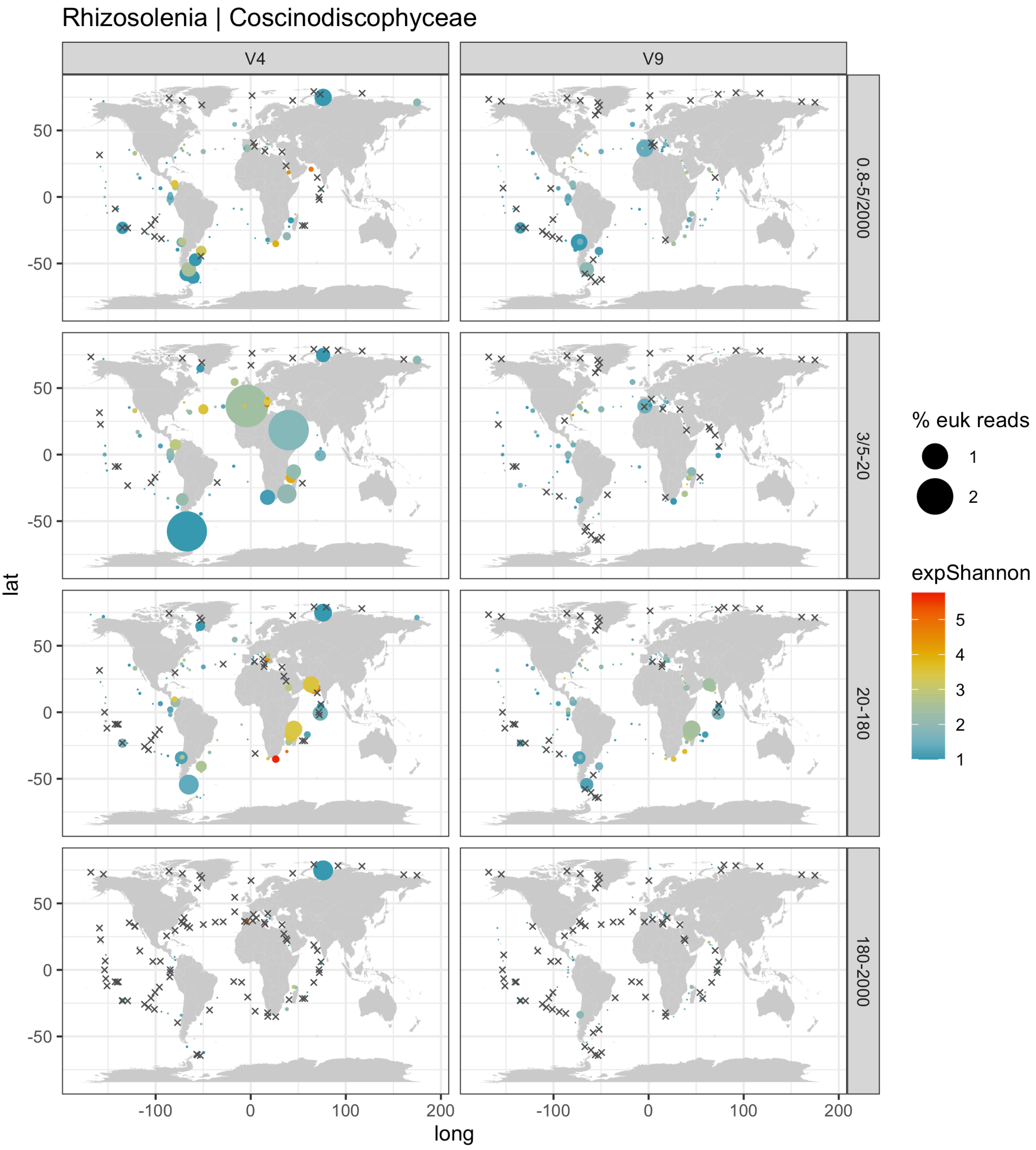

Rhizosolenia | Coscinodiscophyceae
V4
V9
0.8-5/2000
3/5-20
20-180
180-2000
lat
long
50
0
-50
-100
0
100
200
% euk reads
1
2
expShannon
5
4
3
2
1

Rossia | Raphid_pennate

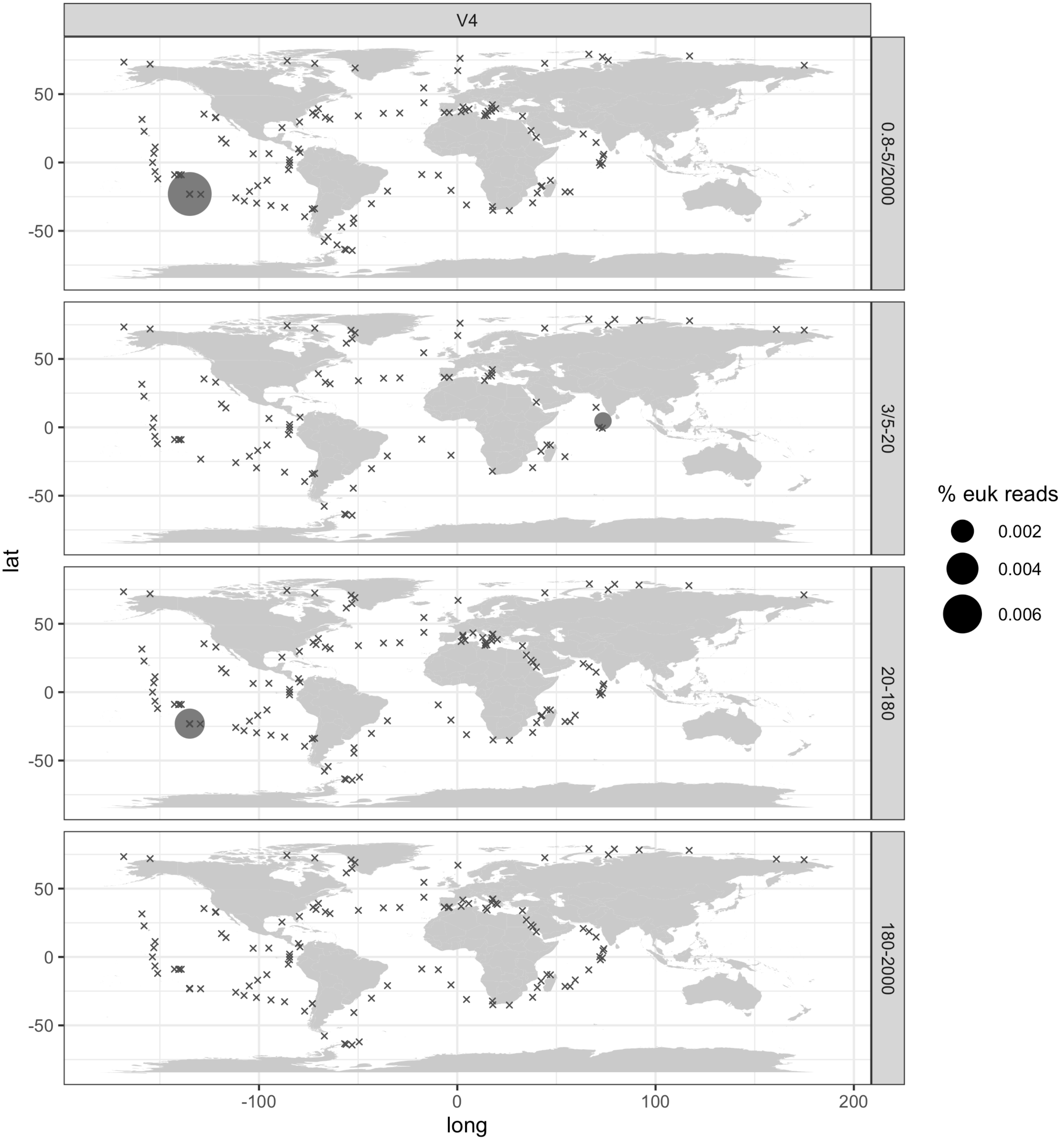

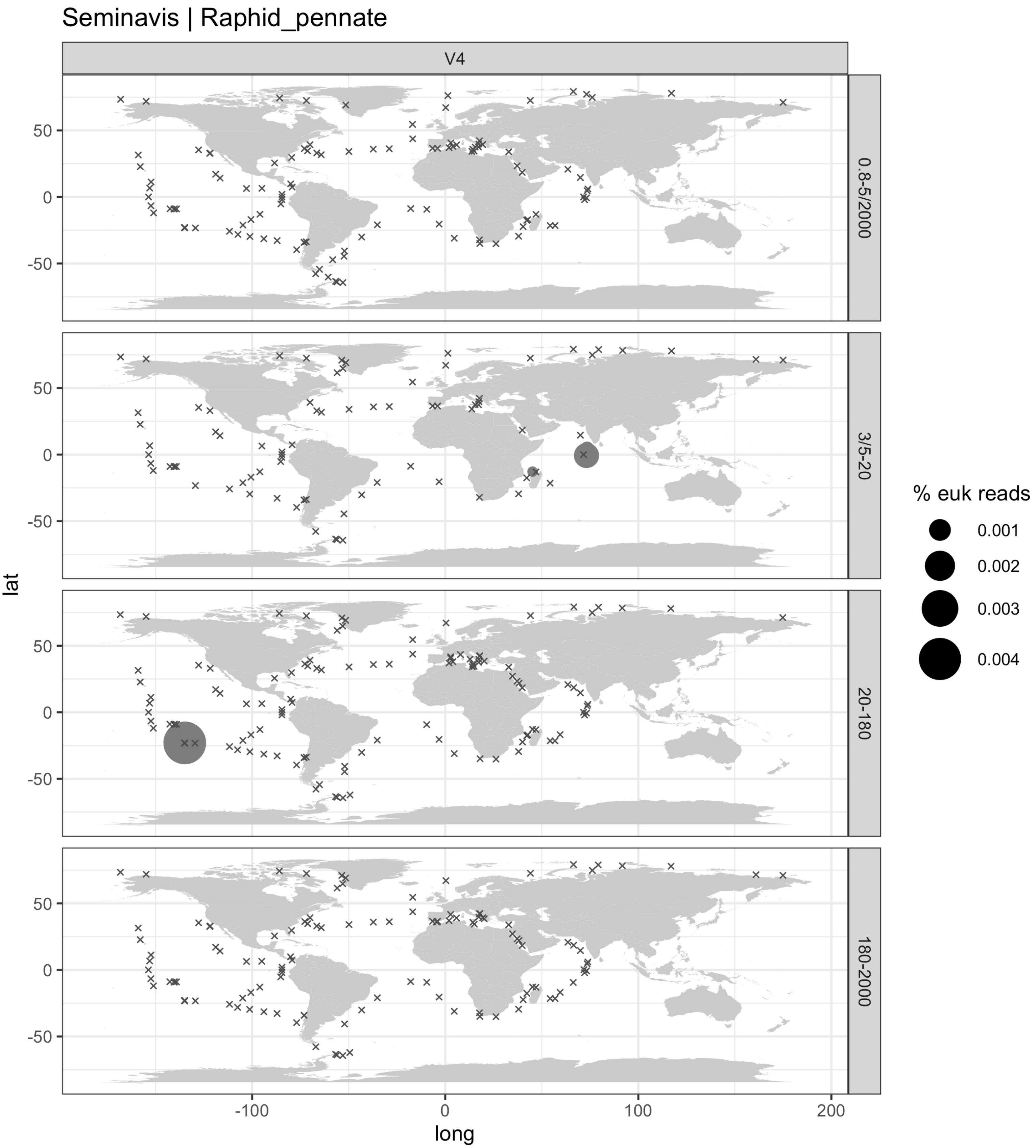
Seminavis | Raphid_pennate
V4
0.8-5/2000
3/5-20
20-180
180-2000
% euk reads
0.001
0.002
0.003
0.004
lat
long
50
0
-50
-100
0
100
200

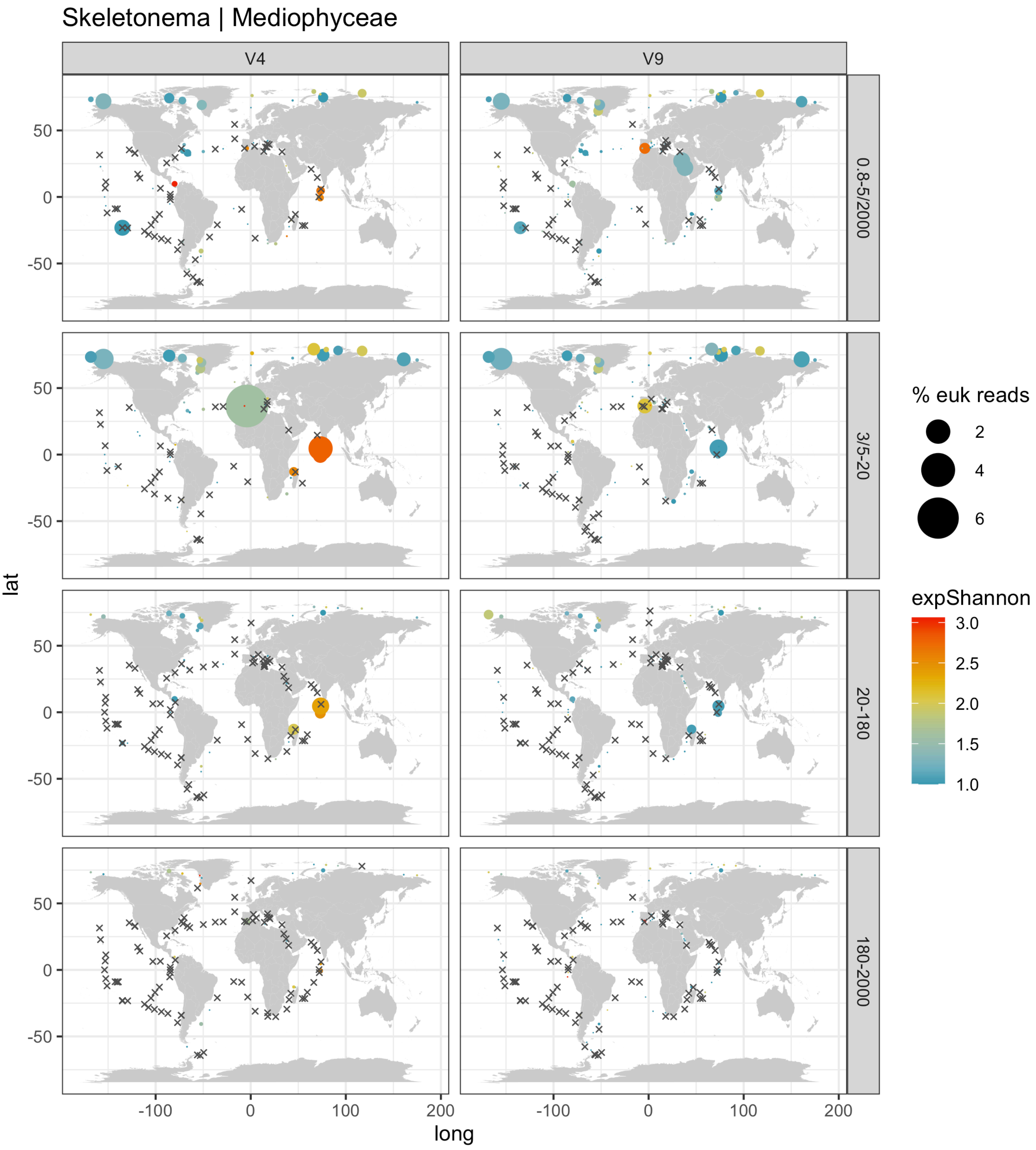

Skeletonema | Mediophyceae
V4
V9
0.8-5/2000
3/5-20
20-180
180-2000
lat
long
50
0
-50
-100
0
100
200
% euk reads
2
4
6
expShannon
3.0
2.5
2.0
1.5
1.0

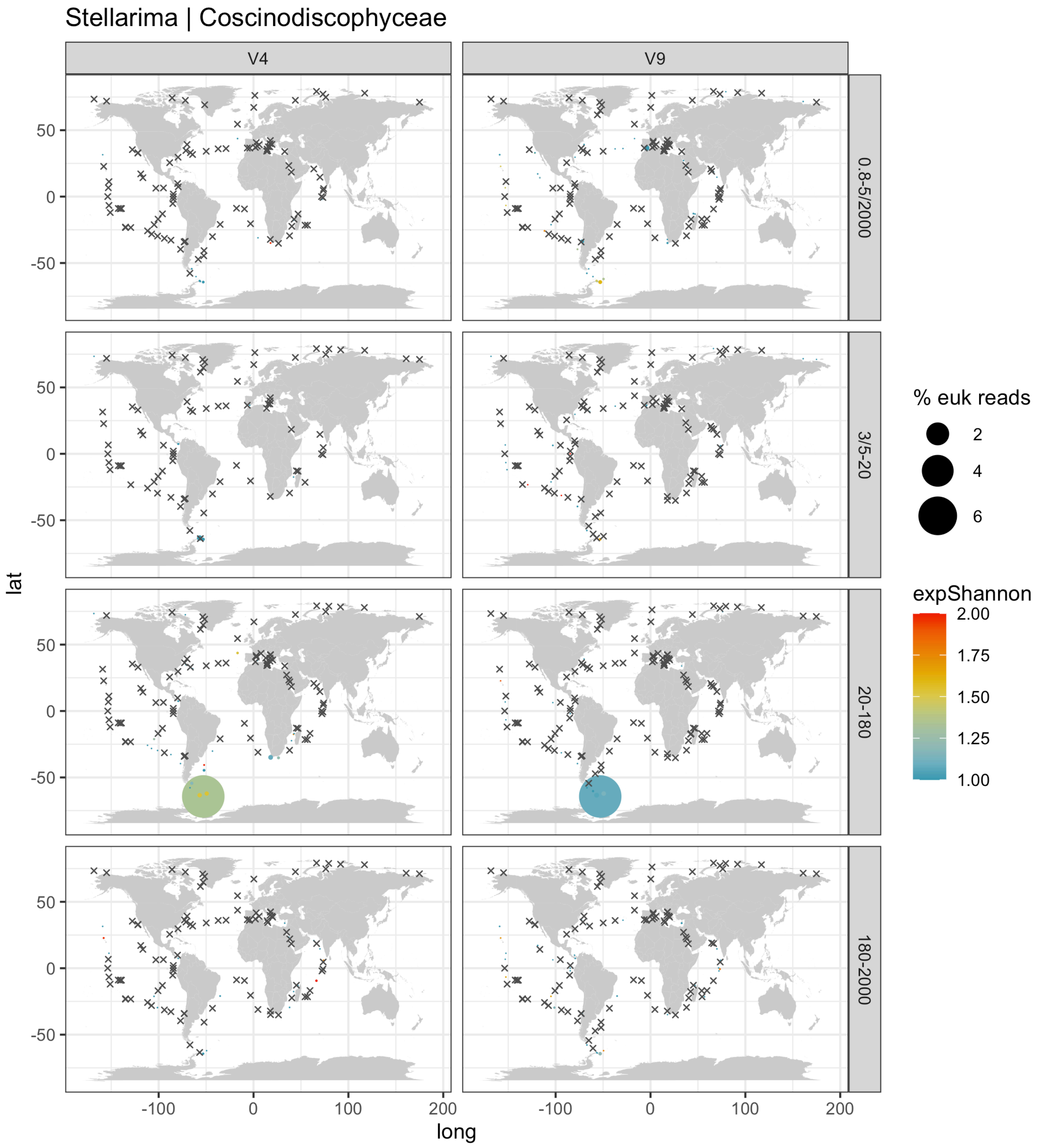

Stellarima | Coscinodiscophyceae
V4
V9
0.8-5/2000
3/5-20
20-180
180-2000
lat
long
% euk reads
2
4
6
expShannon
2.00
1.75
1.50
1.25
1.00

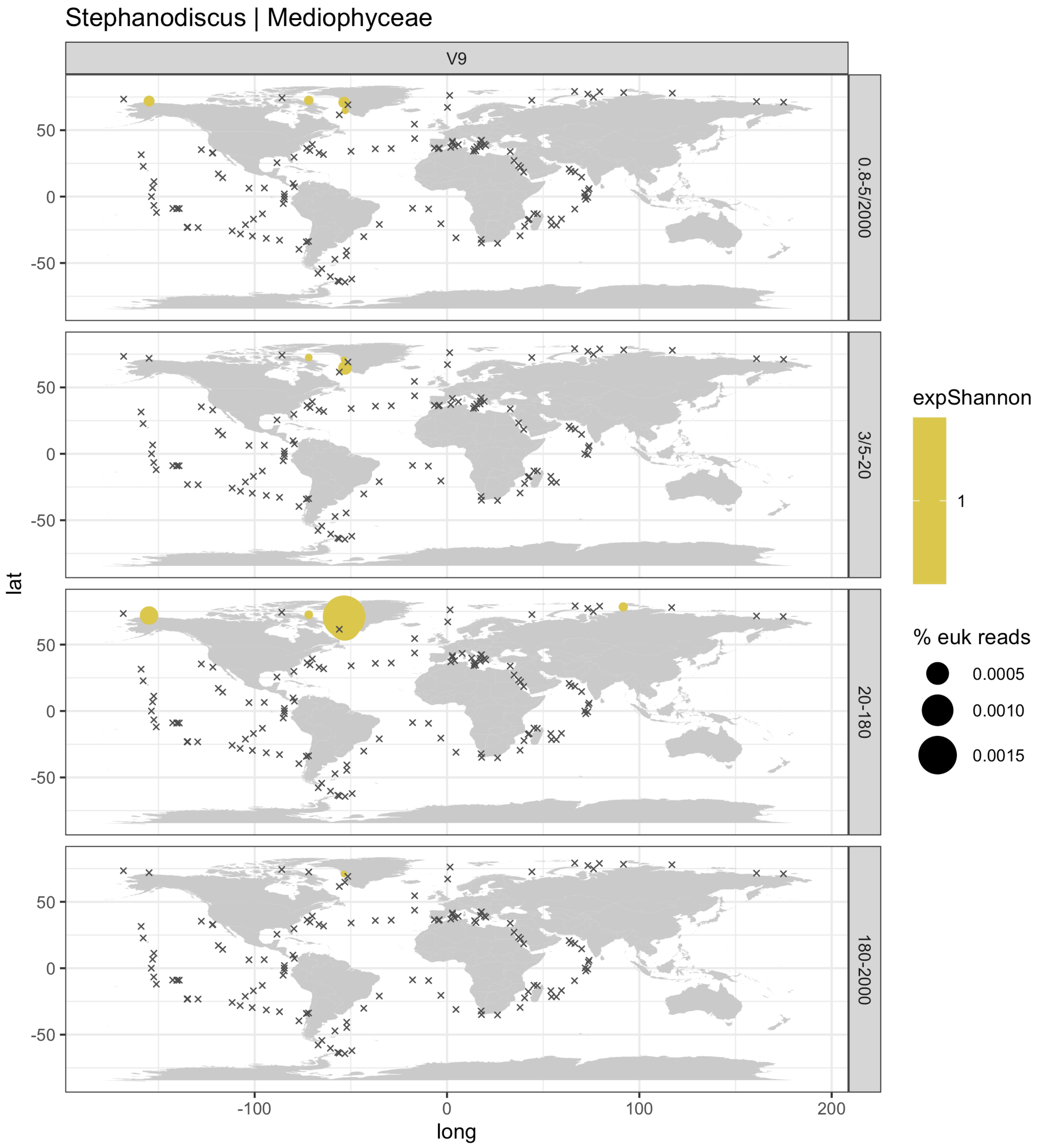

Stephanodiscus | Mediophyceae
V9
0.8-5/2000
3/5-20
20-180
180-2000
lat
long
50
0
-50
-100
0
100
200
expShannon
1
% euk reads
0.0005
0.0010
0.0015

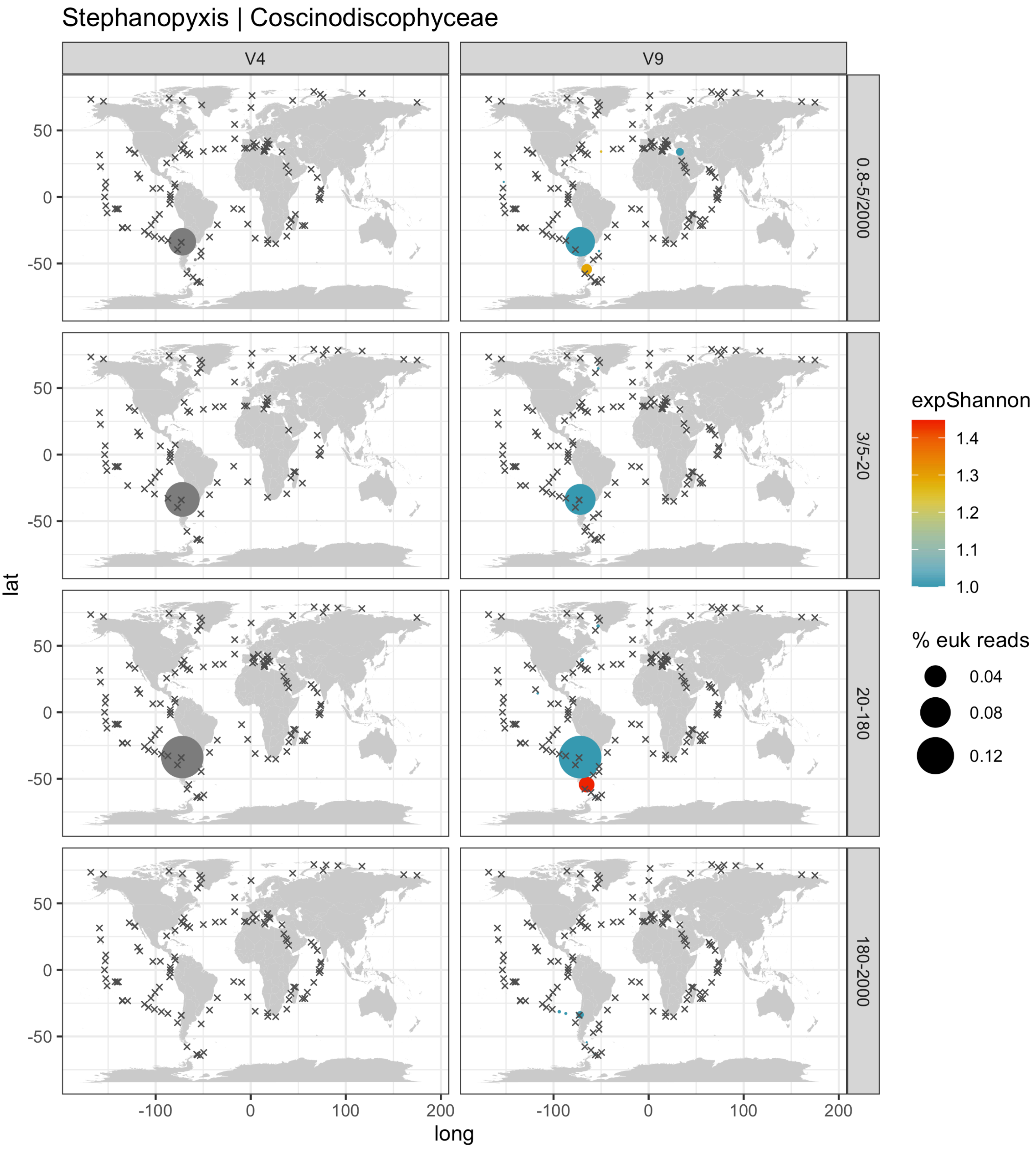

Stephanopyxis | Coscinodiscophyceae
V4
V9
0.8-5/2000
3/5-20
20-180
180-2000
lat
long
50
0
-50
-100
100
200
expShannon
1.4
1.3
1.2
1.1
1.0
% euk reads
0.04
0.08
0.12

Striatella | Araphid_pennate

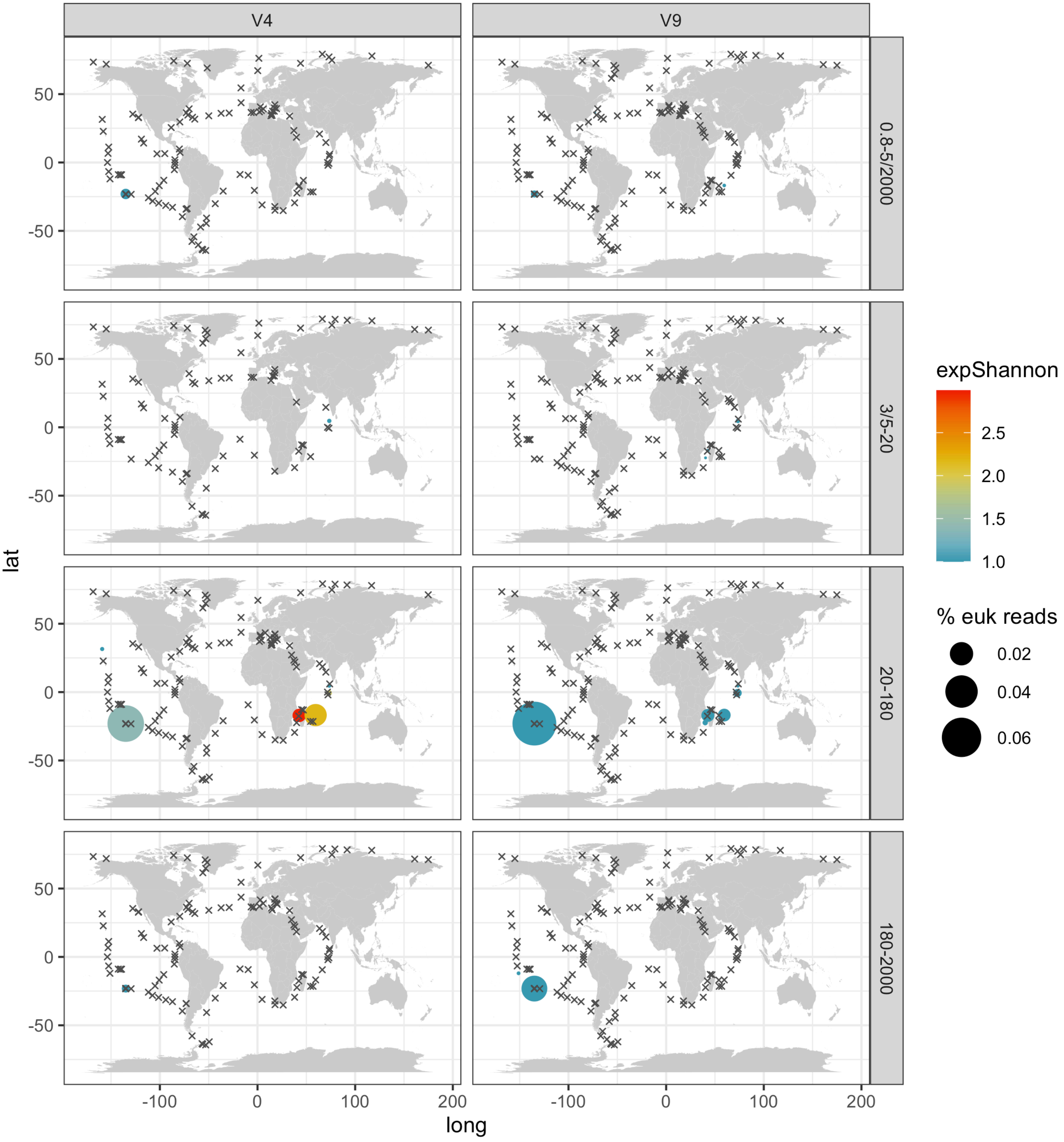

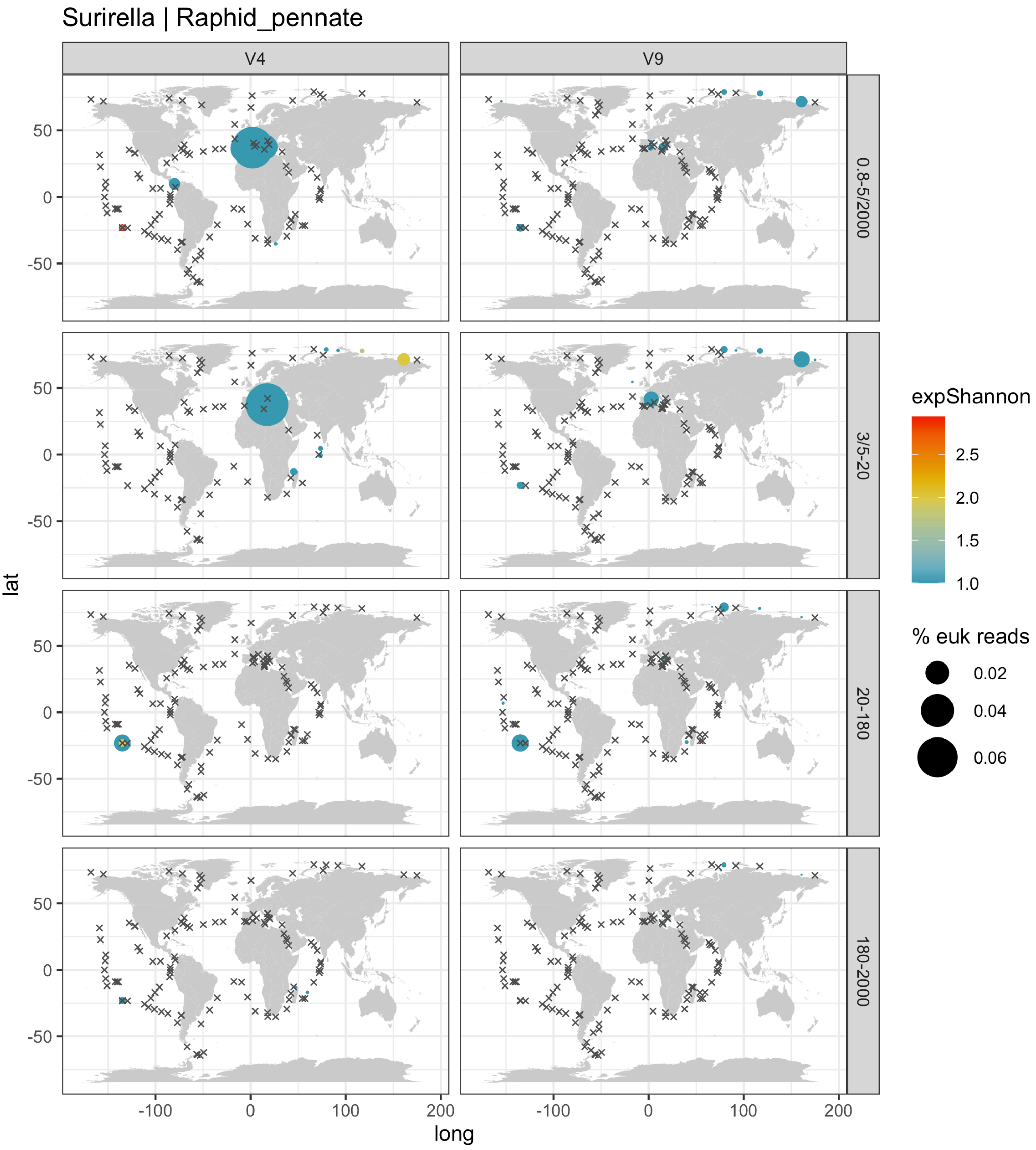

Surirella | Raphid_pennate
V4
V9
0.8-5/2000
3/5-20
20-180
180-2000
lat
long
50
0
-50
-100
100
200
expShannon
2.5
2.0
1.5
1.0
% euk reads
0.02
0.04
0.06

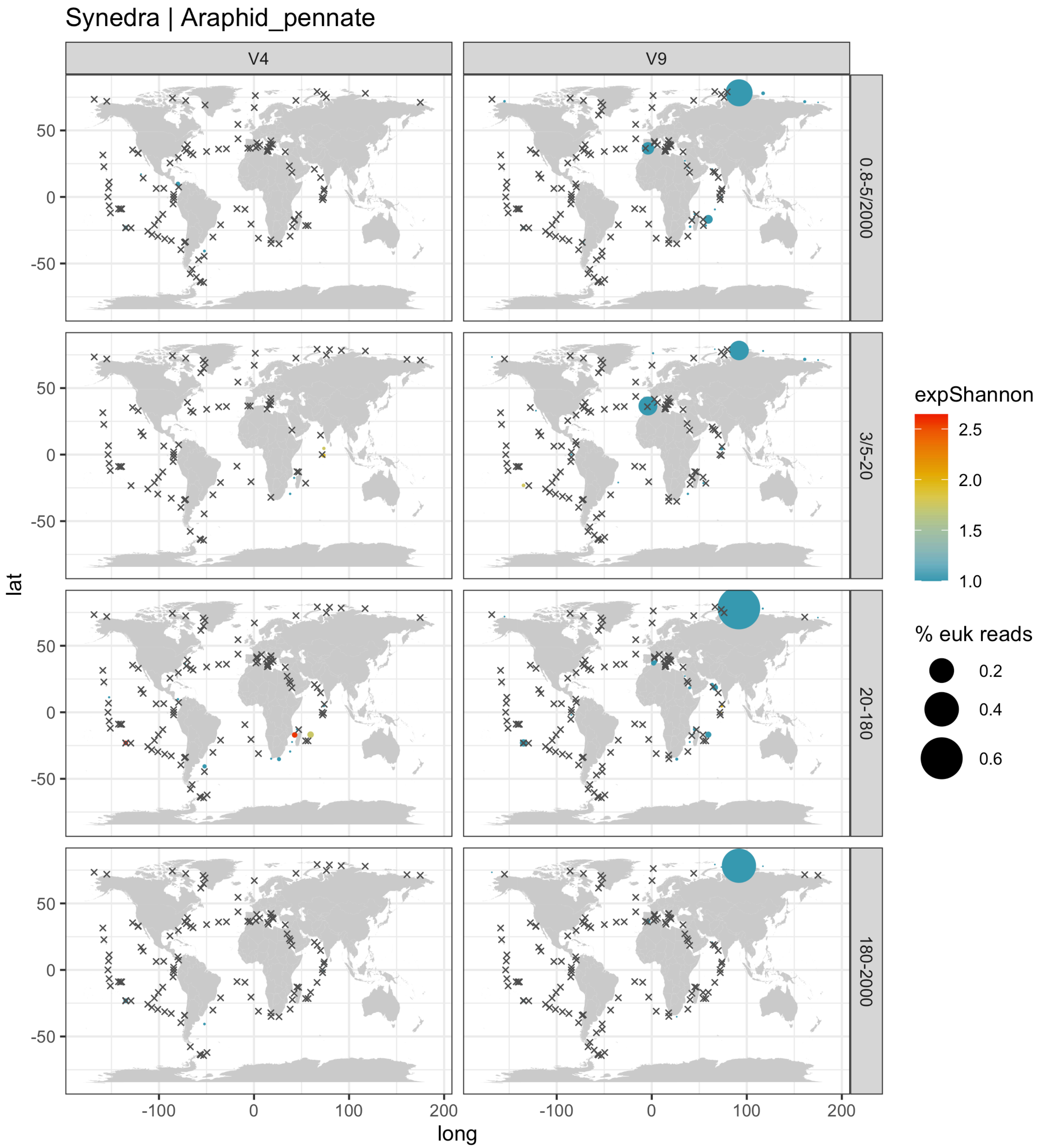

Synedra | Araphid_pennate
V4
V9
0.8-5/2000
3/5-20
20-180
180-2000
lat
long
50
0
-50
-100
100
200
expShannon
2.5
2.0
1.5
1.0
% euk reads
0.2
0.4
0.6

Talaroneis | Araphid_pennate

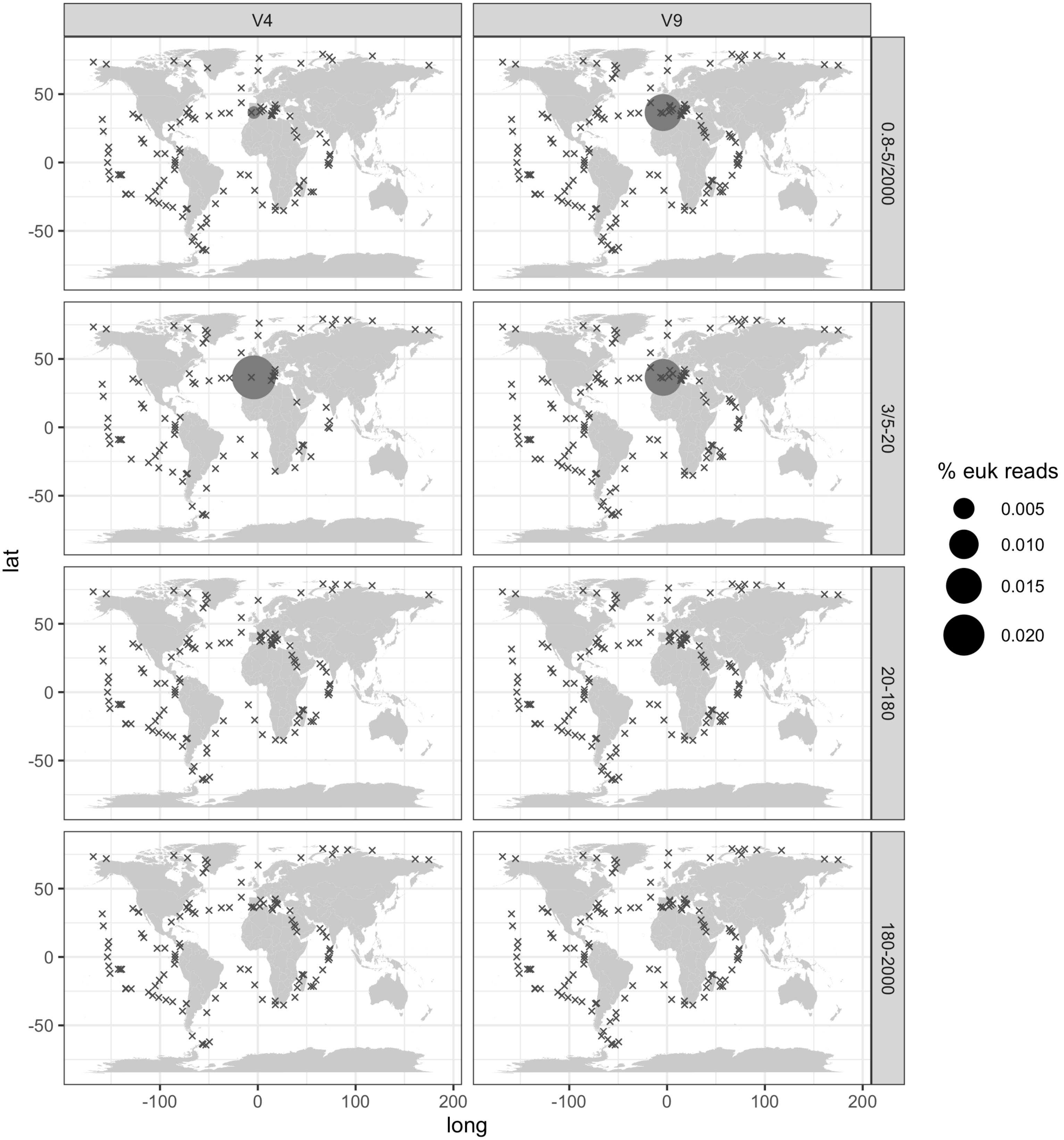

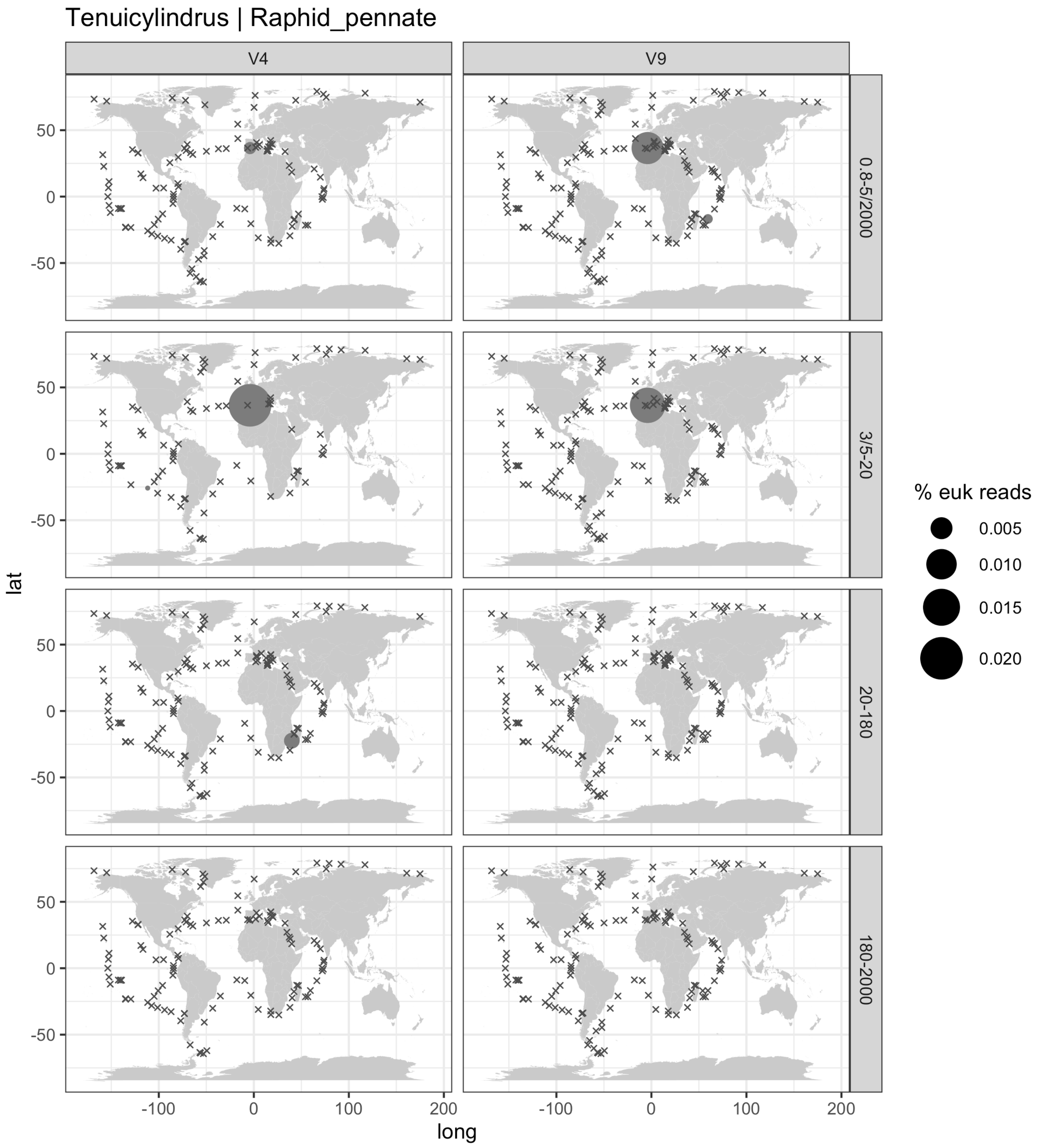

Tenuicylindrus | Raphid_pennate
V4
V9
0.8-5/2000
3/5-20
20-180
180-2000
lat
long
50
0
-50
-100
100
200
% euk reads
0.005
0.010
0.015
0.020

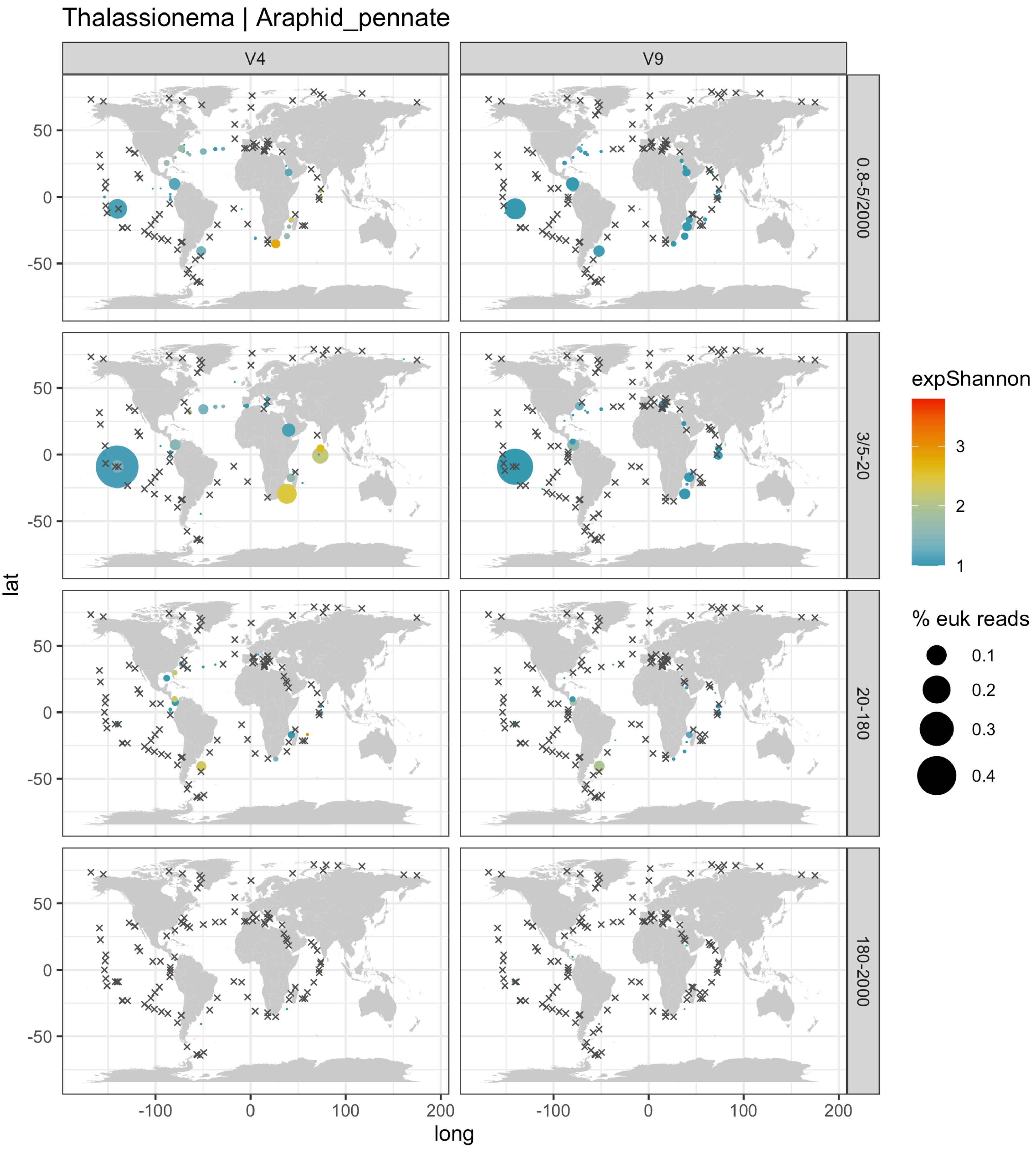

Thalassionema | Araphid_pennate
V4
V9
0.8-5/2000
3/5-20
20-180
180-2000
lat
long
50
0
-50
-100
100
200
expShannon
3
2
1
% euk reads
0.1
0.2
0.3
0.4

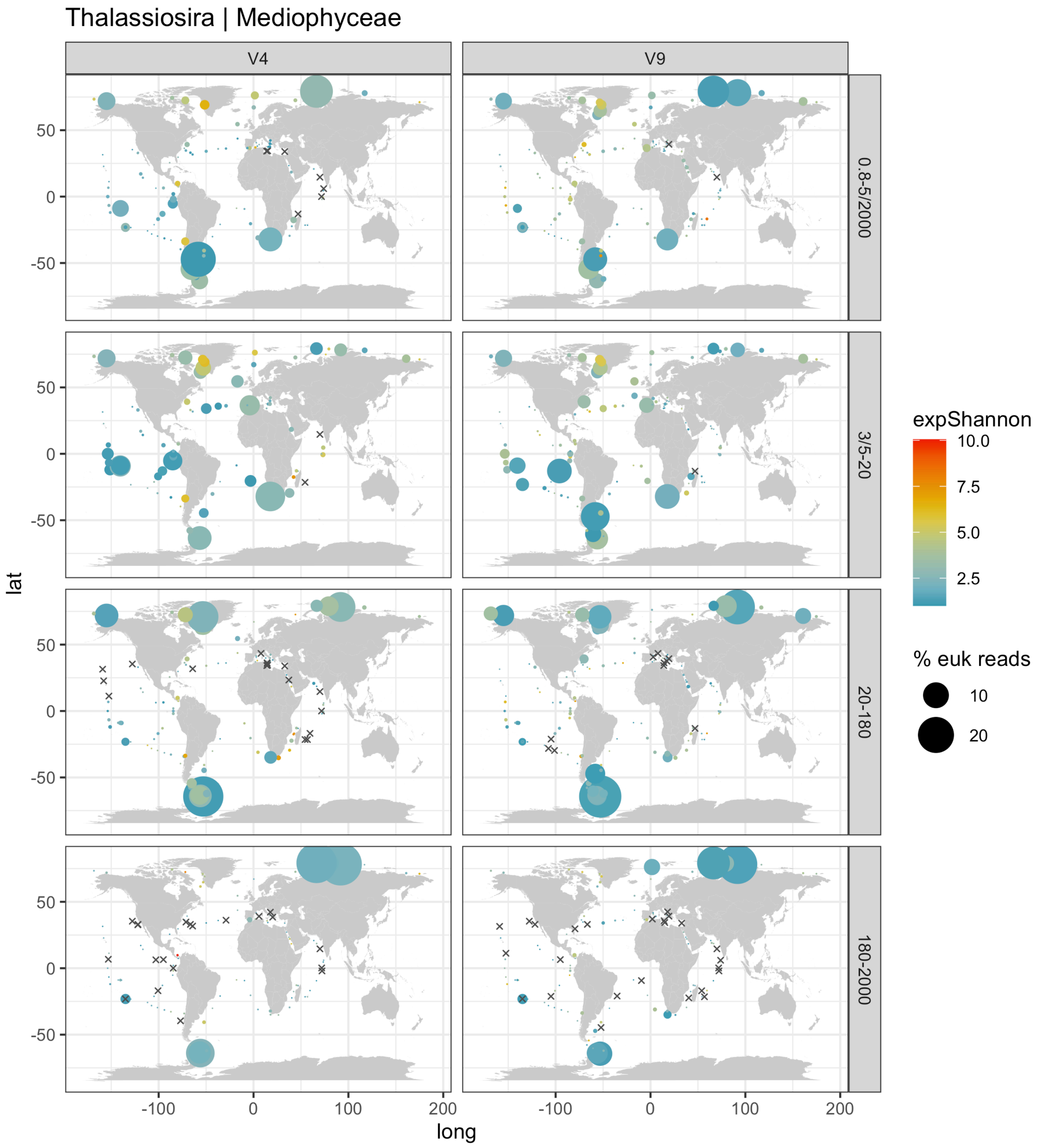

Thalassiosira | Mediophyceae
V4
V9
0.8-5/2000
3/5-20
20-180
180-2000
lat
long
50
0
-50
-100
0
100
200
expShannon
10.0
7.5
5.0
2.5
% euk reads
10
20

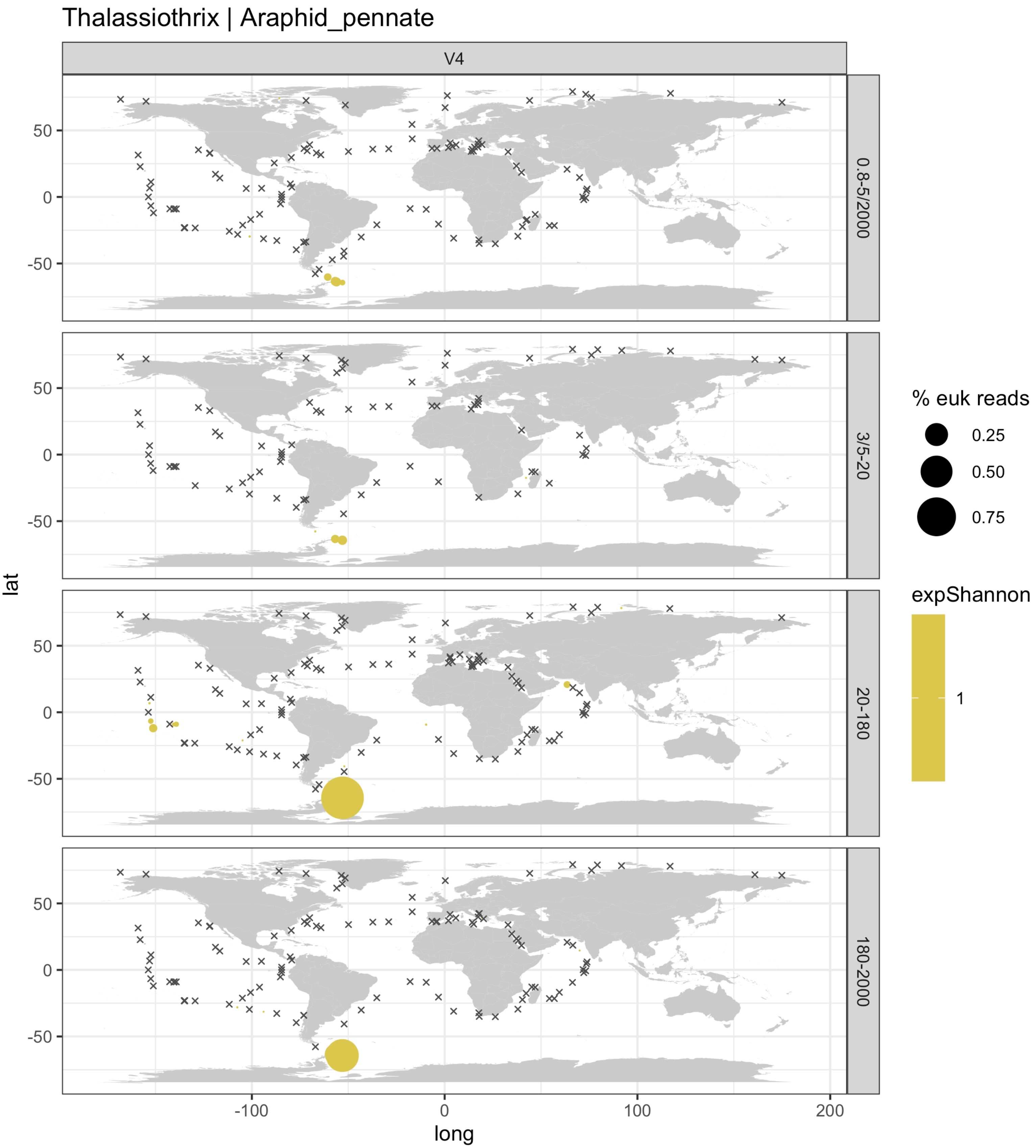
Thalassiothrix | Araphid_pennate
V4
0.8-5/2000
3/5-20
20-180
180-2000
lat
long
50
0
-50
-100
0
100
200
% euk reads
0.25
0.50
0.75
expShannon
1

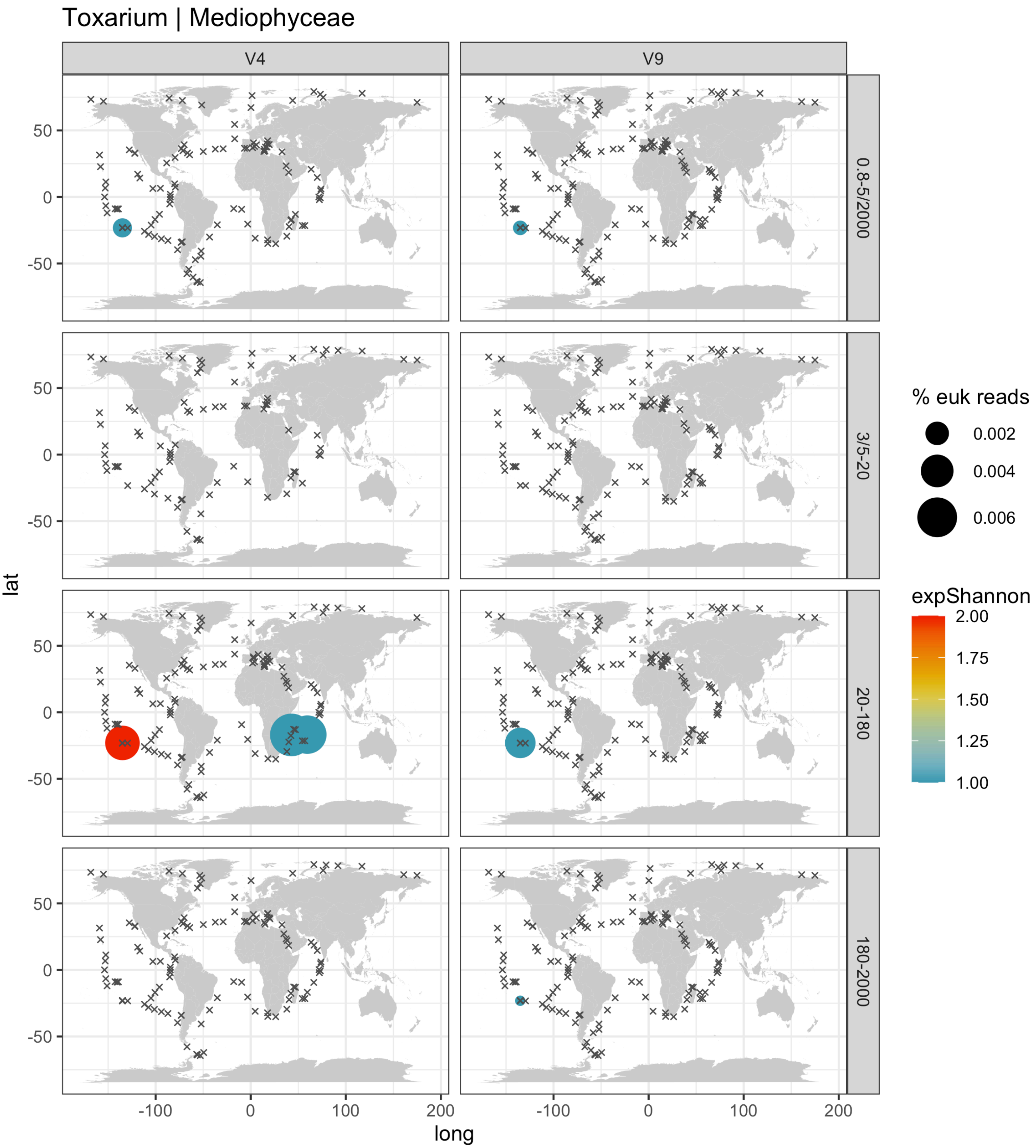

Toxarium | Mediophyceae
V4
V9
0.8-5/2000
3/5-20
20-180
180-2000
lat
long
% euk reads
0.002
0.004
0.006
expShannon
2.00
1.75
1.50
1.25
1.00

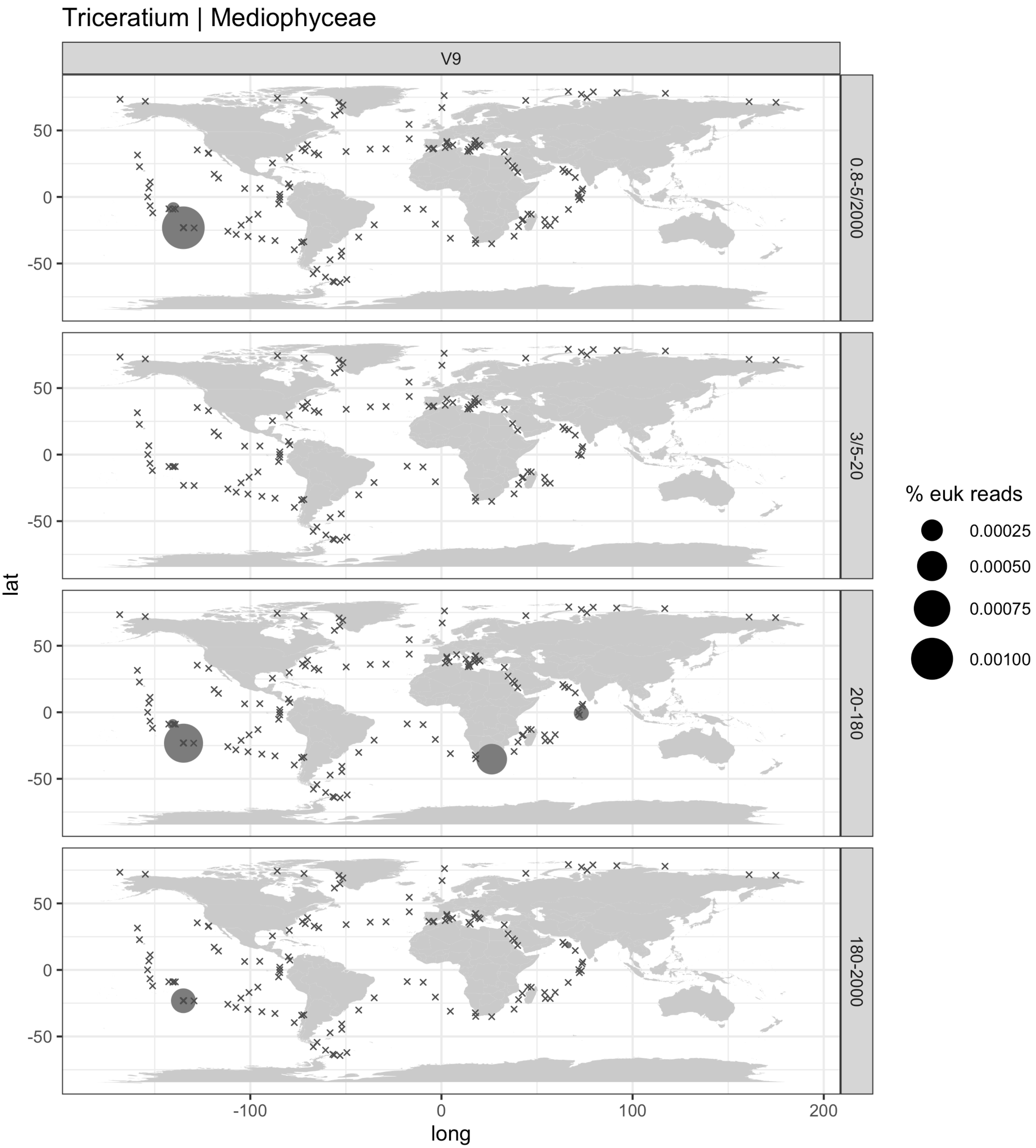

Triceratium | Mediophyceae
V9
0.8-5/2000
3/5-20
20-180
180-2000
lat
long
50
0
-50
-100
100
200
% euk reads
0.00025
0.00050
0.00075
0.00100

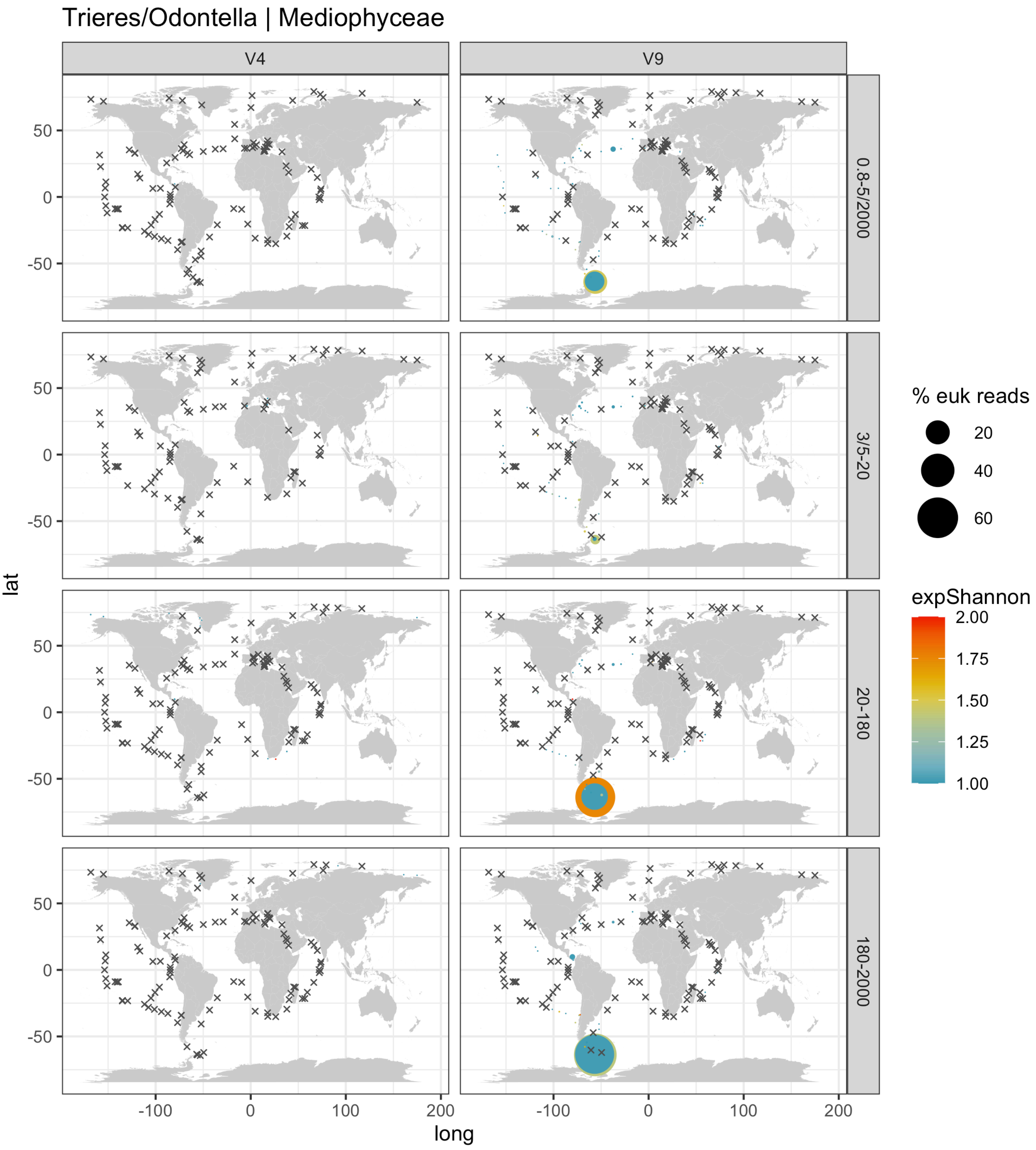

Trieres/Odontella | Mediophyceae
V4
V9
0.8-5/2000
3/5-20
20-180
180-2000
lat
long
50
0
-50
-100
0
100
200
% euk reads
20
40
60
expShannon
2.00
1.75
1.50
1.25
1.00
